# The *Gillenia trifoliata* genome reveals dynamics correlated with growth and reproduction in Rosaceae

**DOI:** 10.1038/s41438-021-00662-4

**Published:** 2021-11-01

**Authors:** Hilary S. Ireland, Chen Wu, Cecilia H. Deng, Elena Hilario, Ali Saei, Sylvia Erasmuson, Ross N. Crowhurst, Karine M. David, Robert J. Schaffer, David Chagné

**Affiliations:** 1 The New Zealand Institute for Plant and Food Research Ltd, Private Bag 92196, Auckland Mail Centre, Auckland, 1142 New Zealand; 2 School of Biological Sciences, The University of Auckland, Private Bag 92019, Auckland Mail Centre, Auckland, 1142 New Zealand; 3 Genomics Aotearoa, ℅ Department of Biochemistry, University of Otago, PO Box 56, Dunedin, 9054 New Zealand; 4 The New Zealand Institute for Plant and Food Research Ltd, Private Bag 11600, Palmerston North, 4442 New Zealand; 5 The New Zealand Institute for Plant and Food Research Ltd, Private Bag 4704, Christchurch Mail Centre, Christchurch, 8140 New Zealand; 6 The New Zealand Institute for Plant and Food Research Ltd, 55 Old Mill Road, RD 3, Motueka, 7198 New Zealand

**Keywords:** Comparative genomics, Genome duplication

## Abstract

The Rosaceae family has striking phenotypic diversity and high syntenic conservation. *Gillenia trifoliata* is sister species to the Maleae tribe of apple and ~1000 other species. *Gillenia* has many putative ancestral features, such as herb/sub-shrub habit, dry fruit-bearing and nine base chromosomes. This coalescence of ancestral characters in a phylogenetically important species, positions *Gillenia* as a ‘rosetta stone’ for translational science within Rosaceae. We present genomic and phenological resources to facilitate the use of *Gillenia* for this purpose. The *Gillenia* genome is the first fully annotated chromosome-level assembly with an ancestral genome complement (*x* = 9), and with it we developed an improved model of the Rosaceae ancestral genome. MADS and NAC gene family analyses revealed genome dynamics correlated with growth and reproduction and we demonstrate how *Gillenia* can be a negative control for studying fleshy fruit development in Rosaceae.

The Rosaceae family displays wide phenotypic diversity especially with regard to fleshy fruit forms^1^. All fleshy fruiting crops putatively arose from dry-fruiting ancestors^2^ and although most of the 3000 species within Rosaceae are dry-fruiting, they can be distilled down to two types, achenes and follicles, thus providing a common basis for comparative analyses. Comparisons between dry and fleshy fruiting relatives provide an alternative approach to study fruit development. Comparative genomics of almond and peach highlighted the potential of the transposable element landscape to alter the fate of fruit tissues^3^ and a comparison of woodland (*Fragaria vesca*) and pink barren (*Potentilla micrantha*) strawberries underscored the importance of MADS-box regulation of embryo and seed development for flesh development^4^. Despite the economic importance of pome fruits (*Malus domestica* (apple) and *Pyrus spp*. (pear)), no closely related dry-fruiting model is currently available.

Apple and pear belong to the Maleae tribe. The origin of this tribe proposes that ~50 Mya an ancestral species with *x* = 9 underwent autopolyploidisation, aneuploidy, and diploidisation to give rise to apple and pear (2*n* = 2*x* = 34) and sister taxa^2,5–7^. It is proposed that the ancestral species was similar to extant genus *Gillenia*. The genome of *Gillenia* remained unduplicated (2*n* = 2*x* = 18) and therefore provides a simpler foundation to untangle complexities of Maleae whole-genome duplication (WGD) as well as to improve prediction of genome configurations of the Rosaceae common ancestor.

The phenotype and genome of *G. trifoliata* has potential to provide a ‘rosetta stone’ for translational science within Rosaceae. The genome complement of *G. trifoliata* (*x* = 9) is similar to most Rosaceae species (*x* = 7, 8, 9) and represents the chromosome complement of the putative ancestor to all Rosaceae^8–11^. Currently no chromosome-level, annotated assemblies are available within Rosaceae with ancestral genome complement (*x* = 9), as *Dryas drummondii* and *Purshia tridentata* are both unannotated drafts^12^. All high-quality Rosaceae genomes sequenced to date are for either fleshy-fruited species or dry achene-fruiting species with fleshy accessories. A sequenced genome for a dry follicle-fruiting species, the ancestral fruit type of the second-largest subfamily Amygdaloideae (e.g. apple, peach, cherry), is currently unavailable. The growth habit of the largest Rosaceae subfamily, Rosoideae, is predominantly herbaceous, with some form of asexual propagation such as rhizomes, stolons, runners or suckers^2^. The rhizomatous annual-fruiting perennial herb or sub-shrub growth habit of *G. trifoliata* will enable comparative studies of shoot architecture, floral induction and biennial fruit bearing.

Here, we provide genomic and phenotypic resources to facilitate the use of *G. trifoliata* as a model species for comparative analyses. The phenotype of *G trifoliata* is described in terms of phenological growth stages using a Biologische, Bundesanstalt, Bundessortenamt, and Chemical Industry (BBCH) scale to assist phenotypic comparisons. A high-quality whole-genome sequence for *G. trifoliata* has been constructed with genome assembly and annotation metrics consistent with its phylogenetic position within the Rosaceae. Analysis of MADS and NAC transcription factor families reveal genome dynamics that correlate with differences in growth and reproductive phenotype between *G. trifoliata* and Rosaceae species. Lastly, a study of selection pressure upon *SEPALLATA1/2*-like *MADS8* orthologues with known intra-familial roles in fruit flesh development, demonstrates the potential for research using *G. trifoliata* to reveal new insights into fruit flesh development.

## Results

### Phenology of *Gillenia trifoliata* within a BBCH framework

To enable intra-familial comparisons, phenological stages were described within a BBCH framework. The BBCH scale provides a universal code to facilitate standardisation of key plant development phases^13^. Scales are established for model plants and key horticultural crops^14,15^.

**Fig. 1:**
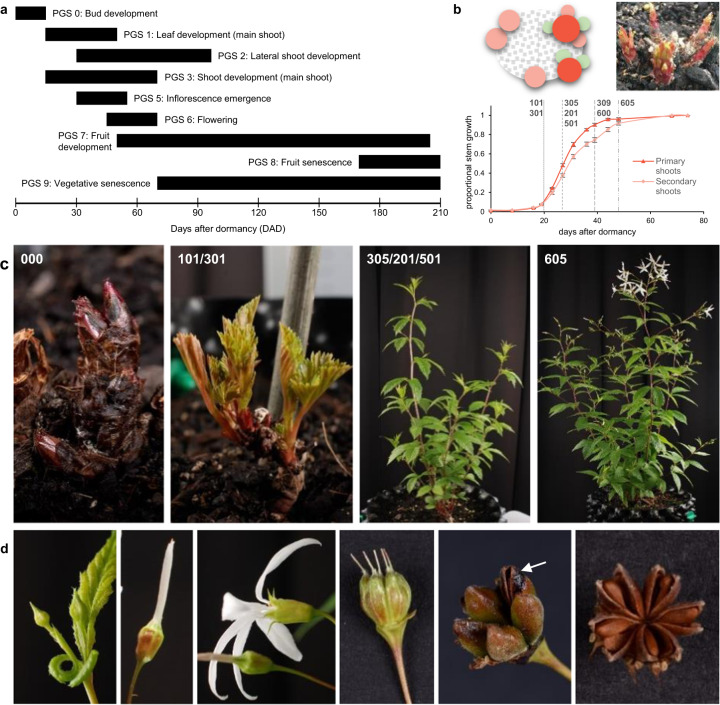
Phenological growth stages (PGS) of *Gillenia trifoliata.* **a** Duration and timing of primary growth stages over time. **b** (top left) Schematic arrangement of primary (red) and secondary (pink) reproductive and vegetative (green) shoots of a *G. trifoliata* plant during bud development (top right, PGS 0), and growth curves of primary and secondary reproductive shoots showing difference in vigour with key shoot-level growth stages indicated. **c** Key stages during whole plant development covered by the shoot-level BBCH scale with correlating growth stages given for each stage. **d** Key stages of the reproductive organ-level BBCH scale, from left, inflorescence emergence (stage 55), end of inflorescence emergence (stage 59), full flowering (stage 65), early fruit development (stage 71), early fruit senescence (stage 85), full fruit senescence (stage 89)

*G. trifoliata* is a perennial herb which grows, reproduces, and dies-back annually to a woody rhizome that then undergoes winter dormancy. The growth cycle of *G. trifoliata* can be described in nine phenological growth stages over time (Fig. 1a). Each rhizome bears multiple perennating buds that give rise to shoot systems comprised of both main and lateral growth that are mostly reproductive (Fig. 1b). Rhizome shoots differ in vigour, as primary shoots (~25% of shoots) have greater vigour than secondary shoots in terms of length, node number, axillary growth, and reproductive or vegetative fate (Supplementary Fig. 1). The difference in vigour was borne out by steeper growth in the first phase of a double-sigmoidal growth curve (Fig. 1b). To manage the growth complexity and maximise utility of a phenological tool, we describe two levels of BBCH scale: a shoot-level scale to describe whole-plant development, and an organ-level scale for reproductive development. The shoot-level scale accommodates growth complexity by being extendable for differences in vigour. Detailed description of the growth stages is provided in Supplementary Note S.1, Supplementary Figs. 1‒9, and Supplementary Tables 1 and 2.

Whole-plant development begins with main shoot growth from rhizome buds occurring over the first 30 days after dormancy (DAD, BBCH stages 0/1/3, Fig. 1c). Lateral shoot development (BBCH stage 2) and flowering (BBCH stages 5/6) occur concurrently, with flowering concentrated at the distal ends of both main and lateral shoots (Fig. 1c, d). A long period of fruit development (~110 days, BBCH stage 7) is followed by late-summer fruit senescence (BBCH stage 8). Vegetative senescence (BBCH stage 9) begins near the end of flowering and continues throughout the season.

The reproductive organ-level scale is independent of shoot position, to aid comparative sample collection. Inflorescence emergence begins (stage 51) after shoots have emerged, first individual flowers are visible (stage 55) by 30 DAD, and first flowers reach the equivalent apple “balloon” stage (stage 59) by 42 DAD. Flowers open (stage 60) with unfurling of the petal tube and proceed rapidly in 2‒3 days to fully open (stage 65), after which stamens darken, petals abscise and hypanthium remains attached. After pollination (stage 70), visible follicle growth is evident by 6 days after pollination (DAP, stage 71). Final fruit size is reached quickly at ~21 DAP (stage 72) and seed coat colour changes to burnt orange (stage 79). Fruit growth to maturity and beginning of fruit senescence (stage 80) takes ~130 DAP, similar to apple, and fruit senescence ends (stage 89) at ~160 DAP.

### Genome assembly and annotation

The genome size of *G. trifoliata* was estimated at 321.6 Mb by flow cytometry (Supplementary Fig. 10, Supplementary Table 3). The nuclear genome was firstly sequenced and assembled from 10X Chromium linked Illumina short reads, which produced two haplotype genome assemblies. One haplotype displayed improved assembly statistics, with a total size of 269 Mb, N50 of 847.9 kb and excellent BUSCO^16^ completeness (96.7% with a duplication rate as low as 2.6%). Oxford Nanopore Technology (ONT) long-read and long-range Illumina Hi-C short-read technologies were later used to improve the continuity and completeness of this haplotype assembly through post-scaffolding and anchoring scaffolds to chromosomal level, respectively (Table 1; Supplementary Fig. 11). The final assembly size was 296.3 Mb, similar to the genome size of peach^17^ (Table 2), and containing only 0.25% of gaps (N%). A total of 272.1 Mb were anchored to nine pseudo-chromosomes with lengths ranging from 22.3 to 46.3 Mb (Supplementary Table 4). Aligning 10X Illumina short reads to the assembly showed an overall alignment rate of 86.3%. BUSCO analyses^16^ predicted a high-quality assembly with completeness of 98.0% (95.6% unique and 2.4% duplicated) with 0.6% fragmented and 1.8% missing genes. K-mer analysis using 10X Illumina short reads estimated genome heterozygosity at 0.565% (Supplementary Fig. 12).

**Table 1:** Genome assembly metrics

Metric	Number	N50 (kb)	Longest (kb)	Total length (Mb)	% of estimated genome size^a^
10X contigs	7108	847.9	10,638.4	268.7	83.6
Scaffolds	1524	1800.8	17,261.3	296.3	92.1
*Pseudo-chromosomes*					
- anchored	238	2157.3	17,261.3	272.1	84.6
- unanchored	1253	–	–	24.2	7.5

^a^Estimated by flow cytometry

**Table 2:** Genome metrics comparing *Gillenia trifoliata* with selected Rosaceae species

		Amygdaloideae			Rosoideae	
Plant	*M. domestica* (GDDH13)	*P. betuleafolia* (Pbe-SD)	*G.trifoliata*	*P. persica* (v2)	*F. vesca* (v4.a2)	*R. occidentalis* (v3)
Base chromosome number (x)	17	17	9	8	7	7
Sequenced genome (Mb)	643.2	532.7	296.3	227.4	240	290
BUSCO complete genes (%)	94.9	94.8	98.0	99.0	98.1	93.9
TE(%)	57.3	46.4	47.2	29.6	31.1	56.6
GC content (%)	39.4	37.6	38.2	37.7	na	na
Number of protein-coding genes	42,140	42,520^a^	28,847	26,873	34,007	34,545
Number of transcripts	na	66,308	30,259	47,089	64,598	na
Mean gene length (bp)	na	3052	2594	3239	2953	3220
Gene density (genes/100 kb)	6.7	8.0^a^	9.7	11.8	14.2	11.9

*na* not available

^a^Number of ‘high-confidence’ genes after authors’ filtration methods applied

### Genome synteny supports ancestral predictions

**Fig. 2:**
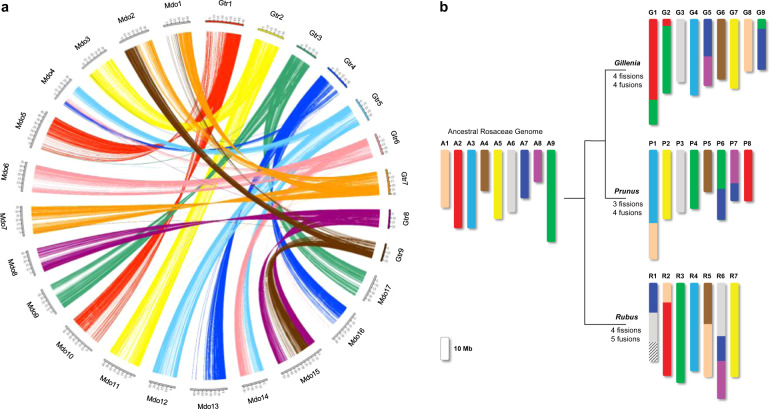
Syntenic conservation and ancestral genome model. **a** Circos plot of synteny between the genomes of *G. trifoliata* (Gtr) and apple (Mdo)^18^. Each connecting line links two homological regions longer than 10 kb. **b** Model for the evolution of present day Rosaceae species from hypothetical ancestor. The ancestral chromosomes are represented in nine colours, while chromosomes of descendants, peach^17^ and raspberry^20^, are coloured according to ancestral origin. Estimates for the number of fissions and fusions are given at each node. Dashed region of raspberry Chr01 represents area for which ancestral origin could not be determined

Comparative genome mapping was performed between *G. trifoliata* and apple^18^. Dot plot analysis using syntenic blocks with a minimum length of 5 kb sharing at least 70% nucleotide identity demonstrated a high level of conserved synteny between *G. trifoliata* and apple and pear (Fig. 2a; Supplementary Fig. 13a, b). Syntenic chromosomal regions in *G. trifoliata* typically hit two homologous regions in apple and pear^19^ as expected because of the Maleae WGD event. Seven *G. trifoliata* chromosomes were completely collinear with apple chromosomes from end-to-end: *G. trifoliata* Chr01, Chr02, Chr03, Chr05, Chr06, Chr07 and Chr08 with apple and pear Chr05/10, Chr03/11, Chr09/17, Chr12, Chr06, Chr07 and Chr08, respectively. Apple Chr13/16 have near-complete collinearity with *G. trifoliata* Chr04, except for their distal ends. Syntenic alignments of partial chromosomes replicate and validate the hypothesised reconstruction of chromosomes of the Maleae ancestor prior to WGD as described by Velasco et al.^5^. The only addition is conserved synteny between the proximal end of apple Chr04 and the distal end of *G. trifoliata* Chr04, which was not previously resolved.

Synteny was assessed against the peach^17^ and *Rubus occidentalis* (black raspberry)^20^ genomes using orthologous blocks with a minimum length of 2 and 1 kb, respectively, sharing at least 70% nucleotide identity. A high degree of synteny was detected against both genomes and, unlike the syntenic map of *G. trifoliata* versus apple, all *G. trifoliata* chromosomes hit only one orthologous region in both peach and raspberry (Supplementary Fig. 13c, d). Despite extensive rearrangements, syntenic conservation between the distal end of *G. trifoliata* Chr04 with peach Chr01 and raspberry Chr04 supports the assembly of *G. trifoliata* Chr04. Two inversions were detected within the distal halves of *G. trifoliata* Chr02 and Chr03, which were common to all four comparative species, suggesting *G. trifoliata* -only events. A single small region within the proximal half of *G. trifoliata* Chr01 had consistently low nucleotide identity across all four species, indicating putative divergence unique to *G. trifoliata*.

Ancestral genome reconstructions have been proposed for the Rosaceae family. The *G. trifoliata* genome was compared with the reconstructions of Jung et al.^10^ and showed fusion and fission events predicted for *Malus* Chr12/04/14 (*G. trifoliata* Chr05) which would have instead occurred respectively prior to, and after Maleae WGD. *G. trifoliata* therefore provides a simplified representation of the Malodae super-tribe. Combined with the conservation of synteny between *Gillenia*, peach and raspberry, a new model for the Rosaceae ancestral genome was developed. Forty-four syntenic blocks identified across all species were used to reconstruct a model of the nine chromosomes of ancestral Rosaceae (Fig. 2b, Supplementary Fig. 14). Both the new model and the model of Jung et al.^10^ required the same number of rearrangements to form raspberry (Rosoideae). However, the new model improved upon the model of Jung et al.^10^ in requiring fewer fissions and fusions to form *Prunus* (Amygdaloideae; Supplementary Fig. 15).

### Comparison of the transposable element (TE) landscape

**Fig. 3:**
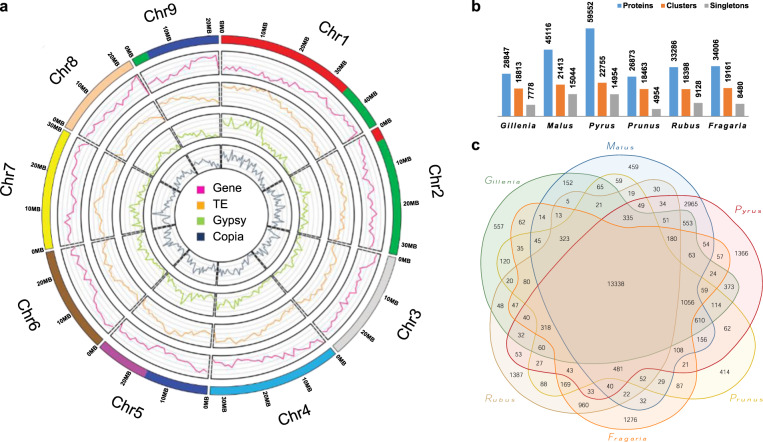
Genome features and orthologous cluster analysis. **a** Circos plot of features in *G. trifoliata* genome. Tracks from outer to inner circles indicate: chromosomes coloured by ancestral origin, gene density (each *y*-axis partition represents 100 k), the density of all annotated transposable elements (TE; each *y*-axis partition represents 200 k), the density of annotated Gypsy and Copia elements (each *y*-axis partition represents 50 k). The window size for all density tracks was 1 Mb. Orthologous cluster analysis (**b**, **c**) with a summary of orthologous clusters and singletons (**b**) and Venn-diagram of relationships between orthologous clusters (**c**) found in a comparison of annotated proteins from six Rosaceae species: *G. trifoliata*, apple^22^, pear^32^, peach^17^, strawberry^72^, and raspberry^20^

In total, 47.2% of the *G. trifoliata* genome assembly was annotated as repeat elements, mainly from long terminal repeat (LTR) and terminal inverted repeat (TIR) classes (Table 2, Supplementary Table 5). Across the nine chromosomes, Chr01 and Chr08 had the highest and lowest TE proportions respectively (Supplementary Tables 6–7). Chr01 and Chr08 were found to have respectively low and high syntenic conservation in dot plot analyses with Amygdaloideae species (Supplementary Fig. 13). The density of annotated TEs on Chr01 was highest at the proximal end and correlated with a high density of annotated Gypsy class TEs (Fig. 3a). Compared with other Rosaceae, *G. trifoliata* had proportionally higher counts of PIF_Harbinger and hAT classes, and proportionally lower counts of Gypsy and LTR_unknown classes, in both annotated and intact comparisons (Supplementary Fig. 16). Combined with the observation of a high density of annotated Gypsy classes in proximal Chr01 (Fig. 3a), degeneration of this TE type may correlate with the low syntenic conservation with apple and pear in this region.

The apple HODOR sequence (KX869746.1) of 9,716 nts was used to verify TEs detected by the Extensive de novo TE Annotator (EDTA) pipeline. An abundance of BLAST high-scoring segment pairs (HSPs) between HODOR and *G. trifoliata* TEs was found, but all TEs belonged to the LTR type. However, the aligned length of HSP was around 1 kb or less, with sequence identity ranging between 80.0% and 91.6%, which indicated that the HODOR repeat putatively appeared after speciation between *G. trifoliata* and the apple and pear ancestor.

### Comparison of the gene landscape

The genic landscape of the *G. trifoliata* genome was compared to those of other Rosaceae (Table 2). A total of 28,847 protein-coding genes were predicted in *G. trifoliata*, similar to peach, with the difference in gene density reflected in the higher TE percentage in *G. trifoliata*. The number of high confidence genes in apple and pear, compared with *G. trifoliata*, was consistent with the rapid loss of most duplicated genes in ancient polyploid plant genomes^21^. The mean gene length in *G. trifoliata* of 2594 bps was shorter than those of the Rosaceae species under comparison. A total of 514 transfer ribonucleic acids (tRNAs) were detected in *G. trifoliata*, which were most abundant on Chr08 at 2.13 tRNAs/Mb, and in notably high abundance on contig_1706_RaGOO (Supplementary Table 8).

Orthologous cluster analysis was performed with predicted proteins from *G. trifoliata*, apple (GDDH13), pear (*Pbe*-SD), peach, strawberry, and raspberry (Fig. 3b, c). Orthologous cluster analysis was previously shown to be a useful metric of annotation quality^19^. The number of proteins, clusters, and singletons and distribution of clusters for the *G. trifoliata* protein set was comparable to those of Rosaceae species of interest, relative to its phylogenetic position. Gene Ontology (GO) enrichment was assessed for clusters of *G. trifoliata* only and all-but-*G. trifoliata* clusters, and no GO terms were found to be enriched (Supplementary Table 9).

### Genome dynamics related to growth habit

Changes to gene family size provide important sources of genetic variation for evolution^21^. Gene family sizes between *G. trifoliata* and apple were assessed to identify genome dynamics correlating with differences in phenotype. The MADS transcription factor family is known to control many aspects of growth and development and was mined from *G. trifoliata* and two recent apple genomes^18,22^. The *G. trifoliata* MADS family was similar in size to that of peach (Table 3). Between subfamilies, Type II MADS had a bias toward retention of both homoeologous gene pairs, whereas the Type I MADS subfamily was similar in size between apple, *G. trifoliata* and peach (Table 3; Supplementary Fig. 17), demonstrating contrasting evolutionary dynamics observed previously^23^.

**Table 3:** Comparison of gene family size based on annotated proteins lists from *Arabidopsis*, *Prunus*, *Malus* and *Gillenia*

Gene family	*Arabidopsis*^a^	*Prunus*^b^	*Gillenia*	*Malus*
*Transcription factors*				
MADS	100	82	97	140
- Type I (α/β/γ)	23/18/13	21/7/14	27/10/18	22/10/22
- Type II (C/*)	39/7	34/6	36/6	77/9
NAC	103	115	110	196
TALE	22	20	22	41
- BEL	13	10	11	19
- KNOX	9	10	11	22
*Cell wall loosening*				
Expansins	35	na	31	54

*na* not available

^a^arabidopsis.org

^b^planttfdb.gao-lab.org

**Fig. 4:**
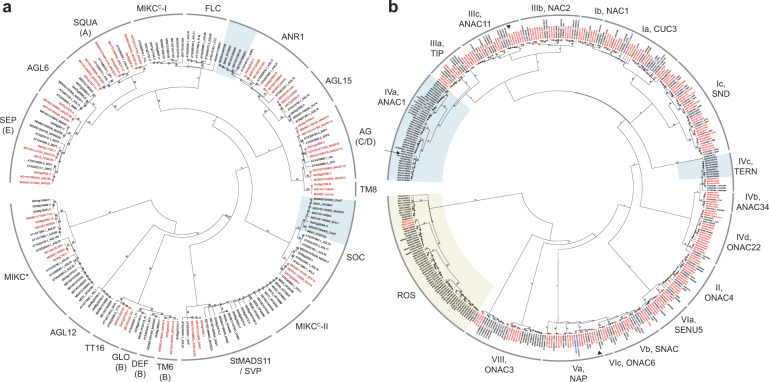
Phylogenetic trees of Type II MADS and NAC transcription factor. Phylogenetic trees of (**a**) Type II MADS and (**b**) NAC transcription factors from apple (*Md*), *Gillenia*, and *Arabidopsis*. Ratios of two homoeologous apple and one orthologous *Gillenia* genes highlighted in red font. Clade expansions of interest shaded green, putative Rosaceae-specific NAC clade shaded yellow. Maximum likelihood bootstrap values from 100 datasets are shown at branches. Prefixes: Gtr, *Gillenia trifoliata*; MDXXG, apple GDDH13^22^; MDXX_HF, apple HFTH1^18^; AT, *Arabidopsis thaliana*; Soly, tomato. MADS subfamily names follow standard convention and^27^ for Maleae-specific MIKC^C^-I and –II, letters in parentheses denote floral homeotic classes. NAC subfamily names follow^81,82^. Apple fruit development NACs in ANAC1 tandem array (arrow) and fruit ripening clades (arrowhead) identified in ref. ^35^

Two notable clade contractions in *G. trifoliata* were observed for Type II MADS, which may correlate with differences in growth habit (Fig. 4a). In the *ANR1* clade, two homoeologous loci were observed in apple, whereas the orthologous locus was absent in *G. trifoliata*. In *Arabidopsis*, *ANR1* controls lateral and primary root development^24^. Comparison of the loci at the genomic level identified a large inversion in *G. trifoliata* bordering the site where the *ANR1* locus should be, suggesting disruption of this locus during genomic rearrangements (Supplementary Fig. 18a). Investigation of the *ANR1* clade across the Rosaceae found an absence of *ANR1*-like sequences in strawberry and raspberry annotations, whereas multiple annotations were observed in pear and single annotations were present in peach and rose (Supplementary Fig. 18b), suggesting a correlation between *ANR1* abundance and growth habit, given *Rosa* contains both shrub and tree habits. In apple, *MdSOC1*-*like* was linked to a QTL on Chr01 for biennial bearing^25^ and resides on one of two homoeologous loci (Chr01/07) that each contain three *SOC1*-like genes in tandem. In annual-bearing *G. trifoliata*, only one complete and two pseudogenes were detected at the orthologous locus.

A notable asymmetric clade contraction in apple was observed in the *StMADS11/SVP* clade, where five genes exist on *G. trifoliata* and apple Chr08, but only two on apple Chr15, demonstrating a departure from retention of duplicated genes post-WGD strongly observed in this gene subfamily. In apple, this clade is associated with the regulation of dormancy^26^. Like apple, *G. trifoliata* has a strong dormancy requirement for optimum growth (Supplementary Note 1.2), which correlates with the near-equivalent number of dormancy-associated MADS in apple. In addition, two clades putatively apple- and pear-specific, MIKC^*C*^-I and -II, showed different evolutionarily dynamics with MIKC^*C*^-II missing entirely from *G. trifoliata*. In apple, the expression of MIKC^*C*^-II genes was similar to that of *SVP*-genes with known involvement in dormancy regulation^27^.

### Genome dynamics related to fruit development

*G. trifoliata* bears a dry follicetum (aggregate of follicles), which develops from an ovary separated from the hypanthium. Key differences from apple include ovary and hypanthium fusion, development of fleshy tissues, and ripening. Gene family analysis was centred on TALE and NAC, and MADS transcription factors, which have roles in organ boundary and floral development, respectively, and expansins with roles in ripening.

Following WGD, transcription factors are frequently retained, to maintain protein stoichiometry or gene dosage balance^21^. A 2:1 apple:*Gillenia* ratio dominated the TALE family (Supplementary Fig. 19), and conspicuous amongst this was a 1:1 ratio within a clade related to *BELLRINGER* (*BLR*). In *Arabidopsis*, *BLR* controls organ boundary, floral and fruit patterning, inflorescence stem development, and is a ubiquitous hub protein in floral gene regulatory networks^28^. In apple, *BLR*-like MD07G1205600 (Supplementary Table 10 for MDP to GDDH13/HFTH1 conversions) is highly expressed in floral buds treated with cytokinin to induce floral transition^29^ and is also expressed in stem, floral and fruit tissues^30^. The 1:1 ratio in the *BLR*-like clade was found to be conserved in domesticated and crab apple^18,22,31^ and pear^19,32^ genomes, suggesting importance for pome-fruiting species. A 1:1 ratio was also observed in the NAC *CUC3* clade (Fig. 4b), which sits within a subfamily dominated by 2:1 ratios; however, this was conserved only in domesticated apple genomes^18,22^. *CUC3* controls organ boundary development and functions upstream of *BLR*^33^.

In *G. trifoliata*, 110 NACs were identified, similar to 112 in strawberry^34^ and 115 in peach (Table 3). In apple, 196 unique NACs were detected using two recent genome annotations^18,22^ (Supplementary Table 11 for MDP to GDDH13/HFTH1 conversions). With few exceptions, a 2:1 apple:*Gillenia* ratio dominated, including in clades with known roles in organ boundary development and ripening (e.g. Ia/CUC3, Va/NAP, Vb/SNAC; Fig. 4b). Notable exceptions included a clade putatively Rosaceae-specific (Fig. 4b ‘ROS’), which may be subject to evolutionary pressures to maintain a constant overall family size, similar to Type I MADS, as 31 NACs were identified in *Gillenia* and strawberry, and 33 in apple despite WGD (Supplementary Fig. 20a). Also notable was a clade expansion related to a tandem gene array in subfamily IVa/ANAC1. In the array *Gillenia* has six NACs on Chr07, while apple has 20 and nine NACs respectively on syntenic Chr01 and Chr07. In strawberry this locus has 10 NACs (Supplementary Fig. 20b) suggesting loss of NACs in *G. trifoliata* and duplication of the entire 10-NAC array on apple Chr01. MD07G1162700 is situated within the Chr07 tandem array (Fig. 4b, arrow) and was recently associated with ethylene/auxin cross-talk for apple fruit development and ripening^35^. In strawberry, all 10 NACS are expressed in fruit, but consistent with its non-climacteric physiology, were mostly down-regulated in red versus green fruit^34^. Fewer tandemly arrayed NACs in *G. trifoliata* may correlate with its dry-fruit physiology. Tandem gene arrays were found to be frequently collapsed in a comparison of the *Rubus* v1 and v3 genome assemblies^20^, therefore the assembly of *G. trifoliata* loci for SOC, TERN, and ANAC1 clades was checked against raw ONT reads. SOC and TERN loci showed >95% identity between assembly and raw ONT reads, whereas the ANAC1 locus showed some evidence for possible collapse. However, error-correction and reannotation of the ANAC1-associated ONT contig, revealed only one additional NAC as a result of the alternate genome arrangements (Supplementary Fig. 21).

In the expansin gene family, clades found to be associated with ripening in apple and pear^36^ (Supplementary Fig. 22) were found to have a dominant 2:1 apple:*Gillenia* ratio, suggesting that, despite their role in ripening, selective pressures have not resulted in isolated clade expansions outside WGD. Expansins were of note as a difficult family for gene annotation programs to detect^37^; however, nearly all computer-generated annotations for *G. trifoliata* were full models.

Lastly, floral homeotic Type II MADS, *SQUA* (A), *AG* (C), and *SEP* (E), maintained a 2:1 apple:*Gillenia* ratio (Fig. 4a). Putative stoichiometric preservation in the A/C/E floral homeotic clades is consistent with the floral quartet model of ternary complex formation for molecular function^38^. The predominant 2:1 ratio within these clades suggested potential for different selective pressures on duplicated genes, which was further explored with E-type *MADS8* as the exemplar.

### *G. trifoliata* as a negative control for fruit flesh development

**Fig. 5:**
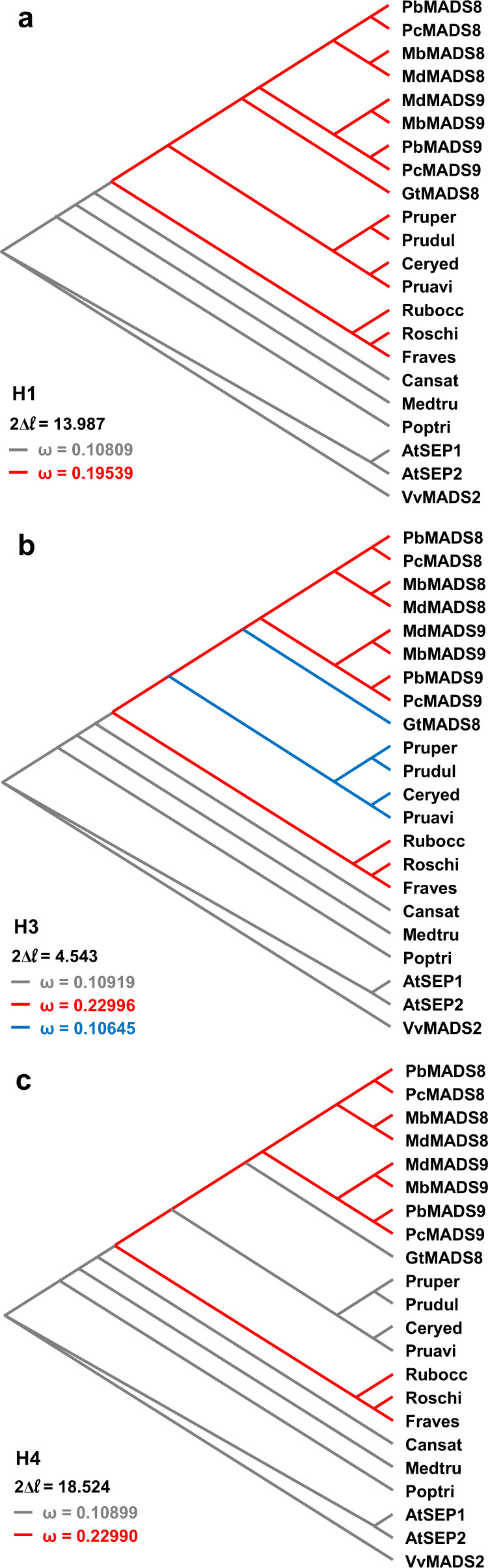
Cladograms of Rosid *SEP1/2*-like orthologues depicting hypotheses for variable branch-wise d*N*/d*S* (ω) ratios. **a** Two-ratio hypothesis: Rosaceae versus non-Rosaceae orthologues. **b** Three-ratio hypothesis: accessory-dependent fruiting Rosaceae versus non-accessory-dependent Rosaceae versus non-Rosaceae orthologues. **c** Two-ratio hypothesis: accessory-dependent fruiting Rosaceae versus non-accessory-dependent fruiting Rosaceae and non-Rosaceae orthologues. Prefixes: pear (Pb, *Pyrus betuleafolia*; Pc, *P. communis* BartlettDH); apple (Mb, *Malus baccata*; Md, *M. domestica* GDDH13); Gt, *Gillenia trifoliata*; Pruper, *Prunus persica* (peach); Prudul, *P. dulcis*; Ceryed, *Cerasus yeodonis*; Pruavi, *P. avium*; Rubocc, *Rubus occidentalis* (raspberry); Roschi, *Rosa chinensis*; Fraves, *Fragaria vesca* (strawberry); Cansat, *Cannabis sativa*; Medtru, *Medicago truncatula*; Poptri, *Populus trichocarpa*; At, *Arabidopsis thaliana*; Vv, *Vitis vinifera*. 2Δl, Likelihood ratio test against Χ^2^(df = 1, a = 0.05) = 3.841

Many successful Rosaceous fruit crops rely on the development of accessory tissues (receptacle and hypanthium) as edible or structural components. In addition, the presence of a floral hypanthium is a unifying character for all species within Rosaceae^2^. In apple and strawberry, genes related to *Arabidopsis SEPALLATA1/2*, *MdMADS8/9* and *FaMADS9*, were implicated in the growth and development of fruit flesh^39,40^. Owing to intrafamilial functional similarity and importance for reproductive development, *SEP1/2*-like orthologues were assessed for selection pressure via the ratio of nonsynonymous/synonymous substitution rates (*d*_*N*_*/d*_*S*_, *ω*). A series of branch-wise hypotheses were tested using a phylogeny of Rosid *SEP1/2*-like genes (Fig. 5, Supplementary Table 12a). It was found that Rosaceae *SEP1/2*-like genes had a highly significant (*p* < 0.005) near twofold increase towards positive selection compared with non-Rosaceae orthologues (H1; Fig. 5a). A 3-ratio test suggested uneven selection pressure across Amygdaloideae subfamily (H2; Supplementary Fig. 23a). Therefore, a hypothesis was tested in which Rosaceae species with accessory-dependent fruit (Maleae and Rosoideae) were under different selective pressures from those without (*Gillenia*, *Prunus* and *Cerasus*). This hypothesis showed a clear twofold increase towards positive selection of *SEP1/2*-like genes in accessory-dependent fruiting Rosaceae and was statistically significant in both 3-ratio (H3; Fig. 5b) and 2-ratio (H4; Fig. 5c) hypotheses. Hypotheses testing for different selection pressures between Maleae homoeologues (*MADS8 vs. MADS9*), were not statistically significant (H5 & H6; Supplementary Fig. 23a). A second study was conducted for *SEP1/2*-like orthologues without *Gillenia*, and while the 2-ratio hypothesis (H2) was found to be statistically significant, the 3-ratio hypothesis was not (H3; Supplementary Table 12b; Supplementary Fig. 23b), suggesting *Gillenia* provided greater statistical power along with the ‘negative control’, fleshless non-accessory-dependent phenotype. Lastly, a third study was conducted for *SEP3*-like Rosid orthologues. *Arabidopsis SEP3* is a central hub protein in floral gene networks^41^ but has no documented relationship with accessory flesh development. Here, the increase towards positive selection was reduced, both 2-ratio hypotheses (H1 & H3) were less significant compared to corresponding *SEP1/2-like* hypotheses (Fig. 5a, c), and the 3-ratio hypothesis (H2) was not statistically significant (Supplemental Table 12c; Supplementary Fig. 23c).

## Discussion

The combination of phenotypic and genomic resources provided here will facilitate the use of *G. trifoliata* as a model species and ‘rosetta stone’ for translational science. The phenotype of *G. trifoliata* has been described in detail in a BBCH framework, to enhance comparative studies within Rosaceae, and to characterise the development of characters of interest, such as annual bearing, cane-like habit, and dry fruit development. The dry fruit phenotype provides a new tool, previously unavailable for apple and pear, to study fleshy fruit development in these two economically important fruit crops. The *G. trifoliata* genome provides new perspectives on genome dynamics relating to growth and reproductive development within Rosaceae. The absence of *ANR1* genes in *G. trifoliata*, strawberry, and raspberry correlates with a herb/shrub growth habit and specifically in *G. trifoliata* may correlate with its reversion to a rhizomatous herb habit after separation from the Maleae ancestor^42^. Many clade contractions were observed in *G. trifoliata* compared with apple, particularly around flowering- and dormancy-related MADS genes, which may correlate with its annual bearing habit. A tandem NAC gene array, which houses a ripening-associated NAC in apple^35^, has expanded greatly in fleshy-fruiting apple, but appears to have contracted in dry-fruiting *Gillenia* relative to fleshy-fruiting strawberry, and provides additional support for further study of NAC-regulated fleshy fruit ripening. Overall, gene family analysis revealed a strong retention of a 2:1 apple:*Gillenia* ratio, which demonstrates preferential retention of homoeologues in these families rather than selective pressure in favour of segmental duplication and clade expansion. In contrast to this was the observation of a Maleae-conserved 1:1 ratio in an apple homologue of *BLR*, an important floral gene network hub gene with roles in regulating organ boundaries^28^ and that provides a candidate to begin unravelling the mechanism behind ovary-hypanthium fusion, which has been pivotal in the development of the pome-fruit structure. The case study of selective pressures acting upon *MADS8*-like genes in Rosids using the ratio of nonsynonymous to synonymous substitutions demonstrated the power of using *Gillenia* as a negative control for fleshy-accessory fruit development and exemplifies its potential utility for future studies of fleshy fruit evolution.

## Methods

### Plant materials

Mature plants of *G. trifoliata* were obtained from Wake Robin Nursery, Balclutha, New Zealand (https://www.wakerobin.co.nz/). During the growing season, plants were held in glasshouse conditions at Plant & Food Research, Auckland, New Zealand with automated watering and automated shading at temperatures above 28 °C. During winter, plants were held in a cold room for twelve weeks at 5 °C to simulate overwintering. Flowers were outcrossed and hand-pollinated to induce fruit set. Phenotypic assessments were performed across three multi-stemmed individuals.

### Molecular extraction, library preparation and sequencing

High molecular weight (HMW) nuclear genomic DNA (ngDNA) was extracted from isolated nuclei prepared as described in^43^ with the following modifications: 1.5 g mature leaves (for 10X Chromium) or 2 g young leaves (for Oxford Nanopore) were fine ground in liquid nitrogen with a mortar and pestle, suspended in 300 mL nuclei isolation buffer and filtered through two layers of Miracloth. All centrifugation steps were performed at 1800 × *g* at 10 °C. HMW ngDNA was extracted from isolated nuclei using the CTAB/NaCl/Proteinase K method described in ref. ^44^ with the following modifications: nuclei pellet was re-suspended in 15 mL CTAB buffer supplemented with 2 mg Proteinase K, and after lysis incubation was extracted with an equal volume of 24:1 chloroform:isoamyl alcohol. QC was performed with Qubit dsDNA high sensitivity assay (Life Technologies), Nanodrop spectrophotometer (Thermo Fisher Scientific) and pulsed-field gel electrophoresis. HMW ngDNA was supplied to Novogene (Hong Kong) for Illumina 10X Chromium sequencing which generated short-read data of 109X coverage, and to the Australian Genome Research Facility (AGRF; Melbourne, Australia) for long-read Oxford Nanopore Technologies (ONT) sequencing which generated data of 123X coverage.

For long-range Hi-C sequencing, chromatin was extracted from isolated nuclei purified using polyvinylpolypyrrolidone and Percoll gradients as described in ref. ^45^. Hi-C libraries were prepared according to ref. ^46^ with modifications. Nuclei were crosslinked with 4% formaldehyde, quenched, washed, lysed and chromatin normalised according to Dovetail™ Hi-C kit (Dovetail Genomics). Chromatin fragmentation and biotinylation were performed with Fragmentation buffer and enzyme mix from Phase Genomics Hi-C kit for Plants v1 (Phase Genomics). Ligation was achieved with T4 DNA ligase and buffer (Invitrogen), at 16 °C overnight with rotation. Crosslink reversal was performed with 0.4 μg/μL Proteinase K (Qiagen) in 1X CutSmart buffer (New England Biolabs) and DNA purified with 2X AMPure XP Beads. The short insert library was prepared with NEBNext Ultra II FS DNA library prep kit for Illumina (New England Biolabs). DNA was fragmented for 8 min in dsDNA fragmentase enzyme mix, ligated with Illumina adaptor, digested with USER enzyme mix and purified with AMPure XP beads. Biotinylated molecules were captured with Dynabeads M280 (Invitrogen) according to the manufacturer’s protocol. The captured library was amplified with NEB Q5U DNA polymerase, the universal Illumina primer and an index primer 12 with the following PCR profile: 98 °C, 30”-(98 °C, 10”−62 °C, 30”−65 °C, 60”)x14-65 °C, 5 min- stop. The amplified library was size selected with AMPure XP beads at 0.6X/0.2X). The average fragment size of the selected amplicons was 399 bp estimated by capillary electrophoresis (Fragment Analyzer). The amplicons were sequenced by AGRF (Australia).

Short-read RNA-Seq libraries were prepared for three floral stages following^47^ and sequenced by AGRF (Australia) on HiSeq platform (Illumina). Long-read RNA libraries were prepared for shoot, root and leaf tissues using the Direct cDNA Sequencing kit SQK-DCS109 (Oxford Nanopore) and sequenced with Oxford Nanopore minION device using Flowcell FLO-MIN106.

### Genome assembly

The genome size of *G. trifoliata* was estimated using flow cytometry as described in ref. ^48^. Leaves from *Malus domestica* ‘Royal Gala’ or *G. trifoliata* were co-chopped with each of two internal reference standards, *Trifolium repens* or *Bellis perennis*. Resulting ratios were used to deduce the estimated genome size for *Gillenia* using the estimated genome size for apple from the latest HFTH1 genome^18^ of 708.54 Mbp.

Sequencing data quality was assessed using FastQC (v0.11.7)^49^ and PycoQC (v2.5.0.21)^50^ for 10X Chromium and ONT reads, respectively. Hi-C data quality was assessed using BWA (v0.7.17)^51^ following the PhaseGenomics read mapping strategy (https://phasegenomics.github.io/2019/09 /19/hic-alignment-and-qc.html) and the script “hic_qc.py” (https://github.com/phasegenomics/hic_qc). K-mers (*k* = 21) were generated from 10X Chromium reads using Jellyfish (v2.2.10)^52^. The k-mer count histogram and the genome heterozygosity were analysed and visualised with the online version of GenomeScope2 (http://qb.cshl.edu/genomescope/genomescope2.0/)^53^. The initial *de novo* haplotype assemblies were generated from 10X Chromium short reads with effective coverage of 45X using Supernova (v1.2.2)^54^ (‘–maxreads=130716163’). The haplotype assembly with longer N50 and higher BUSCO score was selected, contaminating sequences were removed with Kraken2^55^ against RefSeq93 database^56^, and allelic contigs were identified and reassigned using Purge Haplotigs^57^. The later-obtained ONT reads with 123X coverage were assembled using FLYE (v2.5)^58^ and subsequent contigs were used for post-scaffolding the cleaned 10X Chromium-based assembly using RaGOO^59^ followed by gap-filling with ONT reads using TGS-GapCloser (v1.1.1)^60^. Hi-C data were used to construct chromosomal-level assembly utilising HiC-Suite (https://github.com/pfrnz/HiC-Suite), to construct joins based on visualising Hi-C contact maps. The resultant assembly was then compared and optimised with Hi-C scaffolding results produced from ALLHIC (v0.9.8)^61^ to additionally join a few short scaffolds. Results from a syntenic map with the *Malus domestica* genome^18^ generated from MUMmer (v4.0.0)^62^ were used to further inform some Hi-C interactions missed in previous steps. Final pseudo-chromosomes were determined with a whole-genome Hi-C contact map. Duplicated un-anchored sequences were removed using ‘gt sequniq’ in GenomeTools^63^. Genome assembly completeness was assessed by mapping 10X Chromium Illumina short reads to the assembly using bowtie2 (2.3.4.3)^64^, and with BUSCO analyses (v3.0.2)^16^ against “embryophyta_odb10” database (updated on 2019-11-21).

### Genome annotation

TEs were detected with EDTA pipeline (v1.9.3)^65^ using parameters “–species others –step all –sensitive 1 –anno 1 –evaluate 1 –threads 20”. The *Gillenia* genome was further soft-masked with RepeatMasker-open-4-0-5 (-pa 20 -e ncbi -gff -xsmall –poly). Illumina short-read RNA-seq data from three flower stages were mapped to the soft-masked genome using STAR (v2.6.1d)^66^ (–readFilesCommand zcat –outSAMattrRGline ID:$sample –runThreadN $thread –outBAMsortingThreadN $thread –outSAMtype BAM SortedByCoordinate). ONT long-read RNA-seq data were called with guppy/4.2.2. Adapters were removed and chimeric reads were split with porechop/0.2.3, then further filtered with quality (Q7) and minimum read length of 300 using filtlong v0.2. The cleaned reads were mapped to the *Gillenia* genome with minimap2/2.17 -ax splice. Alignments were compressed and indexed with samtools v1.10. Quality checks on ONT sequencing data, long RNA reads, and mapping were carried out with MinIONQC.R, FastQC v0.11.7, and qualimap v2.2.1, respectively. Taxonomy classification of the cleaned ONT RNA-seq reads was performed with Kraken2^55^ against RefSeq93 database^56^, and visualised with Krona (v2.7). The ONT long-read and Illumina short-read RNA-seq alignments were used as hints in BRAKER (v2.1.0)^67^ to inform gene prediction with parameters “–species = Gtr.NCBI.softmasking –gff3 –cores = $thread –workingdir = $outDir –augustus_args = ‘–strand = both –genemodel = complete –alternatives-from-evidence = true –noInFrameStop = true’ –overwrite”. Transfer RNA was detected with tRNAscan-SE (v2.0)^68^.

### Genome synteny and ancestral reconstruction

Dot plots were generated using several commands from MUMmer (v4.0.0 beta2)^62^. Syntenic blocks were produced using “nucmer –mum”, then “delta-filter” with appropriate nucleotide identity (-i) and syntenic block length (-l) settings for every comparison, and finally “mummerplot” with the given options of “–large –png -R -Q –filter –layout -p”. Dots presented for *Gillenia* versus *Malus*^18^ and *Pyrus*^19^ chromosomes have 70% nucleotide identity with a minimum length cutoff of 5 kb. Dots presented for *Gillenia* versus *Prunus*^17^ and *Rubus*^20^ have 70% nucleotide identity with a minimum length cutoff of 2 and 1 kb respectively. To construct the ancestral genome, forty-four syntenic blocks were identified across the dot plots between *Gillenia* versus *Malus*, *Prunus*, and *Rubus*. Blocks were numbered and owing to high syntenic conservation were able to be manually assembled to elucidate most parsimonious genomic rearrangements. Ancestral blocks in descendent genomes were drawn in MapChart^69^ and ancestral chromosome sizes were estimated based on size of descendant blocks.

The circos plot of genomic synteny between apple and *G. trifoliata* was generated using Circos visualisation tool (v0.69-6)^70^. The homological regions were produced in advance using nucmer (–mum) and dnadiff with default options from MUMmer^62^. Multiple-to-multiple coordinates file “mcoords” filtered out regions shorter than 100 bp length and then reformatted and inputted in the command “bundlelinks” (-max_gap 10000 -min_bundle_size 10000 -min_bundle_membership 3) from circos tools (v0.22) within Circos package to build connecting lines. The final plot was generated using “circos” command with customised config file from Circos.

### Comparative transposable element identification

Genome assemblies were collected from rosaceae.org, including *Malus domestica* (GDDH13 and HFTH1), *Pyrus communis* (BartlettDHv2), *P. betulaefolia* (PbeSD), *P. persica* v2.0, *Rubus occidentalis* v2.1, and *F. vesca* v4.0.a1. EDTA (v1.9.3)^65^, a filtering package based on multiple repeats detection tools such as LTR_Finder, LTRharvest, HelitronScanner, LTR_retriever, TIR-Learner and RepeatModeler, was run in the conda environment in a consistent way on the *Gillenia* genome and downloaded genome assemblies. The cross-species summary was drawn based on the result in EDTA.intact.gff3 and EDTA.TEanno.gff3 files for each genome.

### Orthologous gene clustering

OrthoVenn2^71^ was used to identify orthologous gene clusters between *Gillenia* and selected species of Rosaceae. Predicted protein lists were obtained from Genome Database for Rosaceae (rosaceae.org) for *F. vesca* v4.0.a2^72^, *R. occidentalis* v3.0^20^, *P. persica* v2.0.a1^17^, *M. domestica* GDDH13 v1.1^22^, and *P. betulaefolia* v1.0^32^.

### Gene family identification and phylogenetic analysis

Protein sequences identified using the first apple genome^5^ and *Arabidopsis* were used to perform all-to-all BLASTp searches (e-value 1e−50) against predicted protein models from two recent apple genomes, *GDDH13* and *HFTH1*^18,22^ and the *Gillenia* genome. GDDH13 gene models were used in preference to HFTH1 models as the base set because there was a higher incidence of concatenated gene models in the HFTH1 predictions. Reciprocal BLASTn was used to link apple models from the same locus in the two apple genomes and to identify unique models present only in one genome. Complete lists of *Gillenia* and *Malus* models were then used to perform BLASTp searches (e-value 1e−05) of each other’s genomes. To validate familial relationships, all models were subject to MEME analysis^73^ to confirm the presence of familial motifs and were also checked against the NCBI Conserved Domains Database (CDD). Protein sequences were aligned in Geneious 10.2.6 (Biomatters Ltd, Auckland, NZ) using ClustalW and BLOSUM cost matrix. For each alignment, the region of significant alignment was isolated and used to generate a phylogenetic tree with PhyML^74^ using the JTT substitution model and bootstrap analysis of 100 data-sets. For clade-specific analyses of additional Rosaceae species, the following annotations were used: *P. communis* BartlettDH^19^, *P. betulaefolia*^32^, *P. persica* v2.0.a1^17^, *F. vesca* v4.0.a2^72^, *R. occidentalis* v3.0^20^, and *Rosa chinensis* ‘Old Blush’ v2^75^.

### dN/dS analysis

Orthologues to *Gillenia SEP1/2*-like Gtr03.g9252.t1 and *SEP3*-like Gtr04.g13187.t2 were identified by reciprocal best BLAST hits. Rosaceae species, *M. domestica* GDDH13^22^, *M. baccata* v1^31^, *P. communis* BartlettDH^19^, *P. betulaefolia*^32^, *P. persica* v2.0.a1^17^, *P. dulcis* v1^76^, *P. avium* v1^77^, *Cerasus* x *yedoensis* v1^78^, *F. vesca* v4.0.a2^72^, *R. occidentalis* v3.0^20^, and *R. chinensis* ‘Old Blush’ v2^75^ were found using Genome Database for Rosaceae (https://www.rosaceae.org/) BLAST tools. Non-Rosaceae species, *Cannabis sativa*, *Medicago truncatula*, *Populus trichocarpa*, and *Vitis vinifera* were found using NCBI (https://www.ncbi.nlm.nih.gov/) BLAST tools, and *Arabidopsis thaliana* was found using TAIR (arabidopsis.org) BLAST tools. For Rosaceae species orthologues were also confirmed by synteny where possible. Protein and nucleotide alignments were performed with Clustal Omega (v 1.2.4)^79^ using default parameters and nucleotide alignments were manually corrected to ensure codon-alignment. Branch-wise dN/dS ratios were calculated with codeml in PAML v4.9i^80^.

## Supplementary information

Supplemental material

## Data Availability

The data have been deposited at DDBJ/ENA/GenBank under the accession JAEHOF000000000, BioProject PRJNA669900, BioSample SAMN16480607. The *Gillenia* genome is available in the Genomic Database for Rosaceae (GDR): www.rosaceae.org.
