# Supplementary material for: The Gillenia trifoliata genome reveals dynamics correlated with growth and reproduction in Rosaceae
Source: Hortic Res. 2021 Nov 1;8:233. doi: 10.1038/s41438-021-00662-4 (PMC8558331; doi:10.1038/s41438-021-00662-4)
Supplement: Supplementary file 1 — Supplemental material [file 41438_2021_662_MOESM1_ESM.pdf]

Supplementary Information

Contents

Supplementary Notes 3

S.1 Phenological growth stages for *Gillenia trifoliata* 3

S.1.1 Considerations for *Gillenia trifoliata* 3

S.1.2 Whole plant phenology 4

S.1.3 Shoot-level BBCH scale 5

S.1.4 Reproductive Organ-level BBCH scale 9

S.1.5 Correlations between growth stages 11

References 12

Supplementary Figures 13

Supplementary Figure 1. Aerial shoot system development 13

Supplementary Figure 2. Principle growth stage 0: bud development 14

Supplementary Figure 3. Principle growth stages 1-3: leaf, lateral, and main shoot development 15

Supplementary Figure 4. Principle growth stage 5: inflorescence emergence 16

Supplementary Figure 5. Principle growth stage 6: flowering (main and lateral shoots) 17

Supplementary Figure 6. Organ-level growth stages for inflorescence emergence and flowering 18

Supplementary Figure 7. Organ-level growth stages for fruit development 19

Supplementary Figure 8. Fruit development growth curves 20

Supplementary Figure 9. Organ-level growth stages for ripening 21

Supplementary Figure 10. Flow Cytometry Histograms 22

Supplementary Figure 11. K-mer distribution for estimation of genome size. 23

Supplementary Figure 12. Hi-C Contact Map 24

Supplementary Figure 13. Syntenic dot plots between *Gillenia* and Rosaceae species. 25

Supplementary Figure 14. Ancestral blocks detected from syntenic dot plots between *Gillenia* and Rosaceae species. 26

Supplementary Figure 15. Genome rearrangements predicted for present day Rosaceae species from common ancestor 27

Supplementary Figure 16. Proportional counts of TEs in *Gillenia* and Rosaceae species. 28

Supplementary Figure 17. Phylogenetic tree of Type I MADS transcription factors. 29

Supplementary Figure 18. *ANR1* locus 30

Supplementary Figure 19. Phylogenetic trees for TALE transcription factors 31

Supplementary Figure 20. Phylogenetic trees for NAC transcription factor clades of interest 32

Supplementary Figure 21. *Gillenia ANAC1* locus 33

Supplementary Figure 22. Phylogenetic tree of expansins 34

Supplementary Figure 23. Cladograms of branch-wise hypotheses testing variable dN/dS ratios 36

Supplementary Tables 37

Supplementary Table 1. Shoot and reproductive organ level BBCH scales for *Gillenia trifoliata* 37

Supplementary Table 2. Summary of correlative growth stages for *Gillenia trifoliata* 39

Supplementary Table 3: Flow cytometry DAPI peak data 40

Supplementary Table 4: Pseudo-chromosome assembly metrics for *Gillenia trifoliata* 41

Supplementary Table 5: Coverage of transposable elements by class for *Gillenia* genome 42

Supplementary Table 6: Coverage of transposable elements by chromosome for *Gillenia* genome 43

Supplementary Table 7: Annotated transposable elements by class per *Gillenia* chromosome 44

Supplementary Table 8: Transfer RNA by chromosome and unanchored contigs for *Gillenia* 45

Supplementary Table 9: GO enrichment for orthologous clusters of interest between *Gillenia*, *Malus*, *Pyrus*, *Prunus*, *Fragaria* and *Rubus*. 46

Supplementary Table 10: Conversion table of TALE transcription factor MDP models 47

Supplementary Table 11: Conversion table of NAC transcription factor MDP models 48

Supplementary Table 12. Branch-wise hypotheses testing dN/dS ratios 49

Supplementary Notes

S.1 Phenological growth stages for *Gillenia trifoliata*

To establish a framework for comparative analyses we describe the whole plant phenotype of *Gillenia trifoliata* using a Biologische Bundesanstalt, Bundessortenamt, and Chemical Industry (BBCH) scale. BBCH scales provide a universal code with standardised descriptions of plant development in a format compatible with electronic data handling. The general organisation of the scale is reviewed in [1]. Briefly, the scale is organised into ten principle growth phases, numbered 0-9, which represent the major stages of the plant life cycle. The ten principle growth stages are not strictly sequential, some may occur in parallel, and some may not occur at all. Within each principle growth stages are secondary growth stages used to precisely define key points in time or development. Secondary growth stages are numbered 0-9 and when combined with principle growth stages form a two-digit code e.g. inflorescence emergence primary growth stage 5 begins and ends with stages 50 and 59 respectively.

Typically the principle and secondary growth stages are enough to encompass the significant events of most plants, however, in some cases further subdivision is required; mesostages were added to enable this. Mesostages are numbered 0-9 and occur between principle and secondary growth stages. They are chiefly used to separate events on the main stem from events on subsequent stem orders or branches. For instance, in the two-digit system, stage 65 represents 50% flowers open on the main shoot, in a three-digit system this becomes 605 and the same event on a side shoot of the 5th order is stage 655.

Often, BBCH codes are assigned descriptors which cannot be identified in real-time, for instance, mid-point secondary growth stage codes are often described as a part of the total character. In such cases, the finality of the character must be known in advance in order to identify earlier stages. To increase the utility of the BBCH scale, alternative definitions based on correlations with coincident growth stages can help. For example, in *Arabidopsis*, the mid-way code for flowering (stage 65) is coincident with a timed decrease in the rate of stem elongation, thus allowing real-time identification for standardised data collection [2].

A consideration for the BBCH scale for *G. trifoliata* is that *Gillenia* is not a crop plant, but aspects of its growth are of comparative interest to Rosaceae crops. To encompass this a range of BBCH scales were cross-referenced to increase utility of a scale for *Gillenia*, including the generalised scales for perennial plants and weed species, and the crop-focused scales of Rosaceae pome fruits, stone fruits, strawberries and raspberries [1].

S.1.1 Considerations for *Gillenia trifoliata*

Gillenia is a rhizomatous perennial herb and each season, significant vegetative growth of main and lateral shoots arises from each of the perennating rhizome buds prior to reproductive development. Significant biomass and energy is associated with development of the full aerial shoot system, most of which has reproductive fate. To encompass these phenotypic aspects, a three-digit BBCH scale was needed to describe key events occurring

on both main and lateral shoots, which is in contrast to two-digit scales that tend to focus only on main shoot development. Furthermore, the whole rhizome context influences development of each perennating bud and the shoot it bears, leading to differential shoot vigour and fate. To accommodate this, the 3-digit BBCH scale describes growth stages of a single shoot and allows for differences in vigour by enabling designation and description of growth of as many axillary nodes as required.

For flower and fruit development it is more practicable to have an organ-level scale, as one may prefer to normalise sampling based on organ phenotype rather than shoot phenotype. For example, flowers may be sampled or assessed just before petal tips visible or upon opening, and fruit when near full size or at a specific date after pollination. Therefore, for reproductive principle growth stages 5-8, an organ-level scale with organ-level descriptions is given and assigned a two-digit code to recognise the position-independent nature of organ development. For reproductive growth stages, the organ-level stage informs the shoot-level scale in being a combination of a scale-relevant percentage and the phenotype of the respective organ-level stage.

Shoot- and organ-level phenological growth stages are summarised in Supplementary Table 1. Full descriptions of shoot- and organ-level growth stages follow with representative figures for key stages. Fig. 1 shows a graphical overview of principle growth stages in relation to time based on data from three multi-stemmed individuals, although it is anticipated that different environmental conditions and genetic backgrounds would affect these timings.

S.1.2 Whole plant phenology

Consistent with the rhizomatous perennial herb habit, all aerial shoots senesced at the end of autumn leaving only the rhizome to undergo winter dormancy. In this study, winter conditions were simulated with controlled cold storage of 12 weeks at 5°C. After simulated winter, new shoots arose from the perennating buds borne upon the woody rhizome which were situated at or above the soil surface. Buds on the same plant progressed at variable rates, suggesting different developmental age or genetically-programmed vigour. All buds across the three multi-stemmed individuals were broken within two weeks after the end of dormancy. Notably, prior to establishment in the above conditions, plants were held without simulated overwintering and correspondingly vegetative growth was stunted and reproductive development was rare.

Most perennating rhizome buds developed into reproductive shoots. Of these, one or two shoots per rhizome exhibited greater vigour; termed ‘primary shoots’ (Supplementary Fig. 1a,b). The remaining floral shoots, termed ‘secondary shoots’ exhibited a reduced rate of growth, but both primary and secondary shoots followed a double sigmoidal growth curve (Supplementary Fig. 1b). The first growth phase of vigorous primary shoots was steeper than secondary shoots, such that by the first phase growth plateau of secondary shoots, primary shoots had attained 90% total height (stage 309) while secondary shoots had only reached 74% total height (~stage 307). Node number increased with shoot height; the final number of nodes of primary shoots ranged from 11–16, whereas secondary shoots ranged from 8–11 (Supplementary Fig. 1c). Correspondingly, nodes before flowering were also greater on primary shoots (9–11) compared to secondary shoots (4–8)

(Supplementary Fig. 1e). A small proportion of shoots were vegetative and showed low vigour in terms of both total height and final node number.

Primary shoots were the first to exhibit lateral shoot growth. The timing of this broadly correlated with 50% total shoot height (28 DAD, stage 305, Supplementary Fig. 1b,d). On both primary and secondary shoots two phases of lateral shoot growth were observed (Supplementary Fig 1d). The first occurred on the distal one- to two-thirds of the main shoot and was predominantly reproductive fate. A second phase occurred late-in-season from proximal nodes and was predominantly vegetative fate but with sporadic occurrence of flowering.

Shoot vigour and a high shoot-to-rhizome ratio influenced the window for inflorescence emergence and flowering. Within shoot, the window was a function of number of reproductive nodes and number of flowers within each inflorescence. Both of these variables developed at different rates. Inflorescence and flower development progressed basipetally down the shoot, and individual flowers developed at different rates within each inflorescence. Between shoots, flowering commenced earlier on primary shoots than secondary shoots. Fruit development lasted approximately 120-130 days after pollination, and concluded with fruit senescence which lasted 30 days.

S.1.3 Shoot-level BBCH scale

Principle Growth Stage 0: Bud development

In the generalised BBCH scale for perennial plants, bud development begins with winter dormancy (stage 00), enters bud swelling (stages 01–03) and bud break (stage 07), and ends with buds showing leaf tips (stage 09) [1], and this progression was recapitulated in *Gillenia* rhizome buds. During dormancy, buds appeared brown and dry, and this appearance remained up to 7 days after dormancy (DAD; stage 000, Supplementary Fig. 2a). Buds swelled and reddened between 5-10 DAD (stages 001-003, Supplementary Fig. 2b). Bud break began between 9-12 DAD (stage 007, Supplementary Fig. 2c) and ended with emergence of green to red-orange leaf tips around 14-16 DAD (stage 009, Supplementary Fig. 2d).

Principle Growth Stage 1: Leaf development (main and lateral shoots)

Leaf development in perennial plants begins with first leaves separating (stage 10) and ends when leaves at all nodes are unfolded (stage 19) [1]. In *Gillenia*, a single three-leaflet compound leaf developed at each node of the main shoot and became separately discernible as the shoot elongated during concomitant principle growth stage three (shoot development). First leaves separated from the main shoot (stage 100) between 15–18 DAD (Supplementary Fig. 3a) and were rapidly followed by first leaves unfolding on the main shoot (stage 101, 19-21 DAD, Supplementary Fig. 3b). The end of leaf development on main shoots, described by leaves at all nodes unfolded (stage 109), was dependent on shoot vigour, but was typically concluded by 44–48 DAD. Leaves also occurred at each node of lateral shoots. Timing, progression and order of lateral shoot development is described in principle growth stage 2, but leaf development upon lateral shoots can be designated for axillary nodes 1–9 using stages 111–199.

Principle Growth Stage 3: Shoot development (main and lateral shoots)

The principle growth stage of shoot development in perennial plants begins with the first visible signs of stem elongation on the main shoot (stage 30) and ends when shoots have reached 90% total height or length (stage 39) [1]. In *Gillenia*, the first visible signs of main shoot elongation (stage 300) occurred soon after bud break (15-18 DAD, Supplementary Fig. 3a) and soon after the first node was detected (stage 301, 19-21 DAD, Supplementary Fig. 3b). From then, shoot development was rapid and reached approximately 30% of total shoot height (stage 303) between 23-25 DAD. At this time, shoot architecture was columnar with rigid shoots supporting erect partially unfolded yellow-red leaves (Supplementary Fig. 3c). Approximately 40% total shoot height (stage 304) was reached between 26-28 DAD and shoot architecture had become more rounded as shoots loosened and green leaves unfolded (Supplementary Fig. 3d). Shoot development reached the approximate mid-way point (50% of total shoot height, stage 305) between 27-30 DAD (Supplementary Fig. 3e), and at this stage, inflorescence emergence and lateral shoot development had begun (Supplementary Fig. 3e, arrows and arrowhead). Throughout shoot growth, shoot development followed a predominantly monopodial pattern with very occasional instances of sympodial growth. The end of shoot development (stage 309), when main shoots ceased to elongate further, occurred between 68-74 DAD. Shoot elongation also occurred on lateral shoots. Timing, progression and order of lateral shoot development is captured by principle growth stage 2, but lateral shoot elongation can be designated for axillary nodes 1-9 using stages 311-399.

Principle Growth Stage 2: Lateral shoot development (main shoot only)

In the generalised scale for crops and weeds, this stage describes side shoot formation for monocots, dicots, and perennial plants and tillering for Gramineae species [1], and is frequently omitted from published scales. The stage was included here to allow description of the order and number of lateral shoots arising for the main shoot independent of node position, and is included alongside description of specific events upon lateral shoots within each principle growth stage. Offering two mechanisms accommodates the different patterns of development. In the first and main phase of lateral growth, lateral shoots emerged from within the distal one- to two-thirds of the main shoot. Here, lateral growth was predominantly acropetal with some divergent order at proximal nodes (later, these proximal nodes bore the longest lateral shoots giving an overall pyramidal architecture to the distal shoot). To accommodate the divergent growth order, the scale defines lateral shoot growth in terms of first, second, third, (etc.) lateral shoots visible rather than node order. In contrast, events occurring upon lateral shoots treated within specific principle growth stages are assigned in distal-proximal node order to match the basipetal order of development.

In this study, the first lateral shoot (stage 200) was observed on the most vigorous primary shoots when those shoots had reached approximately 50% total shoot height (stage 305) between 25–27 DAD (Supplementary Fig. 3e (arrowhead)). By the first growth phase plateau of primary shoots, ~48 DAD, all lateral shoots of the first and main phase of lateral growth were present. The second phase arose from the proximal main shoot when

main shoots had completed growth, between 68–74 DAD. Growth of lateral shoots was predominantly monopodial, however, like main shoots, had very occasional instances of sympodial growth.

Principle Growth Stage 4: Development of vegetatively propagated or perennating organs

This stage could be assigned to growth and development of the rhizome including development of next-seasons perennating rhizome buds. In the strawberry BBCH scale this stage was used for stolon development, and in plants with cane habits such as raspberry this stage was omitted. While a detailed study of the vegetative propagation capacity is of considerable interest, this research was not undertaken in this study.

Principle Growth Stage 5: Inflorescence emergence (main and lateral shoots)

The generalised scale for perennial plants, describes inflorescence emergence succinctly in three growth stages: inflorescence visible (stage 51), first individual flowers visible (still closed; stage 55), and first flower petals visible (stage 59) [1]. In the BBCH scales for pome fruit and stone fruit, the first half of the inflorescence emergence principle growth stage is coincident with bud development principle growth stage 0, such that early stages of inflorescence emergence of the reproductive pome or stone fruit bud track begin with bud swelling and end with bud burst of green leaf tips [1]. First visibility of floral buds for pome and stone fruit are set at stage 55, like the generalised plant scales. The first stage of inflorescence emergence in strawberry is also stage 55, when the first floral buds are visible at the bottom of the rosette [1]. For raspberry, visibility of the first individual flowers was assigned to stage 51. For *Gillenia*, despite the similarity in growth habit between *Gillenia* and raspberry, the approach of the majority of published scales was followed.

In their study of floral ontogeny in several Maleae species and *Gillenia*, Evans & Dickinson [3] identified the first stages of floral development in *Gillenia* as appearing after short shoots had expanded after winter. The floral transition and subsequent primordial inflorescence development may therefore occur during early shoot development. Determination of the timing of the floral transition and early inflorescence development is generally outside the scope of BBCH scales which tend to focus on macroscopic phenotypic events, but the data of Evans & Dickinson [3] was in keeping with observations made here. Just before most shoots of the rhizome had reached the mid-way point of their development a change in shoot architecture and leaf morphology was observed. At around 26–28 DAD, plant shoot architecture shifted from erect and columnar to loose and rounded (Supplementary Fig. 3c,d). At the shoot apex, feathery leaves clustered loosely around primordial inflorescence units indiscernible by eye (Supplementary Fig. 3e, Supplementary Fig. 4a). This change in morphology early in shoot development may signal a refocus from vegetative to reproductive development, and therefore putatively serves as a representative phenotype to define early inflorescence emergence, stage 501, but would require further investigation. This stage would align with inflorescence bud swelling of pome and stone fruit, signalling the beginning of inflorescence maturation after dormancy.

Shortly after the shoot architecture change (stage 501), floral structures became discernible but remained enclosed by folded leaves. At the shoot apex, particularly on vigorous shoots, multiple floral buds from the terminal and most distal nodes were clustered tightly pushing the enclosing leaves aside (stage 505, 29–31 DAD,

Supplementary Fig. 4b,c). The *Gillenia* inflorescence is a highly branched panicle in which pedicles and peduncles elongate during emergence. Panicle elongation separated individual flower buds and a small petal tip was discernible on the most advanced flower (stage 506, 35–37 DAD, Supplementary Fig. 4d). Individual flowers then enlarged and separated further, and most had a short thin tube of petal tissue visible, defining growth stage 507 (39–41 DAD, Supplementary Fig. 4e). At the end of inflorescence emergence at the terminal node most flowers were near full size and petal tubes were broader and beginning to unfurl (stage 509, 42–44 DAD, Supplementary Fig. 4f). The window of inflorescence emergence of a vigorous primary shoot spanned ~27 days from 27 DAD (stage 500) to 54 DAD (stage 599).

Principle Growth Stage 6: Flowering (main and lateral shoots)

For perennial plants, the principle growth stage of flowering begins when the first flowers open sporadically (stage 60), is followed by a 'full flowering' stage during which 50% of flowers are open (stage 65), and ends when all petals have fallen or dried and in some cases fruit set is visible (stage 69) [1]. This progression was recapitulated in *Gillenia*, with the exception that fruit set was dependent on outcrossing owing to self-incompatibility and so was not used here.

In *Gillenia*, the first flowers opened on terminal inflorescences from 44 DAD (Supplementary Fig. 5a). Flowering progressed rapidly to stage 603 when 30% of flowers had stamens slightly revealed by 48–DAD (Supplementary Fig. 5b). Due to basipetal order of development, flowers on lateral shoots at proximal nodes were developmentally younger, for example, 20% of flowers were open at nodes one to five (stages 612-652), compared to 10% of flowers open on lateral shoot at node 6 (stage 661, Supplementary Fig. 5b,c). The mid-way point of flowering on the main shoot (stage 605) occurred when 50% of flowers had petals ~90° to the hypanthium with stamens light-coloured and well-revealed (Supplementary Fig. 5b). Near the end of flowering, 68-74 DAD, all petals had fallen or were easily displaced from flowers on main shoot (stage 609) and all flowers borne upon lateral shoots had opened to expose stamens (stage 695; Supplementary Fig. 5d). The window for flowering of a vigorous primary shoot spanned ~30 days from 44 DAD (stage 600) to 74 DAD (stage 699).

Principle Growth Stage 7: Fruit development (main and lateral shoots)

The fruit development stage in the BBCH scales for weed species and generalised perennial plants centre around fruit size [1]. The pome fruit scale is similar and details stages important to crop management. In early stages, fruit diameter is specified and stages are aligned with seasonal fruit falls. Later the scale specifies fruit size in relation to final size to allow for cultivar differences. A hybrid approach is used for *Gillenia* to capture changes in phenotype which might align dry versus fleshy fruit development.

The shoot level scale for fruit development defines stages based on the organ-level scale (described below) across the whole shoot context. Fruit development began (stage 700, 0 DAP) from 48 DAD with sporadic fruit set of stage 65 flowers of the terminal inflorescence after out-crossing pollination. Early stages of the shoot-level scale were based on receptacle and follicle enlargement. However, follicle final size was reached early in fruit development, ~21 DAP, limiting the utility of fruit size as a further phenotypic indicator. Instead, later

stages were defined by seed coat colour change, requiring destructive analysis. The total period of fruit development spanned ~130 DAP before signs of fruit senescence were seen. The window of fruit development of a vigourous primary shoot was ~160 days, from 48 to 208 DAD, given the long flowering window.

Principle Growth Stage 8: Fruit Senescence (main and lateral shoots)

For pome fruit and also generalised perennial plants, the principle growth stage of ripening is focussed on colour development in early stages and, in later stages, variety specific ripening indicators for picking for shipment or immediate consumption [1]. In weed species, the stage is briefly defined by the beginning of ripening (stage 81) and fully ripe (stage 89). The term fruit senescence is used here as the processes that leads to seed release are more akin to leaf senescence while ripening refers to processes which are distinct to fleshy fruit. The shoot level scale for fruit senescence defines stages based on the organ-level scale (described below) across the whole shoot context. Fruit senescence began (stage 800) after 130 DAP (~178 DAD) and lasted approximately 30–40 days.

Principle Growth Stage 9: Vegetative senescence (main and lateral shoots)

Given the annual cane-like growth exhibited by *Gillenia*, remobilisation of nutrients to the roots to bring on senescence forms a key developmental stage. The first signs of senescence begin as soon as 68–70 DAD, which coincided with both the end of shoot development and flowering. Signs of senescence began with browning of distal ends of mature leaves, typically at more basal nodes, but generally not following a strict directional progression. Leaves continued to brown throughout the whole plant and remained attached to the stem until late in the growing season. Leaves eventually detached from shoots, while shoots themselves retained strength appearing woody close to the rhizome. At stage 999 all shoots had senesced appearing brown and dry, but remained attached to the rhizome, requiring manual excision prior to transfer to cold storage.

S.1.4 Reproductive Organ-level BBCH scale

Organ-level stages for reproductive principle growth stages were defined with reference to BBCH scales for other Rosaceae species [1].

Principle Growth Stage 5: Inflorescence emergence

Inflorescence emergence organ-level stages are depicted in Supplementary Fig. 6a-e. Stage 51 inflorescence was apparent as feathery leaves enclosing primordial inflorescences, not readily visible by eye. Stage 55 inflorescence had discernible buds of different developmental age covered by leaves with a representative organ stage of a small green bud of less than 2 mm diameter at the hypanthium. Stage 56 had a larger diameter at the hypanthium of 2-3 mm and petals tips had emerged. Stage 57 floral buds were 3-4 mm in diameter at the hypanthium, with occasional red colouration, and a long pointed tube of white petals. Stage 59 was set to align as close as possible with the pome fruit BBCH scale, defined as 'most flowers with petals forming a hollow ball' [1], commonly termed the 'balloon' stage. Stage 59 was therefore defined by the long pointed tube of petals broadening and about to unfurl. At the organ level, the window of inflorescence emergence, from stage 51 to 59 was approximately 15 days.

Principle Growth Stage 6: Flowering

Flowering organ-level stages are depicted in Supplementary Fig. 6f-k. Stage 60 floral buds were characterised by petals unfurling and parting at the tip but with hypanthium opening remaining constricted. Stage 63 petals were nearing 90° to hypanthium and stamens are slightly revealed. Stage 65 flowers were open, with light-yellow stamens well revealed and petals approximately 90° to the hypanthium. Petals on stage 67 flowers had begun to fade and stamens were visibly darkened. At the end of flowering, petals on stage 69 flowers had fallen or were easily displaced. At the organ level, the window of flowering, from stage 60 to 69 was approximately 12 days.

Principle Growth Stage 7: Fruit development

Fruit development organ-level stages are depicted in Supplementary Fig. 7a-f. Initially, no immediate phenotypic indicators of successful pollination were apparent, because in both pollinated and unpollinated flowers the receptacle became dark-green and the hypanthium reddened. By 6 DAP fruit set was evident as enlargement of the receptacle, compared to a stage 65 open flower, and follicle enlargement was confirmed after removal of the hypanthium. A noticeable increase in follicle length was observed without need for removal of the hypanthium by 14 DAP. Stage 71 was designated as receptacle enlargement to accommodate both destructive and non-destructive approaches, and the stage was putatively aligned with ~10 DAP.

As mentioned, final fruit size was reached early in development by ~21 DAP (Supplementary Fig. 8a-c). Fruit did not begin to senesce until 130 DAP suggesting an additional 110 days of fruit development not evident in external follicle phenotype. Therefore, stage 72 was designated as follicle length reaching final size (~21 DAP), and other phenotypic attributes were used to define later stages.

Beyond 21 DAP, changes in external fruit appearance do not help to define further stages. For instance, fruit colour was highly variable, did not correlate with light exposure by hypanthium presence/absence, and was irrespective of developmental age. Instead phenotypic changes after 21 DAP occurred at the seed level (Supplementary Fig. 7f). Seed coat colour was silver-white at 21 DAP, then transitioned to yellow shortly after with an approximately 50:50 ratio of silver-white to yellow observed at 28–29 DAP, and defined stage 73. Biosynthesis of red pigmentation occurred between 50–60 DAP, which developed as an orange hue across the seed coat, marking stage 75. By 100 DAP, the entire seed coat had reached the final dark red-brown colour, marking stage 79.

Principle Growth Stage 8: Fruit senescence

Fruit senescence organ-level stages are depicted in Supplementary Fig. 9a-i. The beginning of fruit senescence is not immediately evident in external fruit appearance. However, by ~120 DAP seed coat colour had reached deep red-brown and did not appear to change beyond this, thus defining fruit maturity, stage 80 (Supplementary Fig. 7b). Soon after, follicles could be made to split along ventral and dorsal sutures by applying gentle force upon the frontal plane, suggesting partial maturation of dehiscence zone tissue layers. Not all follicles split

easily down the entire follicle suture, instead some only split easily at the apex then tore the follicle wall thereafter, suggesting ongoing maturation and basipetal formation of dehiscence zones. The phenotype of splitting follicle sutures under gentle force was used to define stage 81, the first signs of fruit senescence.

Fruit senescence continued with apical dehiscence defining stage 85, which occurred 120–130 DAP. Follicle walls began to dehiscence along ventral and dorsal sutures defining stage 87, approximately 130–140 DAP. The senescence process caused follicles to shrink in length and to bend outward from the central fruit axis generating both an increase in follicetum width and reduction in follicle length and receptacle width (Supplementary Fig. 8a-c). Between 140–160 DAP, follicles were mostly brown throughout, had extended further outwards from the central fruit axis and seeds were easily displaced from the dried follicetum signalling the end of senescence, stage 89. The window of fruit senescence was approximately 30–40 days.

S.1.5 Correlations between growth stages

For many plants, including *Gillenia*, the ten principle growth stages occur concomitantly rather than sequentially. The guidance for usage of overlapping scales [1] recommends that concomitant stages can be referenced together separated by diagonal strokes e.g. 305/501, or alternatively, if only one stage can be referenced it may either be the more advanced growth stage or the growth stage of greater interest. Complexities of overlapping plant development can therefore be handled according to research goals.

Further, complexities of overlapping phases of plant development can become useful tools to help describe stages with more accuracy and thus aid in data collection. Midway points in growth stages are sometimes difficult to observe in real-time because they are described as a proportion of the endpoint e.g. 50% of total shoot height reached for stage 305. To maximise utility of the BBCH scale, correlations were identified which may help to identify stages in real-time. Correlations are summarised in Supplementary Table 2.

References

1. Meier, U.E., *Growth stages of mono- and dicotyledonous plants: BBCH monograph*. 2001: Federal Biological Research Centre for Agriculture and Forestry.
2. Boyes, D.C., et al., *Growth stage-based phenotypic analysis of Arabidopsis: a model for high throughput functional genomics in plants.* Plant Cell, 2001. **13**(7): p. 1499-510.
3. Evans, R.C. and T.A. Dickinson, *Floral Ontogeny and Morphology in Gillenia ("Spiraeoideae") and Subfamily Maloideae C. Weber (Rosaceae).* International Journal of Plant Sciences, 2005. **166**(3): p. 427-447.
4. Vurture, G.W., et al., *GenomeScope: fast reference-free genome profiling from short reads.* Bioinformatics, 2017. **33**(14): p. 2202-2204.
5. Robinson, J.T., et al., *Juicebox.js Provides a Cloud-Based Visualization System for Hi-C Data.* Cell Syst, 2018. **6**(2): p. 256-258 e1.
6. Jung, S., et al., *Whole genome comparisons of Fragaria, Prunus and Malus reveal different modes of evolution between Rosaceous subfamilies.* Bmc Genomics, 2012. **13**.
7. Moyano, E., et al., *Genome-wide analysis of the NAC transcription factor family and their expression during the development and ripening of the Fragaria x ananassa fruits.* PLoS One, 2018. **13**(5): p. e0196953.
8. Chagne, D., et al., *The draft genome sequence of European pear (Pyrus communis L. 'Bartlett').* PLoS One, 2014. **9**(4): p. e92644.
9. Zhang, L., et al., *A high-quality apple genome assembly reveals the association of a retrotransposon and red fruit colour.* Nat Commun, 2019. **10**(1): p. 1494.
10. Li, R., et al., *Genomewide analysis of homeobox gene family in apple (Malus domestica Borkh.) and their response to abiotic stress.* J Genet, 2019. **98**.
11. Su, H., et al., *Genome-wide analysis and identification of stress-responsive genes of the NAM-ATAF1,2-CUC2 transcription factor family in apple.* Plant Physiol Biochem, 2013. **71**: p. 11-21.
12. Busatto, N., et al., *Climacteric ripening of apple fruit is regulated by transcriptional circuits stimulated by cross-talks between ethylene and auxin.* Plant Signal Behav, 2017. **12**(1): p. e1268312.

Supplementary Figures

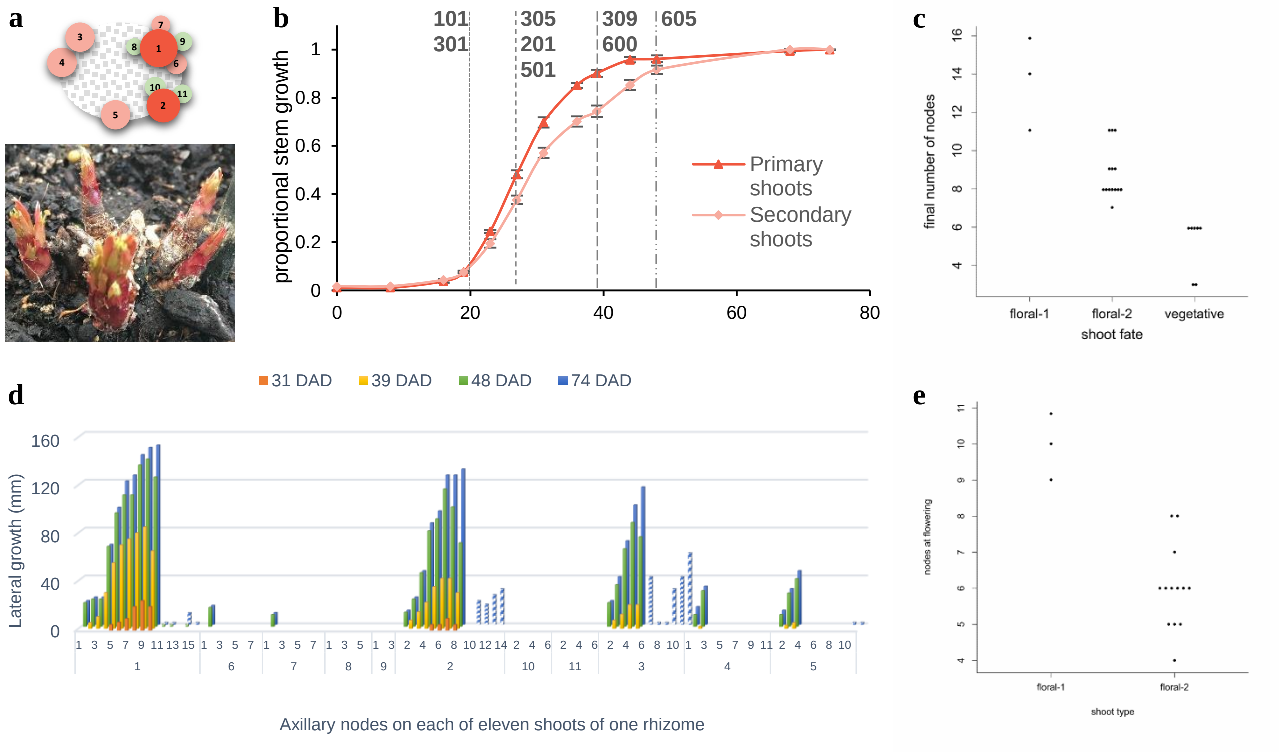

Supplementary Figure 1. Aerial shoot system development

a) Schematic of a rhizomatous plant used in this study. Primary reproductive shoots (red), secondary reproductive shoots (pink), and vegetative shoots (green) with size of circle depicting overall shoot vigour based on shoot height. Numbers relate to shoot numbering in (d). Schematic based on plant at bottom, ‘shoot 1’ at front. b) Growth curves of primary and secondary reproductive shoots with some key developmental BBCH stages marked on graph. c) Final number of nodes in the growth season analysed by shoot fate (floral or vegetative) and shoot vigour (primary, floral-1; secondary, floral-2). d) Lateral growth from axillary nodes of eleven shoots on one plant at 31, 39, 48, and 74 DAD. Shoots with reproductive fate (solid bars) and vegetative fate (dashed bars). Numbering of shoots relates to the numbering shown in (a). e) Number of nodes before flowering were greater on primary floral shoots (floral-1) compared to secondary floral shoots (floral-2).

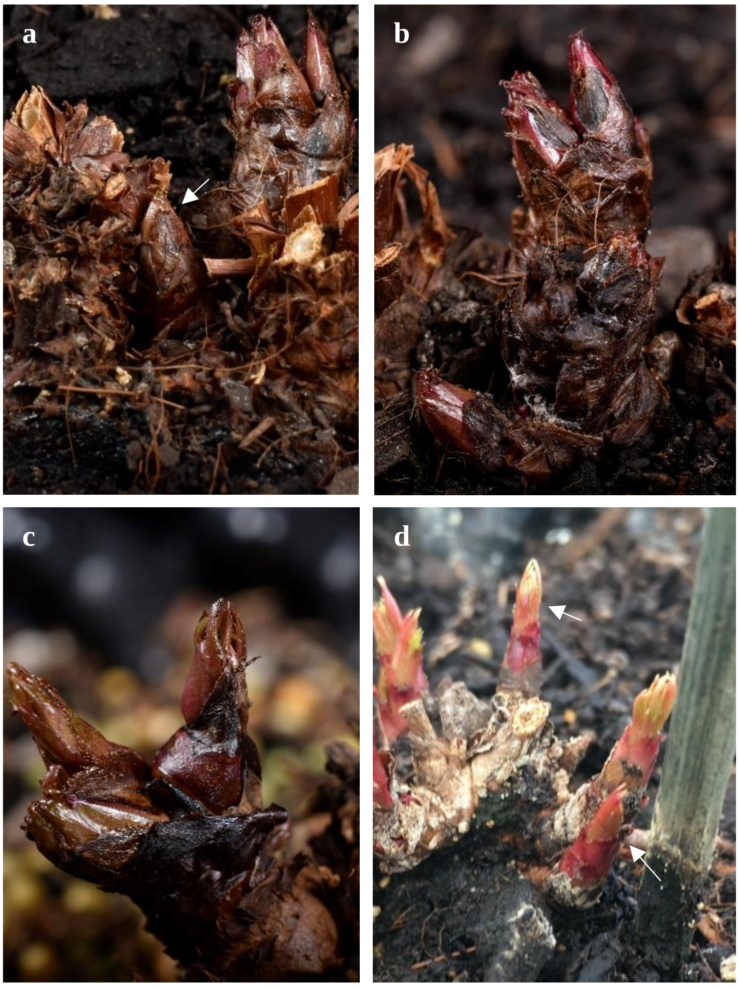

Supplementary Figure 2. Principle growth stage 0: bud development

a) Dormancy (stage 00); rhizome buds appear brown (arrow) during dormancy and up to 7 days after removal from cold storage. b) Bud swelling; buds become swollen and red between the beginning (i, stage 01) and end (ii, stage 03) of bud swelling occurring between 5-10 days after cold storage (DACS). c) Bud break (stage 07) begins when bud tips elongate and separate, from 9-12 DACS. d) Bud development ends (stage 09) when light-green to red-orange leaf tips emerge from bud scales (arrow), between 14-16 DACS.

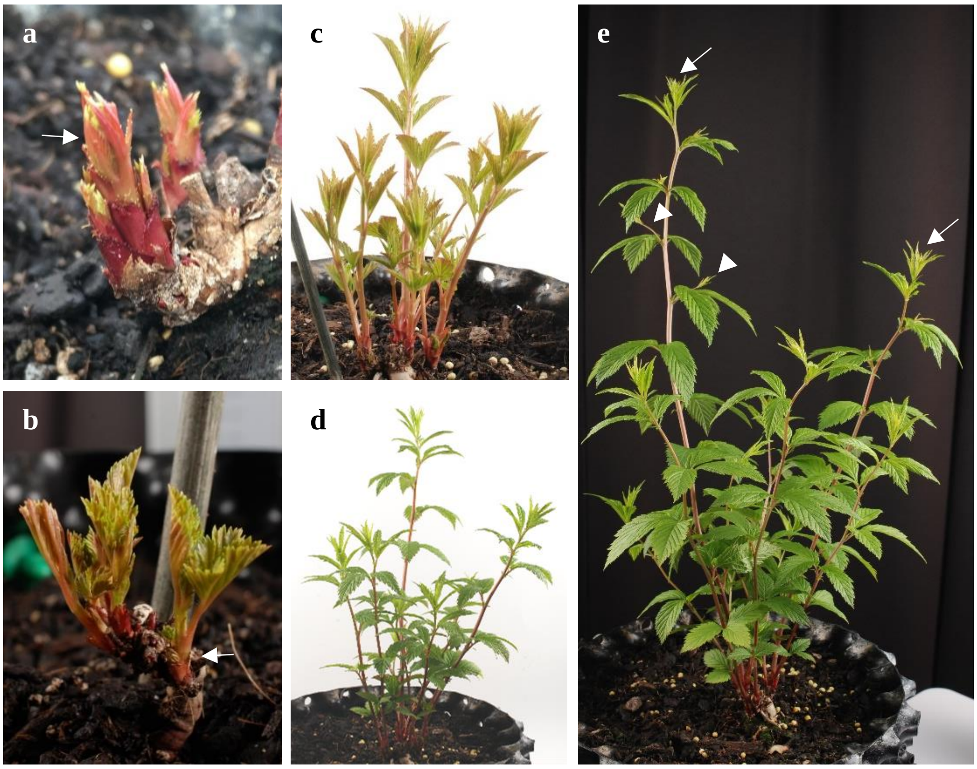

Supplementary Figure 3. Principle growth stages 1-3: leaf, lateral, and main shoot development

a) First leaves separate (stage 100) as internodes begin to elongate (stage 300) after 15-18 days after cold storage (DACS). b) First leaves unfold (stage 101) and first node is detectable (stage 301, arrow) after 19-21 DACS. c) Shoot development (stage 303), approximately 30% of total shoot height, occurred between 23-25 DACS. Architecture appears columnar with erect yellow-reddened leaves. d) Shoot development (stage 304), approximately 40% of total shoot height, occurred between 26-28 DACS. Architecture is more loose and less erect, as shoots elongate and leaves unfold. e) Approximate half-way point of shoot development (stage 305) occurred between 29-30 DACS. At this time, inflorescence emergence (stage 501; arrows) and lateral shoot development (stage 200/201, arrowheads) had begun.

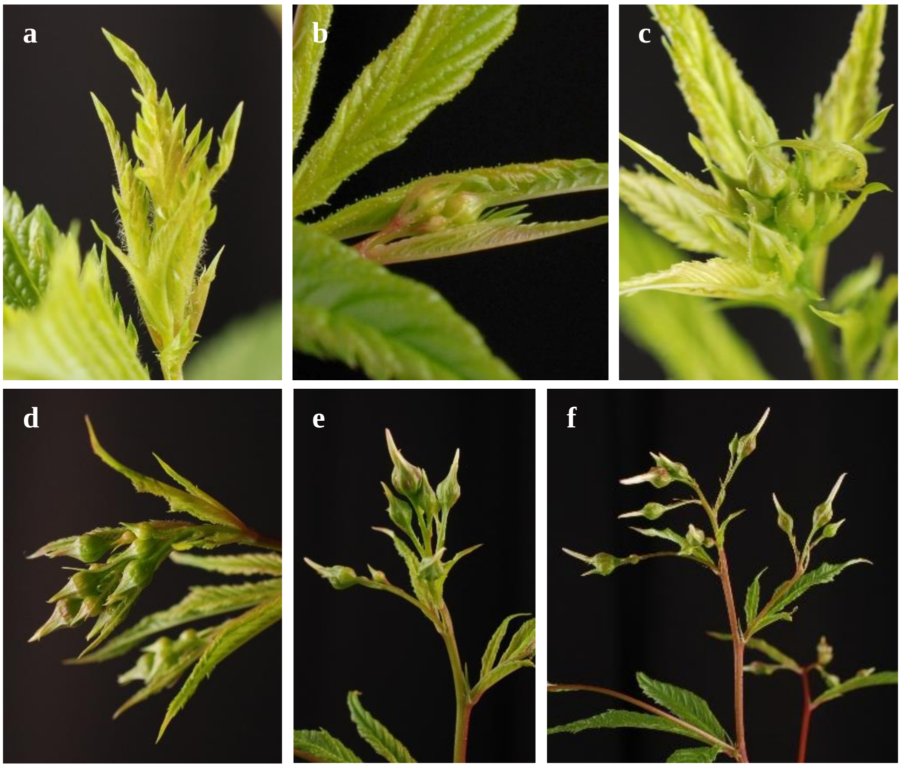

Supplementary Figure 4. Principle growth stage 5: inflorescence emergence

a) A terminal node showing feathery leaves enclosing primordial inflorescences not visible by eye (stage 51/501). b) Individual flowers visible beside leaves on a lateral shoot from nth node (stage 5N5, N = nth node). c) Individual flowers visible amongst unfolded leaves on a vigorous main shoot (shortly after stage 505). Flower buds clustered together on terminal and most distal nodes which will separate further with panicle elongation and shoot internode elongation. d) Flowers separating with panicle elongation (stage 506). e) Most individual flowers enlarged and separated with petals visible (stage 507). f) Inflorescence on main shoot at stage 509 with most flowers forming a broad tube of petals (arrow) and inflorescence structures on lateral shoots from first and second nodes (stage 518 and 528 respectively) with some flowers with petals visible (~57) and some flowers with broad tube of petals (~59).

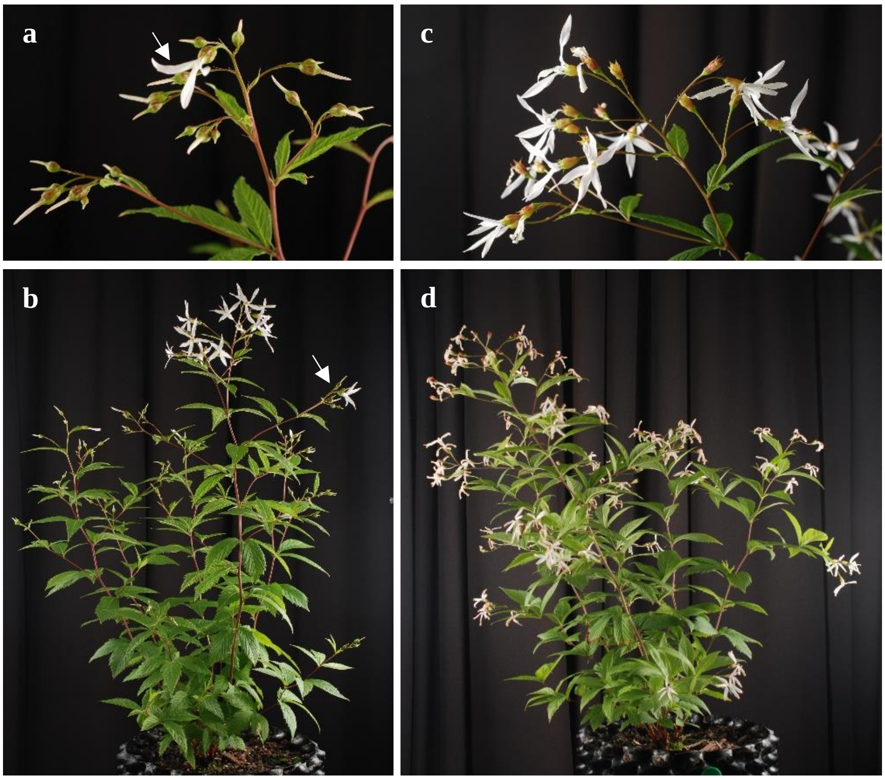

Supplementary Figure 5. Principle growth stage 6: flowering (main and lateral shoots)

a) First flowers open sporadically on main shoot (stage 600). Organ-level stage 60 flower with petals parting (arrow), most remaining flowers at organ-level stage 59. b) Stage 605, 50% of flowers open (main shoot terminal node). Flowering on lateral shoots at proximal nodes were developmentally younger with 30% of flowers open, stage 613, 623, 633, 643, 653 at nodes 1, 2, 3, 4, 5, respectively, and 10% of flowers open on lateral shoot at node 6 (stage 661, arrow). c) Stage 607 (main shoot) with 70% petals fallen or easily displaced and all flowers opened. Flowers on lateral shoots at nodes 1 and 2 developmentally younger at stages 616-626. d) Nearing end of flowering. All petals fallen or easily displaced from flowers on main shoot (stage 609) and all flowers borne upon lateral shoots opened to expose stamens (stage 695).

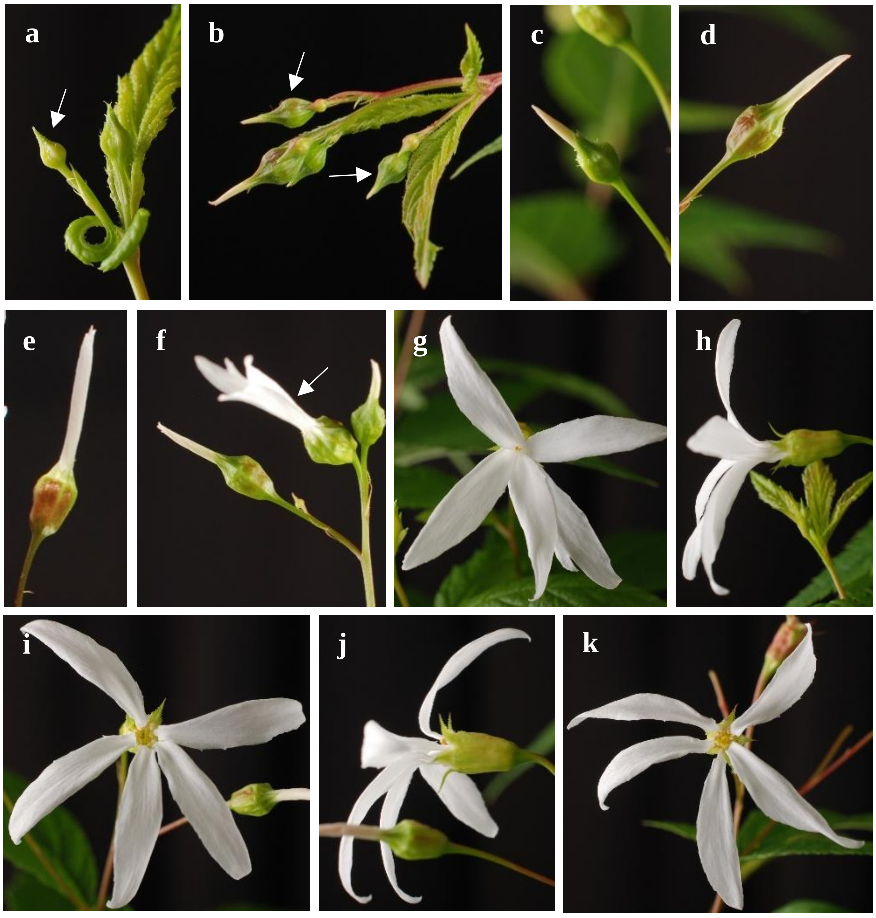

Supplementary Figure 6. Organ-level growth stages for inflorescence emergence and flowering

Individual flowers become discernible, and grow and enlarge during principle growth stage 5, inflorescence emergence (a-e), then mature and open for pollination during principle growth stage 6, flowering (f-k). a) Stage 55, green bud (arrow), hypanthium diameter up to 2 mm. b) Stage 56 (arrows), green buds with petal tips emerged, hypanthium 2-3 mm diameter. c) Stage 57, bud 3-4 mm diameter, pointed tip of petals. d) Stage 58, bud 3-4 mm diameter, hypanthium reddened, longer pointed tip of petals. e) Stage 59, petal tube broadening and about to unfurl. f) Stage 60 (arrow), petals parting, hypanthium opening constricted. g-h) Stage 63 flower, front (g) and side (h) view, petals <90° to hypanthium, stamens slightly revealed. i-j) Stage 65 flower, front (i) and side (j) view, petals ~90° to hypanthium, stamens light and well revealed. k) Stage 67 flower, petals fading, stamens dark.

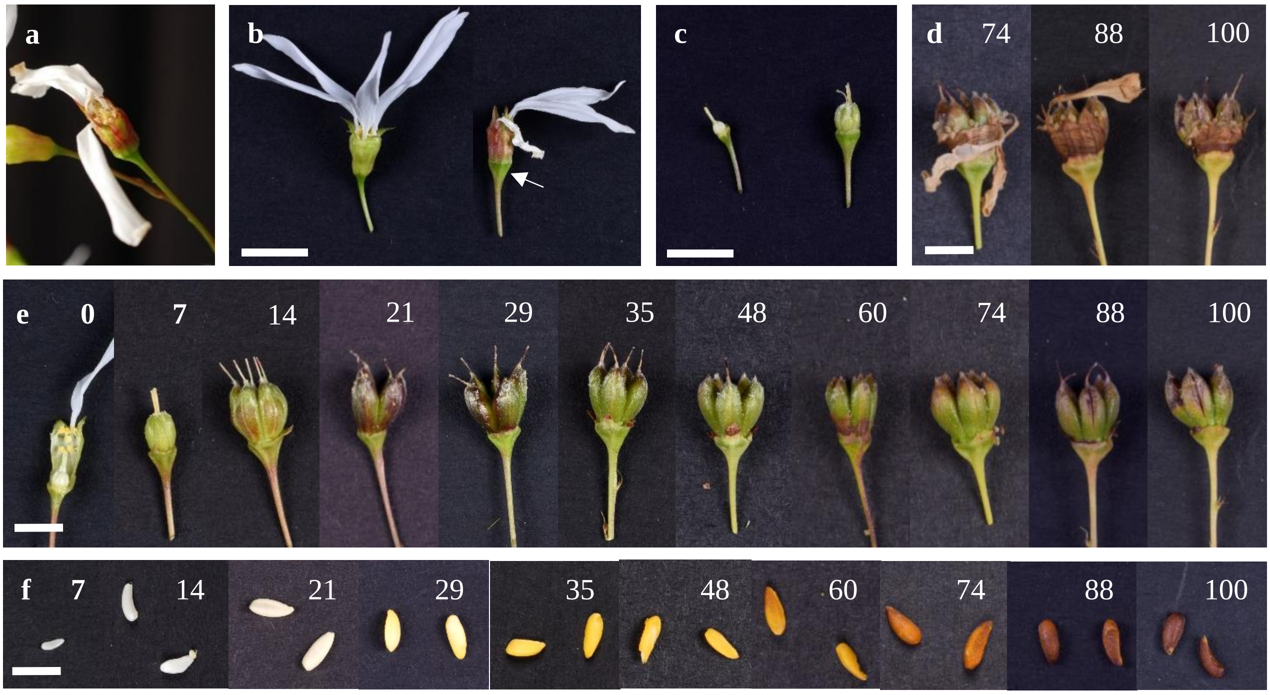

Supplementary Figure 7. Organ-level growth stages for fruit development

a) Unpollinated flower at end of flowering (stage 69) with petals falling, hypanthium becoming red, and receptacle becoming dark green. b) Open flower (stage 65, left) and pollinated fruit 6 days after pollination (DAP; right) showing enlargement of the receptacle in pollinated flowers (arrow); scale 1 cm. c) Flowers in (b) with hypanthium removed. Unpollinated (left), 6 DAP (right), growth of follicle evident upon removal of the hypanthium; scale 1 cm. d) The hypanthium dries, and parts but remains attached throughout fruit development, DAP indicated; scale 5 mm. e) Fruit development series of folliceta with dried hypanthium removed, DAP indicated; scale 5 mm. f) Seed coat colour development series with DAP indicated; scale 5 mm.

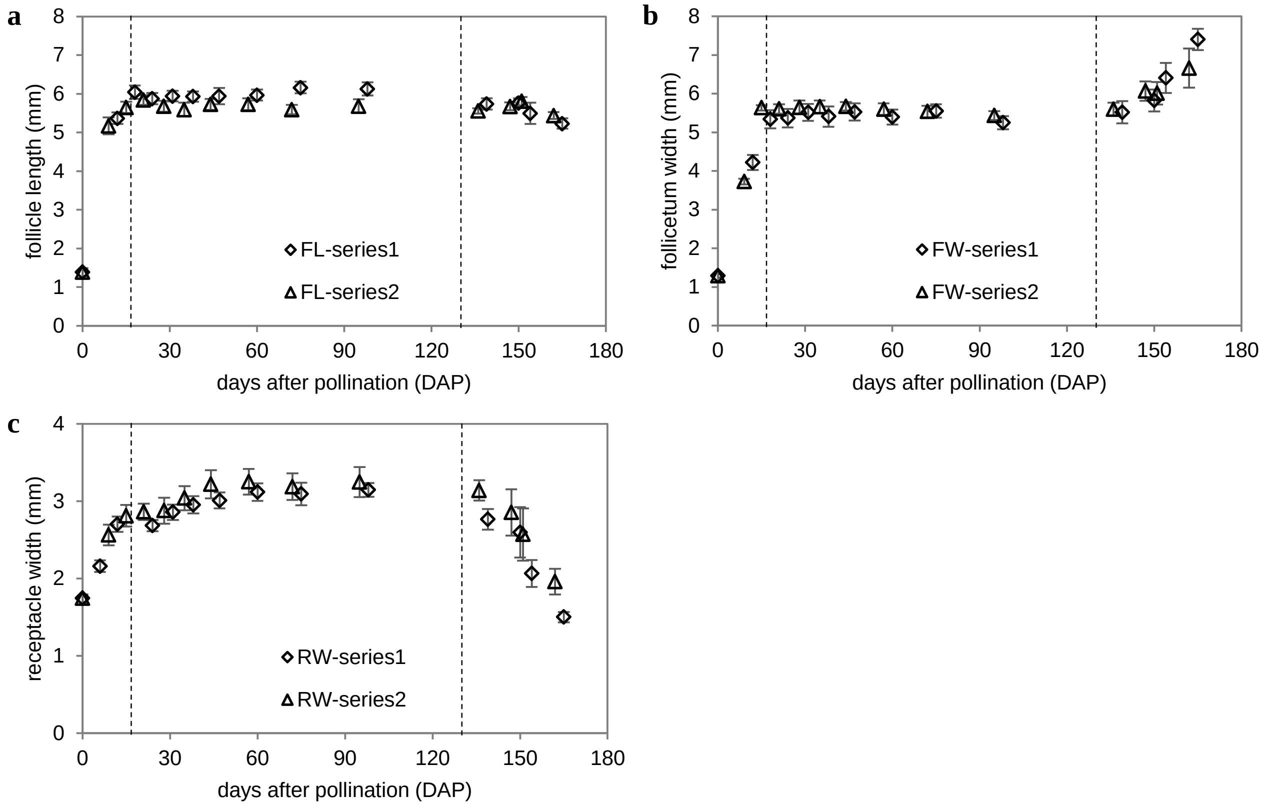

Supplementary Figure 8. Fruit development growth curves

Growth of (a) follicle length, (b) follicetum width, and (c) receptacle width over fruit development from two series of fruits (n=7) pollinated on different days. Dashed line at 20 DAP represents stage 72 when follicle length reaches final size. Dashed line at 130 DAP represents putative beginning of fruit senescence.

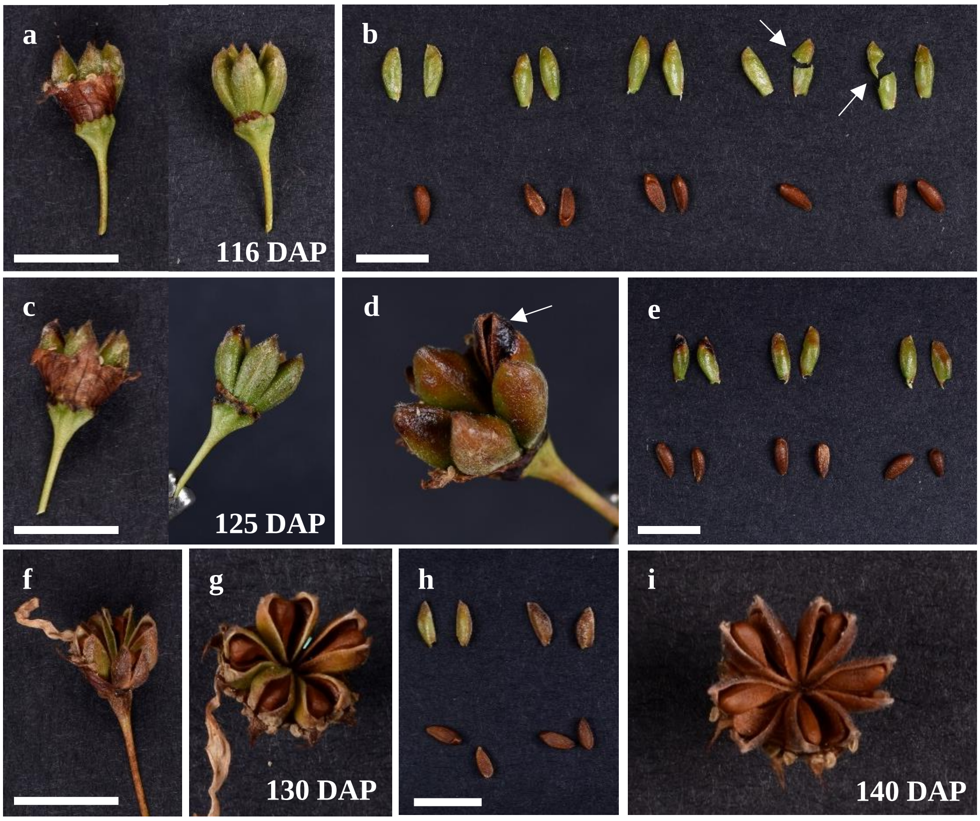

Supplementary Figure 9. Organ-level growth stages for ripening

a) Fruit with (left) and without (right) hypanthium at organ-level ripening stage 81 (110-120 DAP). Signs of senescence not evident in external fruit appearance, but some follicles can be split along suture length under gentle force. The seed coat has reached final red-brown colour. Scale, 1 cm. b) Fruit in (a) separated into individual follicles. Three split follicles on left easily split along full suture length with gentle force, whereas the two split follicles on right split easily with gentle force only at the follicle apex, tearing the follicle wall thereafter (arrows). Follicles held 1-2 seeds each. Scale, 1 cm. c) Fruit with (left) and without (right) hypanthium in the early stages of ripening (125 DAP), with one follicle showing darkened brown colour and splitting at apex only. Scale, 1 cm. d) Top view of fruit in (c) showing splitting and dark brown colour at apex (arrow). e) Fruit in (c) separated into individual follicles (only three follicles shown). All follicles separated easily. Seeds remained red-brown (bottom). Scale, 1 cm. f) Fruit at 130 DAP with hypanthium attached all follicles dehiscent. g) Top view of fruit in (f). h) Separated follicles (top) from fruit in (f) showing range of colours of the fruit wall, from dark green to brown, and seeds (bottom) released from above follicles showing red-brown colour. i) Fully ripe follicetum at 140-160 DAP from a different individual positioned in a high light environment.

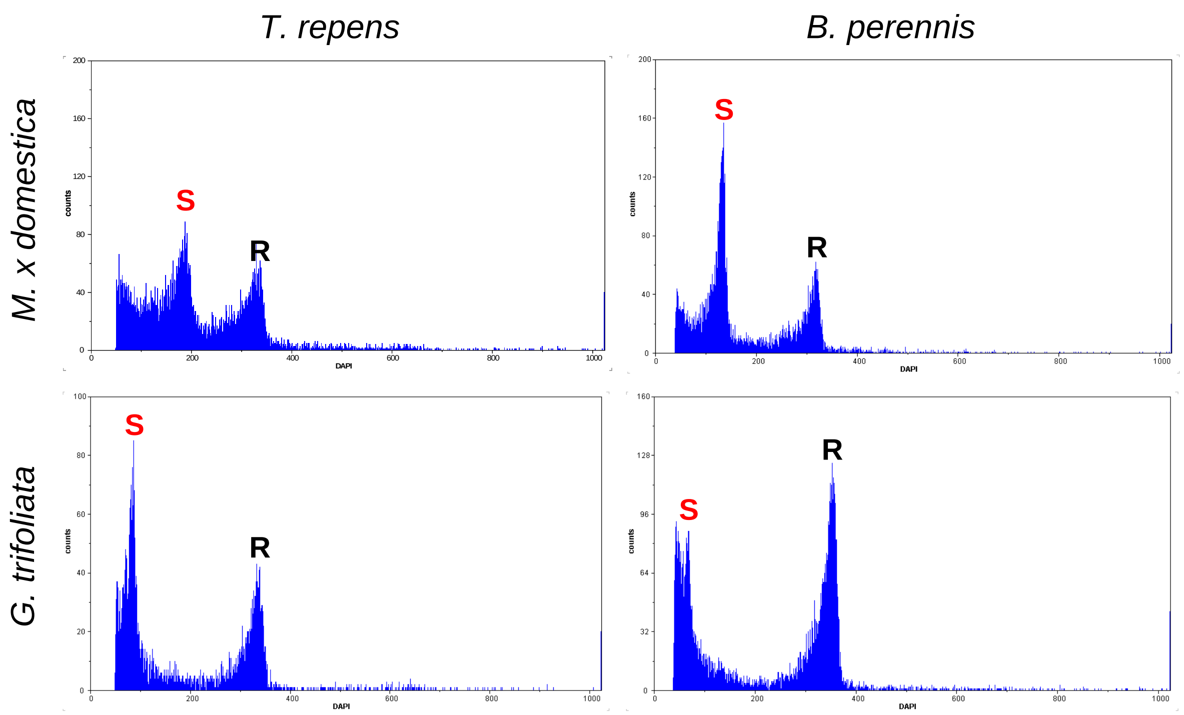

Supplementary Figure 10. Flow Cytometry Histograms

Flow-cytometry histograms showing cell counts of DAPI stained nuclei isolated from samples (S), apple (*M.* x *domestica*) and *Gillenia trifoliata*, with internal references (R), *Trifolium repens* and *Bellis perennis*.

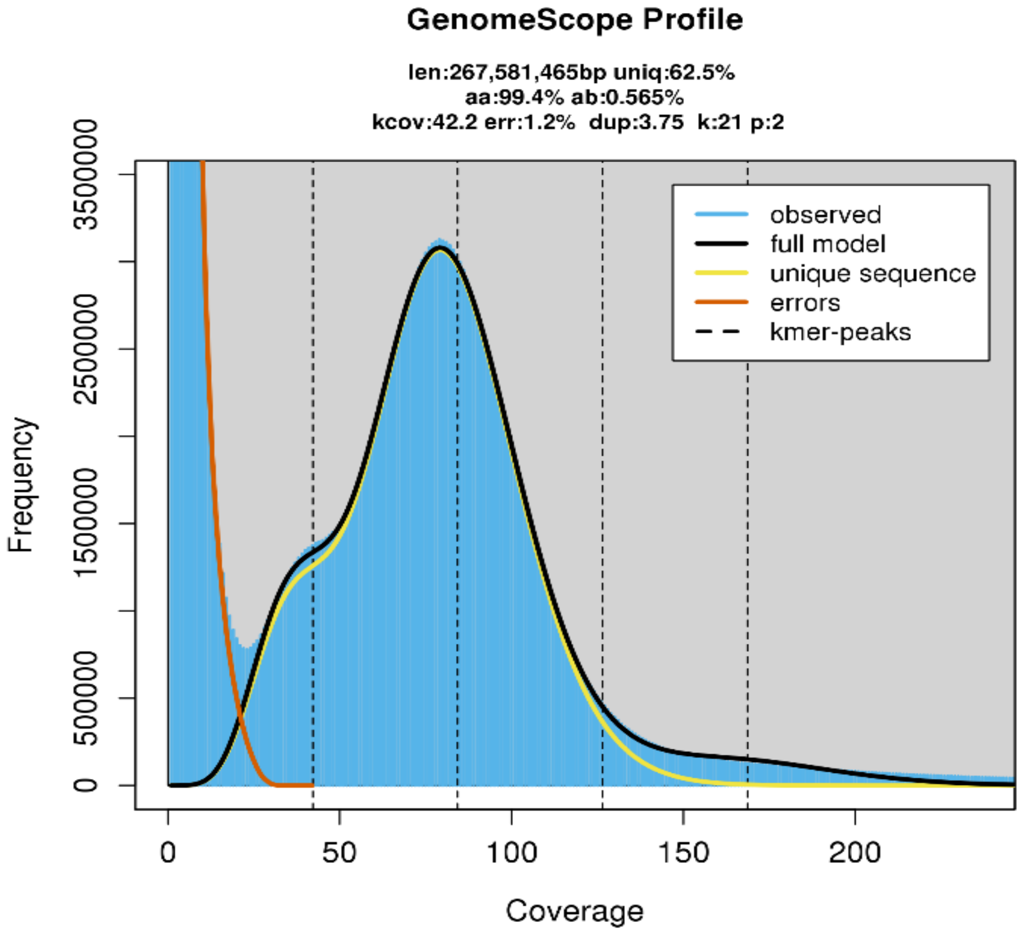

Supplementary Figure 11. K-mer distribution for estimation of genome size.

K-mer (k: 21) distribution produced with GenomeScope2 [4] using 10X Illumina short reads with estimation of genome size (len), homozygosity (aa), heterozygosity (ab), mean k-mer coverage for heterozygous bases (kcov), read error rate (err), the average rate of read duplications (dup), and ploidy (p).

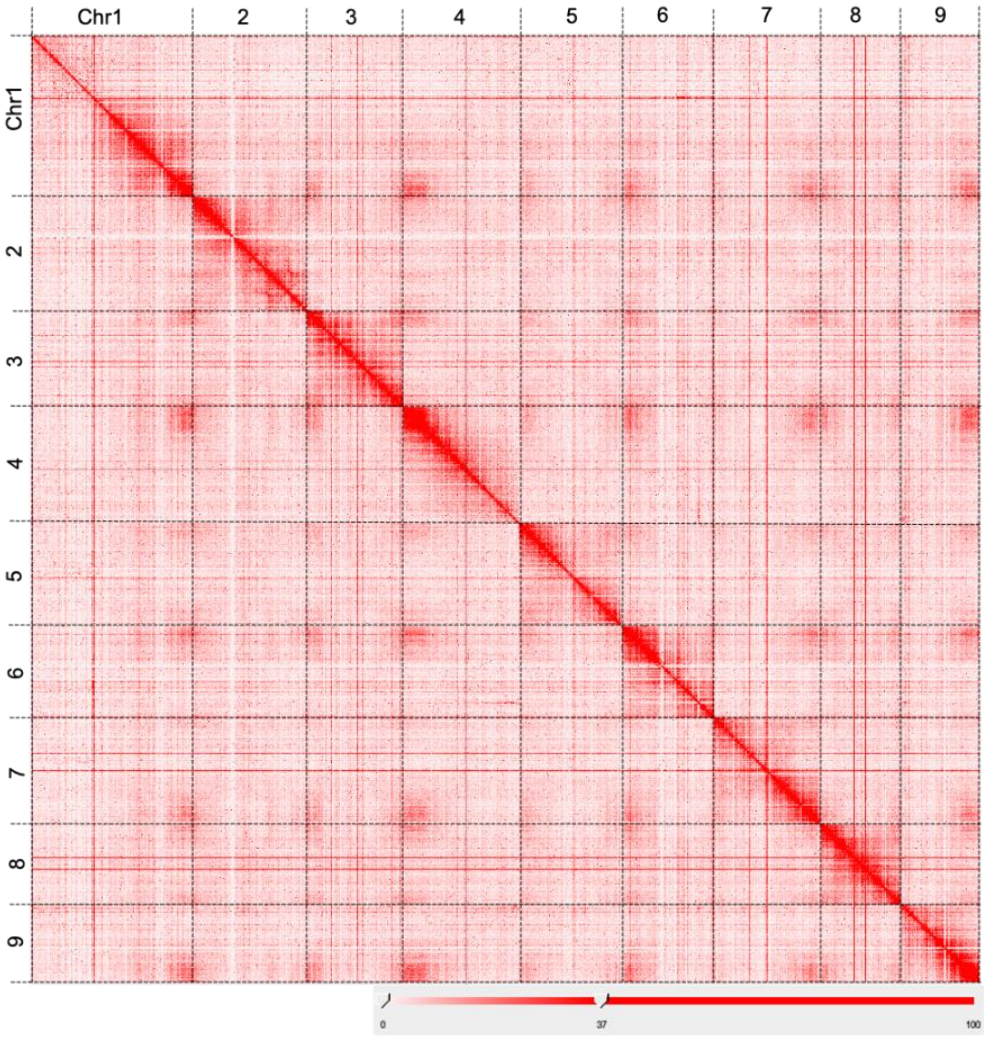

Supplementary Figure 12. Hi-C Contact Map

Hi-C contact map used for scaffolding *Gillenia* contigs into nine pseudo-chromosomes. The scale bar obtained from Juicebox [5] visualisation tool shows at least 37 interactions identified between the two chromosomal locations for each dark red dot.

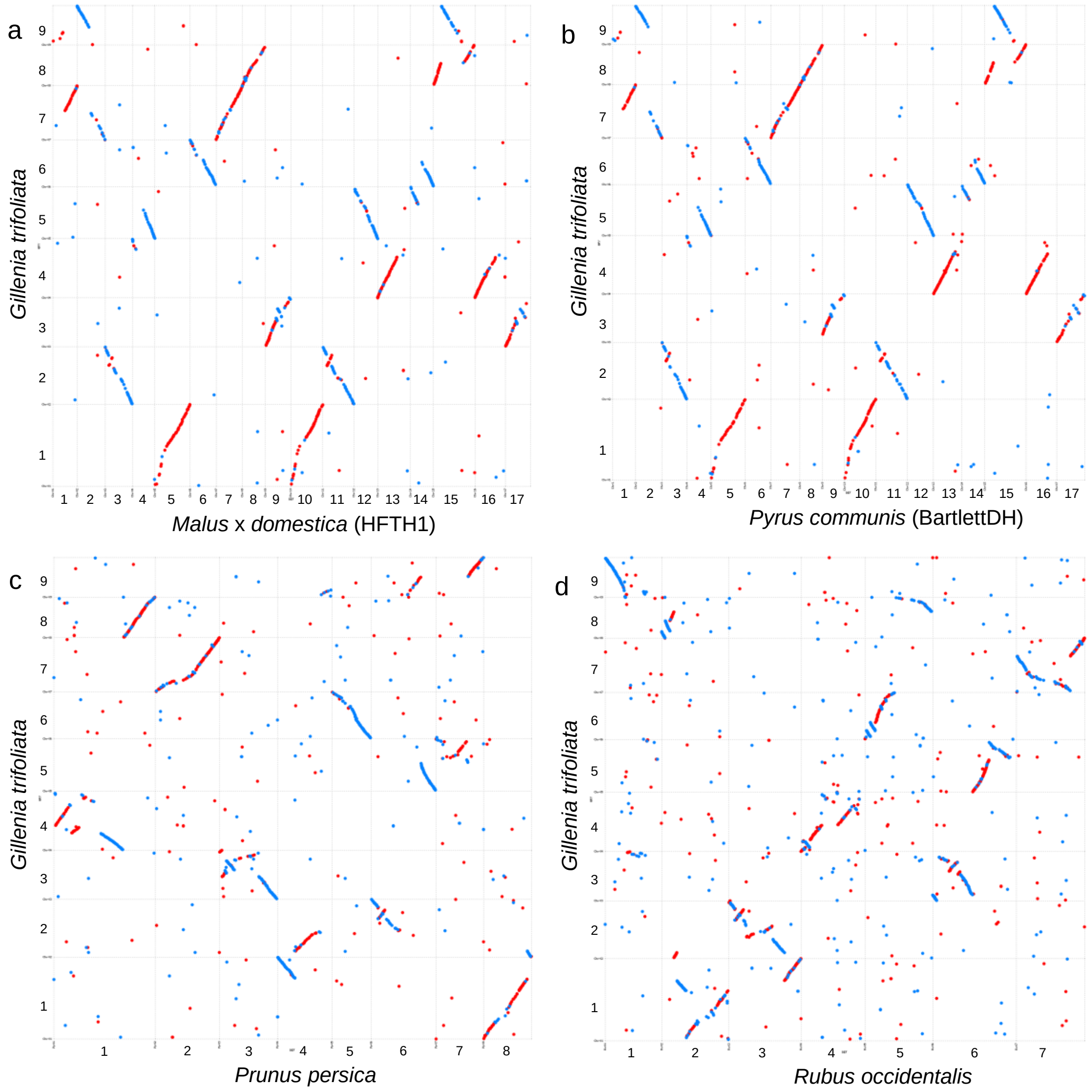

Supplementary Figure 13. Syntenic dot plots between *Gillenia* and Rosaceae species.

Syntenic dot plot of *Gillenia* chromosomes against (a) *Malus* (HFTH1), (b) *Pyrus* (BartlettDH), (c) *Prunus persica* (v2) and (d) *Rubus occidentalis* (v3). Each dot represents a collinear genomic block with at least 70% nucleotide identity over minimum length of 5 kb for apple and pear, and 2 kb and 1 kb for peach and raspberry, respectively. Red and blue represent forward and reverse matches respectively.

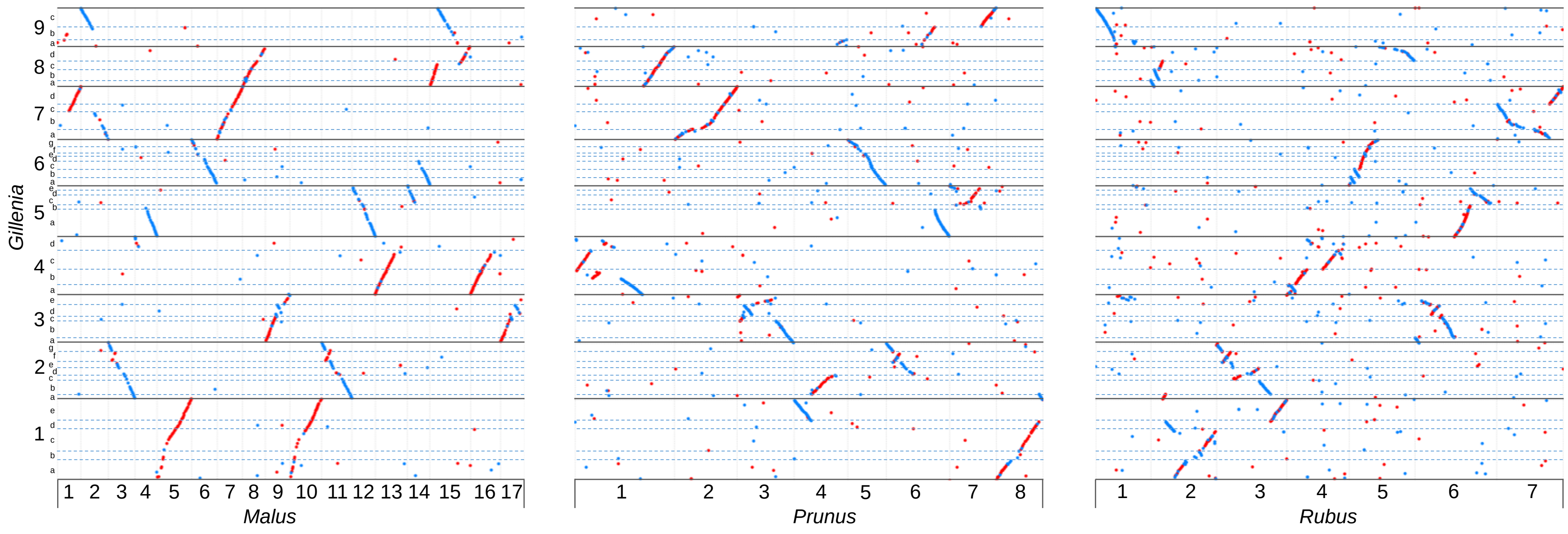

Supplementary Figure 14. Ancestral blocks detected from syntenic dot plots between *Gillenia* and Rosaceae species.

Syntenic dot plot of *Gillenia* chromosomes against Rosaceae species as described in Supplementary Figure 12. Forty four ancestral blocks detected across all three dot-plots shown as dotted blue lines.

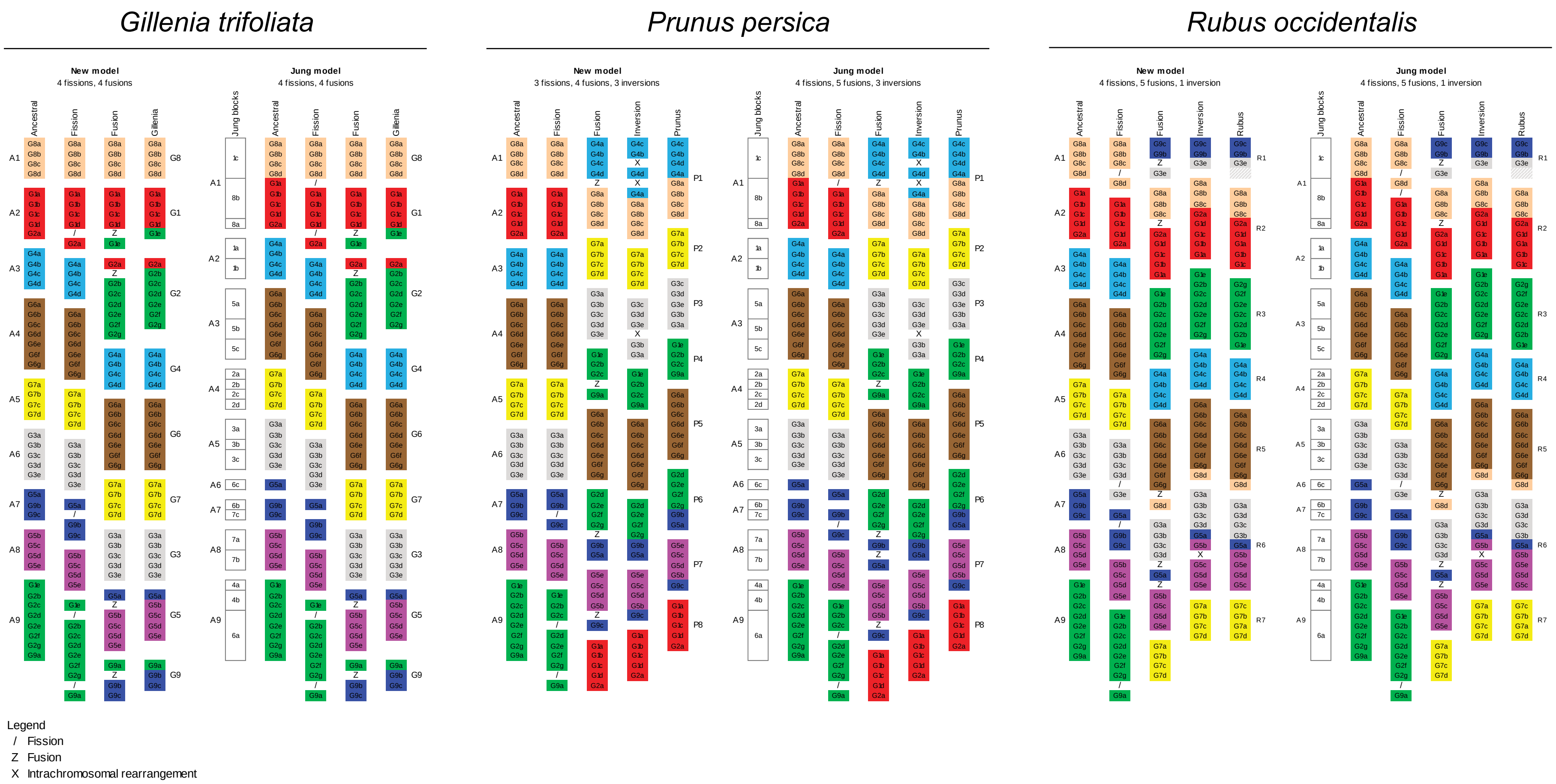

Supplementary Figure 15. Genome rearrangements predicted for present day Rosaceae species from common ancestor

Genomic rearrangements predicted for present day *Gillenia*, *Prunus*, and *Rubus* genomes based on the new ancestral genome model predicted in this study, compared to the ancestral model predicted by Jung et al [6]

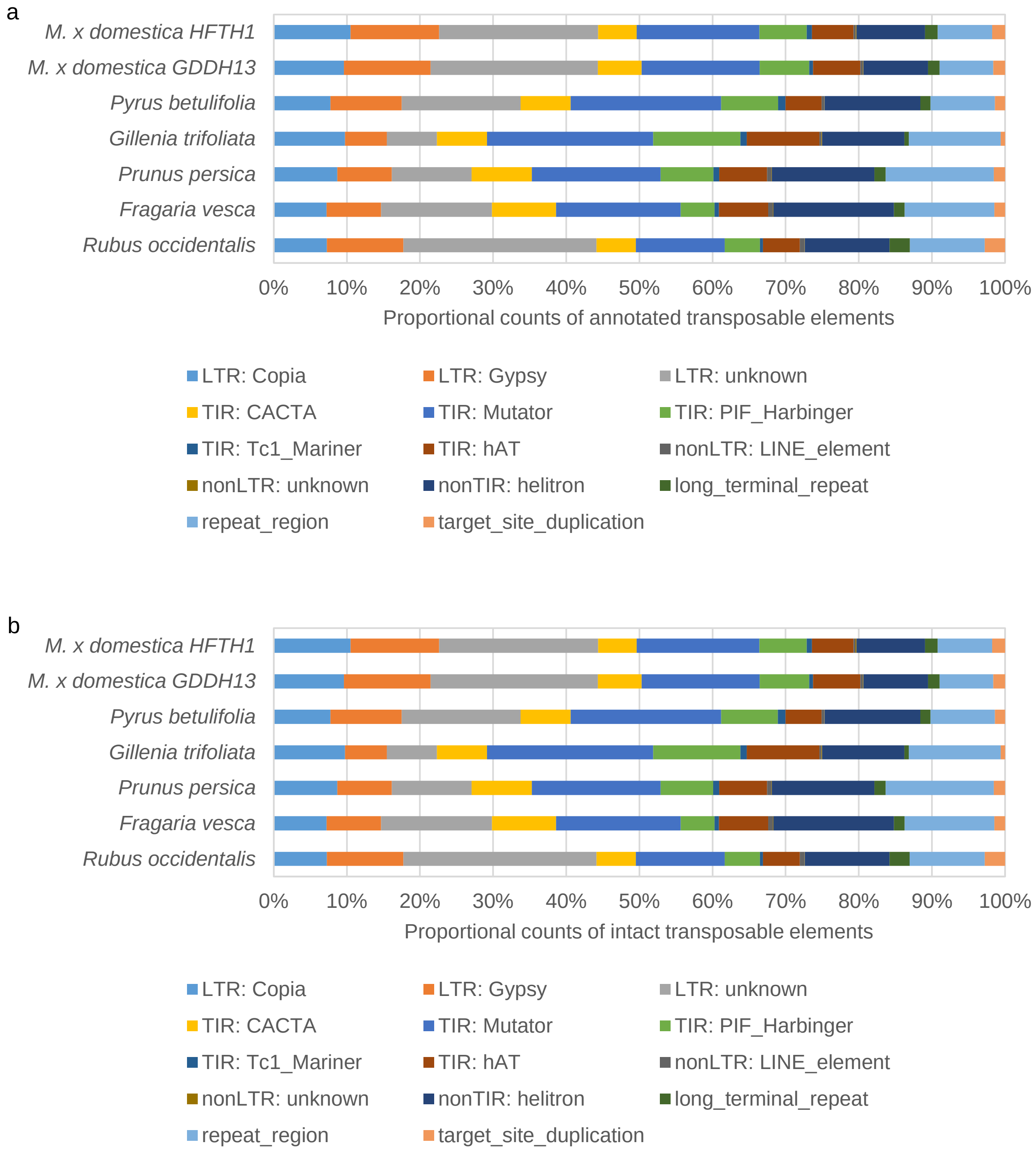

Supplementary Figure 16. Proportional counts of TEs in *Gillenia* and Rosaceae species.

Comparison of the proportional count of annotated (a) and intact (b) transposable elements between *Gillenia* and *Malus* (GDDH13 and HFTH1), *Pyrus betulifolia*, *Prunus persica*, *Fragaria vesca* and *Rubus occidentalis*.

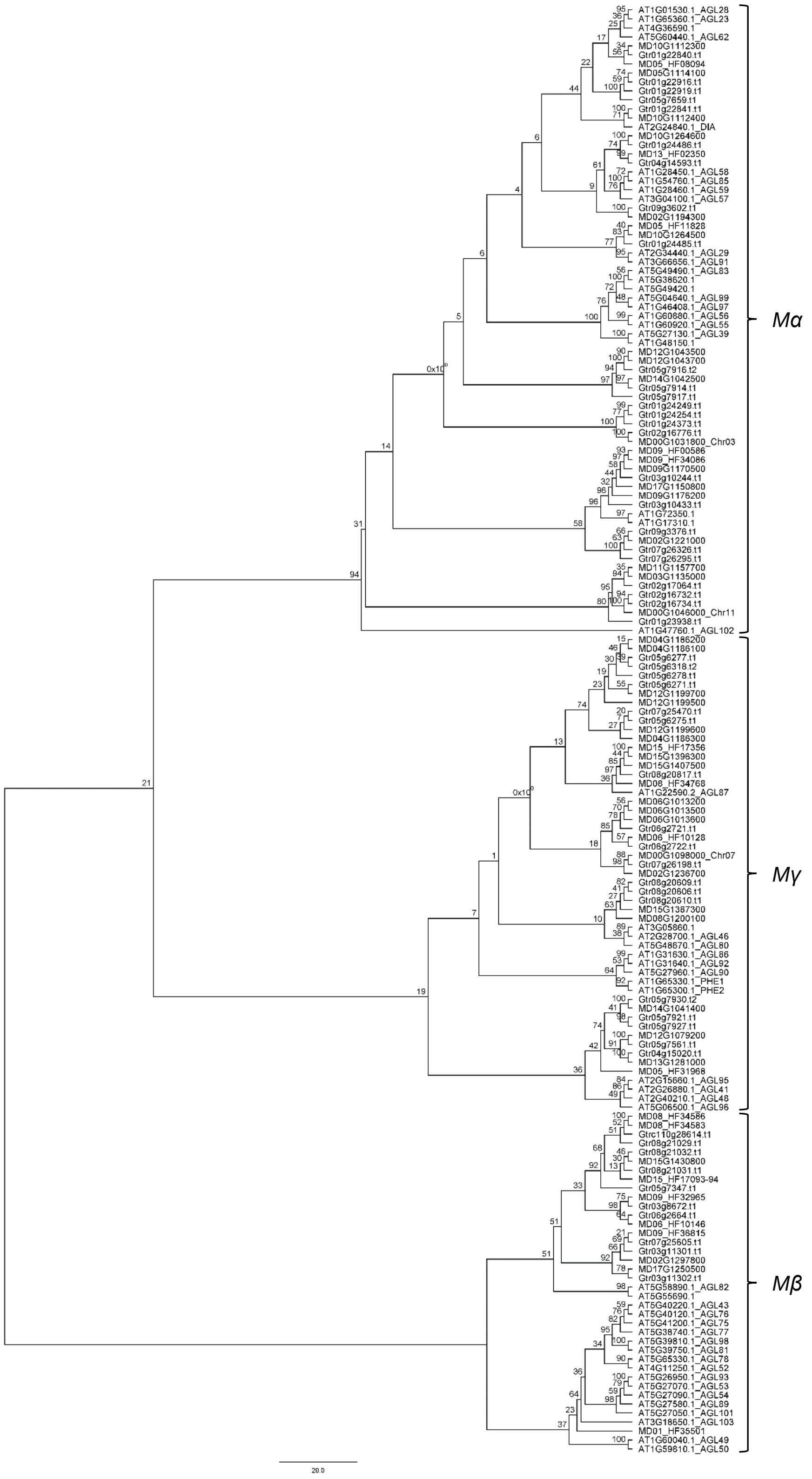

Supplementary Figure 17. Phylogenetic tree of Type I MADS transcription factors.

Phylogenetic tree of Type I MADS transcription factors in *Gillenia*, apple and *Arabidopsis*. Maximum likelihood bootstrap values from 100 datasets shown at branches. Prefixes: Gtr, *Gillenia trifoliata*; MDXXG, *Malus* GDDH13; MDXX_HF, *Malus* HFTH1; AT, *Arabidopsis thaliana*. MADS subfamily names follow standard convention. Scale bar shows substitutions per site.

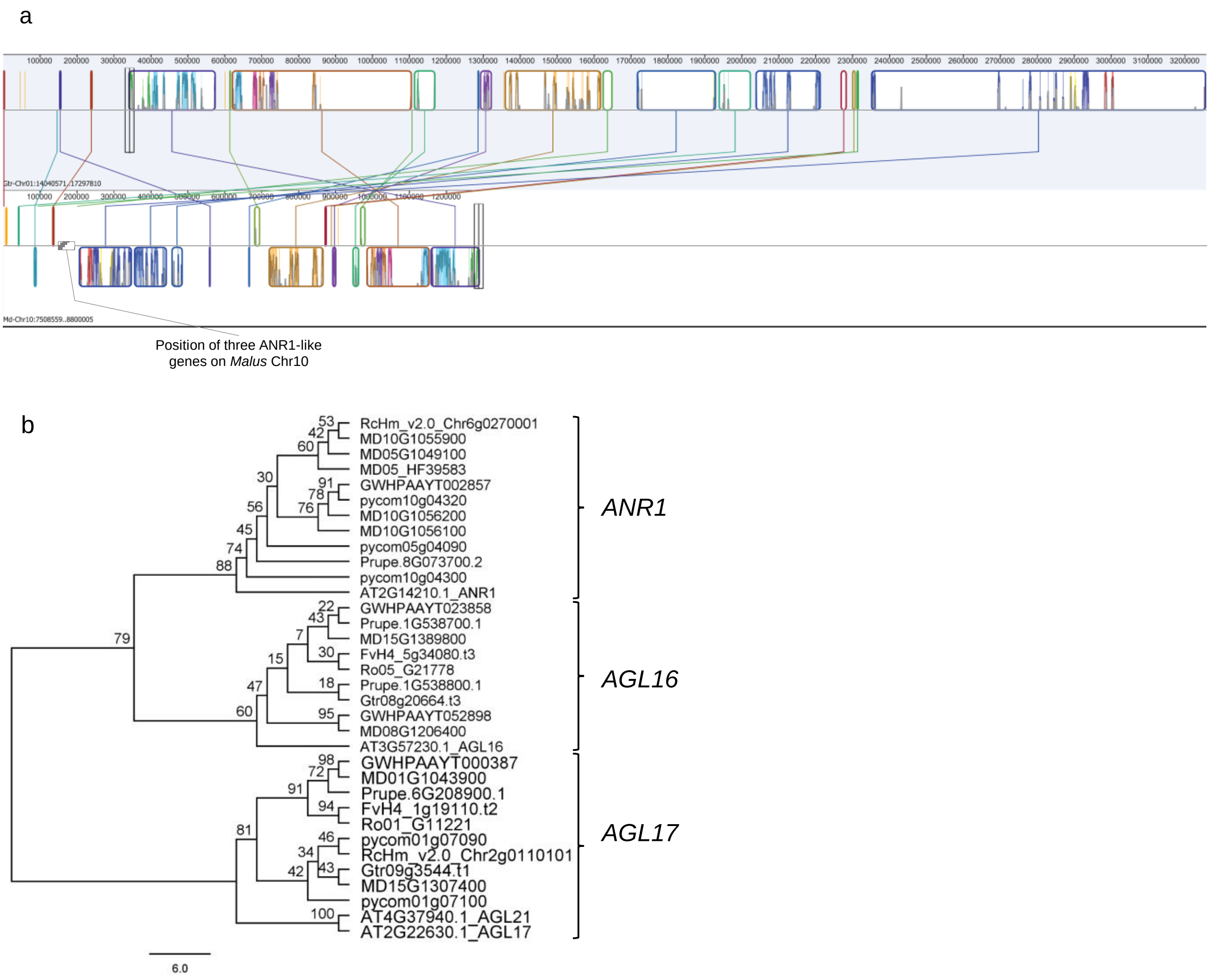

Supplementary Figure 18. *ANR1* locus

a) Progressive Mauve alignment of *ANR1* locus in *Gillenia* (top) and apple *GDDH13* (bottom). The locus of three *ANR1*-like genes in apple is positioned immediately proximal to the site of a large inversion in *Gillenia*. b) Phylogenetic tree of *ANR1* superclade with predicted proteins from Rosaceae species of interest including Gtr, *Gillenia trifoliata*; MDXXG, *Malus* GDDH13; MDXX_HF, *Malus* HFTH1; GWHP, *Pyrus betulifolia*; pycom, *P. communis* BartlettDH; Prupe, *Prunus persica* (v2); FvH4, *Fragaria vesca* v4.0a2; Ro, *Rubus occidentalis* (v3); RcHm, *Rosa chinensis* 'Old Blush' (v2); AT, *Arabidopsis thaliana*.

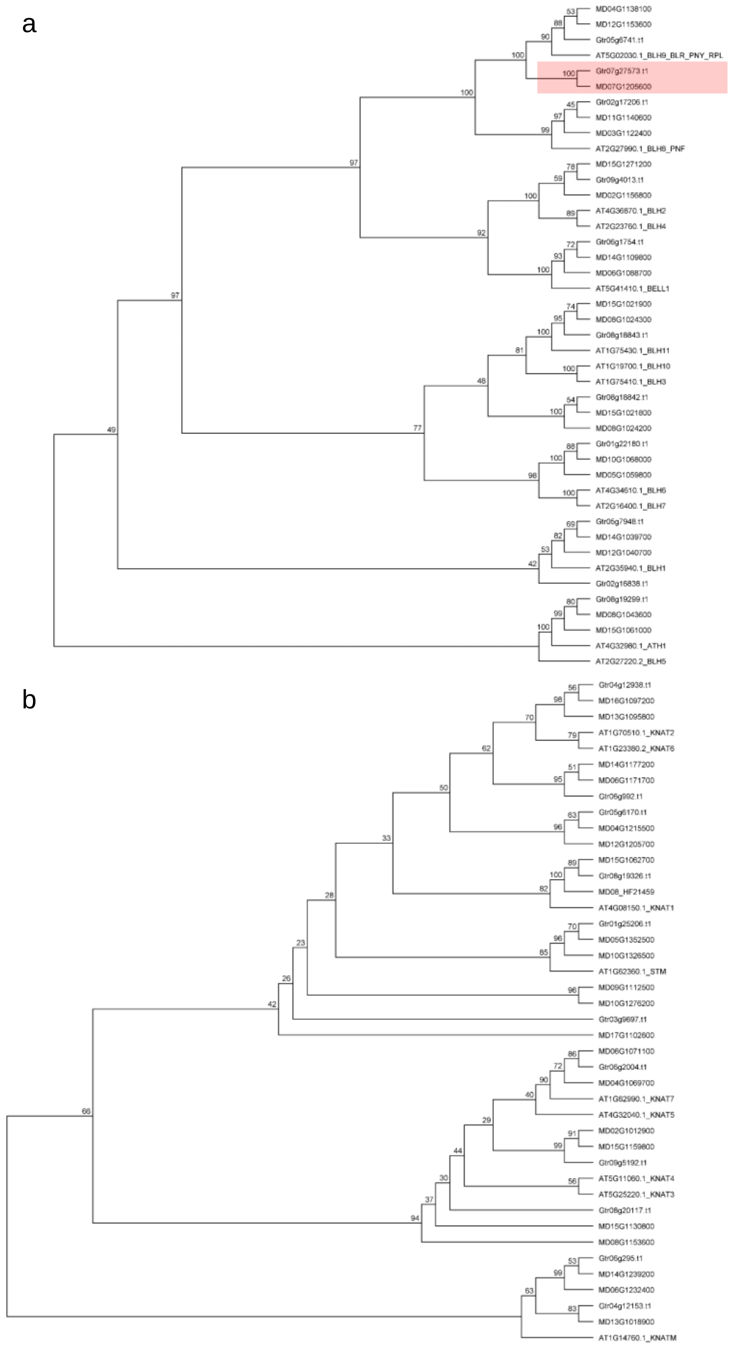

Supplementary Figure 19. Phylogenetic trees for TALE transcription factors

Phylogenetic tree of a) *BEL* transcription factors and b) *KNOX*-like transcription factors in *Gillenia*, apple and *Arabidopsis*. Pink shaded box highlights *BELLRINGER*-related clade with 1:1 ratio found to be conserved amongst Maleae species. Maximum likelihood bootstrap values from 100 datasets shown at branches. Prefixes: Gtr, *Gillenia trifoliata*; MDXXG, *Malus* GDDH13; MDXX_HF, *Malus* HFTH1; AT, *Arabidopsis thaliana*.

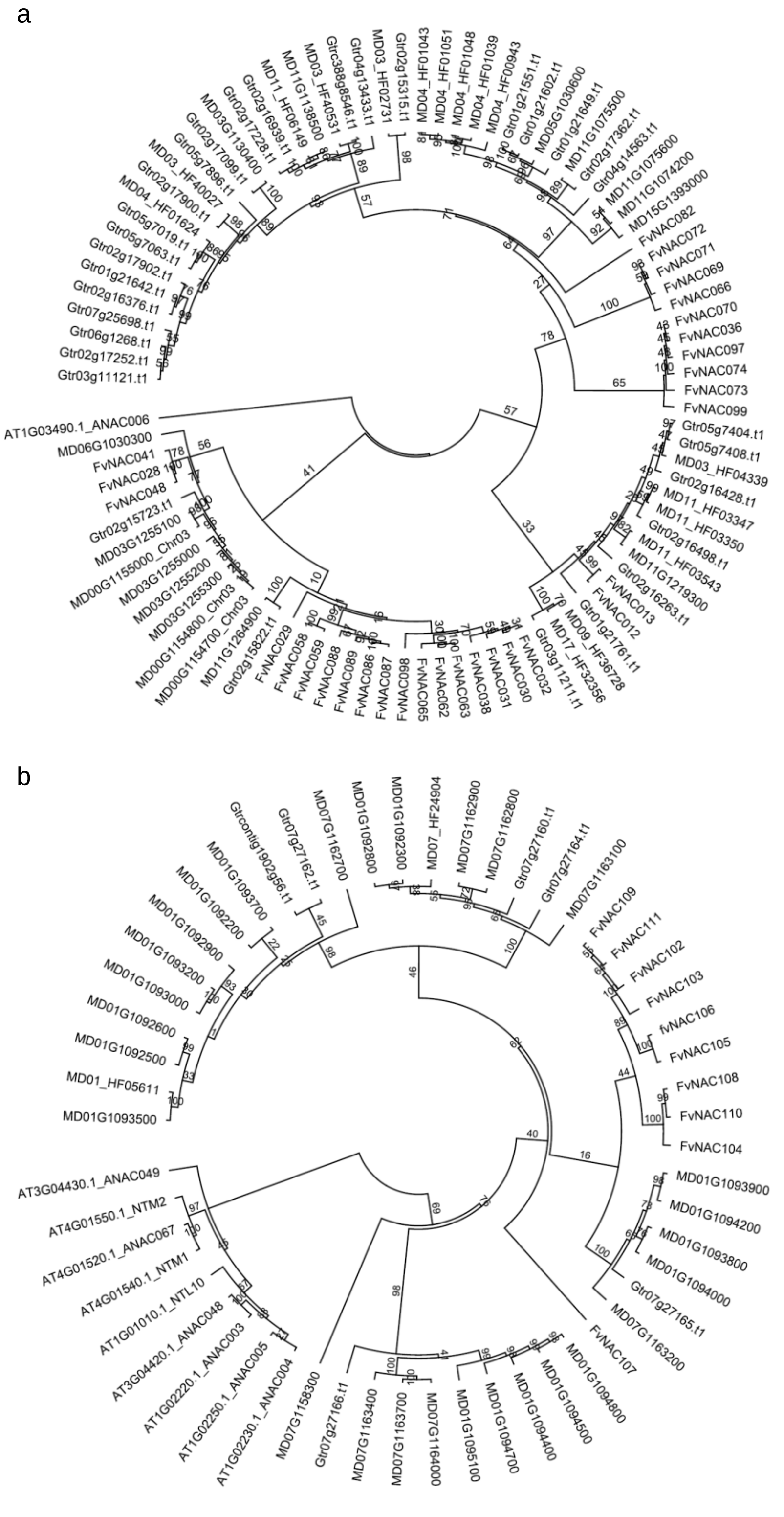

Supplementary Figure 20. Phylogenetic trees for NAC transcription factor clades of interest

Phylogenetic tree of a) putatively Rosaceae specific NAC clade and b) *ANAC1* clade with tandem gene array from *Gillenia*, *Malus*, *Fragaria* and *Arabidopsis*. Maximum likelihood bootstrap values from 100 datasets shown at branches. Prefixes: Gtr, *Gillenia trifoliata*; MDXXG, *Malus* GDDH13; MDXX_HF, *Malus* HFTH1; *Fv*, *Fragaria vesca* NACs [7]; AT, *Arabidopsis thaliana*.

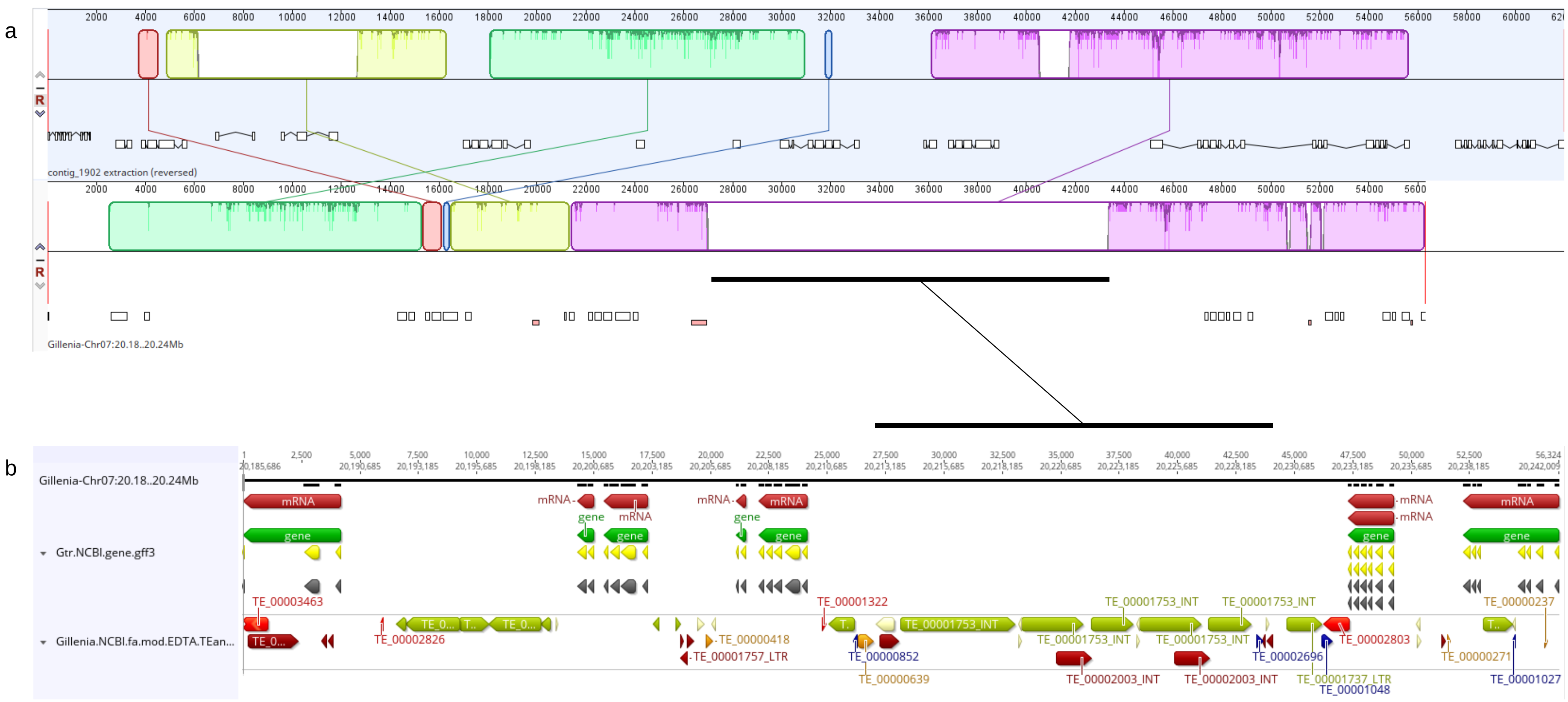

Supplementary Figure 21. ***Gillenia ANAC1*** **locus**

a) Progressive mauve alignment of *Gillenia ANAC1* locus in genome assembly with error-corrected and re-annotated *ANAC1*-associated ONT contig showing assembly rearrangements. b) The region present in the genome assembly but absent in the ONT contig is associated with a region of high transposable element density.

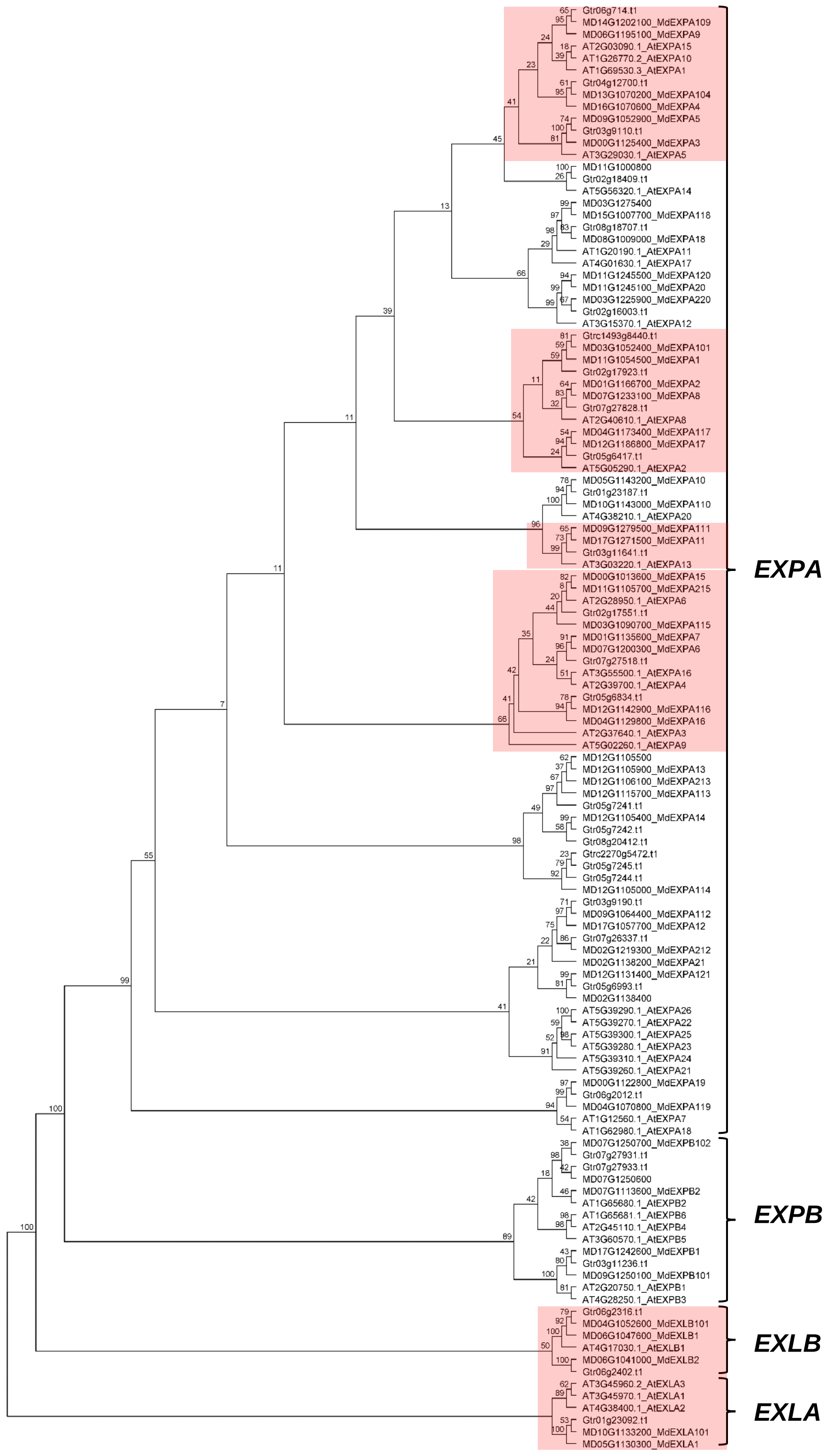

Supplementary Figure 22. Phylogenetic tree of expansins

Phylogenetic tree of cell-wall lossening expansin proteins from *Gillenia*, *Malus*, and *Arabidopsis* expansins. Subfamily clades and clades related to ripening in apple and pear as identified by [8] highlighted in light red shading. Maximum likelihood bootstrap values from 100 datasets shown at branches. Prefixes: Gtr, *Gillenia trifoliata*; MDXXG, *Malus* GDDH13; MDXX_HF, *Malus* HFTH1; AT, *Arabidopsis thaliana*.

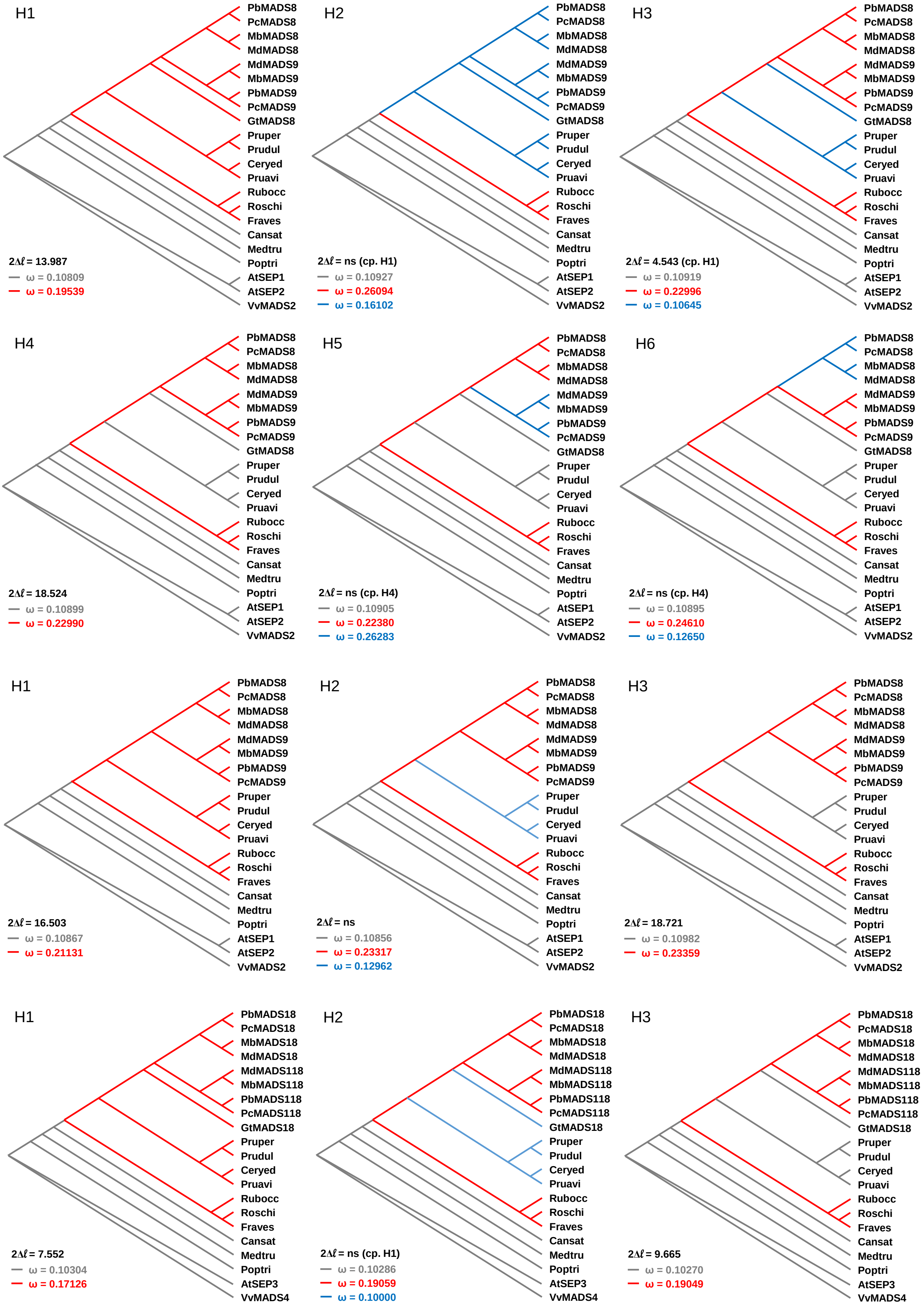

H1
PbMADS8
PcMADS8
MbMADS8
MdMADS8
MdMADS9
MbMADS9
PbMADS9
PcMADS9
GtMADS8
Pruper
Prudul
Ceryed
Pruavi
Rubocc
Roschi
Fraves
Cansat
Medtru
Poptri
AtSEP1
AtSEP2
VvMADS2
2Δℓ = 13.987
ω = 0.10809
ω = 0.19539
H2
2Δℓ = ns (cp. H1)
ω = 0.10927
ω = 0.26094
ω = 0.16102
H3
2Δℓ = 4.543 (cp. H1)
ω = 0.10919
ω = 0.22996
ω = 0.10645
H4
2Δℓ = 18.524
ω = 0.10899
ω = 0.22990
H5
2Δℓ = ns (cp. H4)
ω = 0.10905
ω = 0.22380
ω = 0.26283
H6
2Δℓ = ns (cp. H4)
ω = 0.10895
ω = 0.24610
ω = 0.12650
H1
2Δℓ = 16.503
ω = 0.10867
ω = 0.21131
H2
2Δℓ = ns
ω = 0.10856
ω = 0.23317
ω = 0.12962
H3
2Δℓ = 18.721
ω = 0.10982
ω = 0.23359
H1
PbMADS18
PcMADS18
MbMADS18
MdMADS18
MdMADS118
MbMADS118
PbMADS118
PcMADS118
GtMADS18
AtSEP3
VvMADS4
2Δℓ = 7.552
ω = 0.10304
ω = 0.17126
H2
2Δℓ = ns (cp. H1)
ω = 0.10286
ω = 0.19059
ω = 0.10000
H3
2Δℓ = 9.665
ω = 0.10270
ω = 0.19049

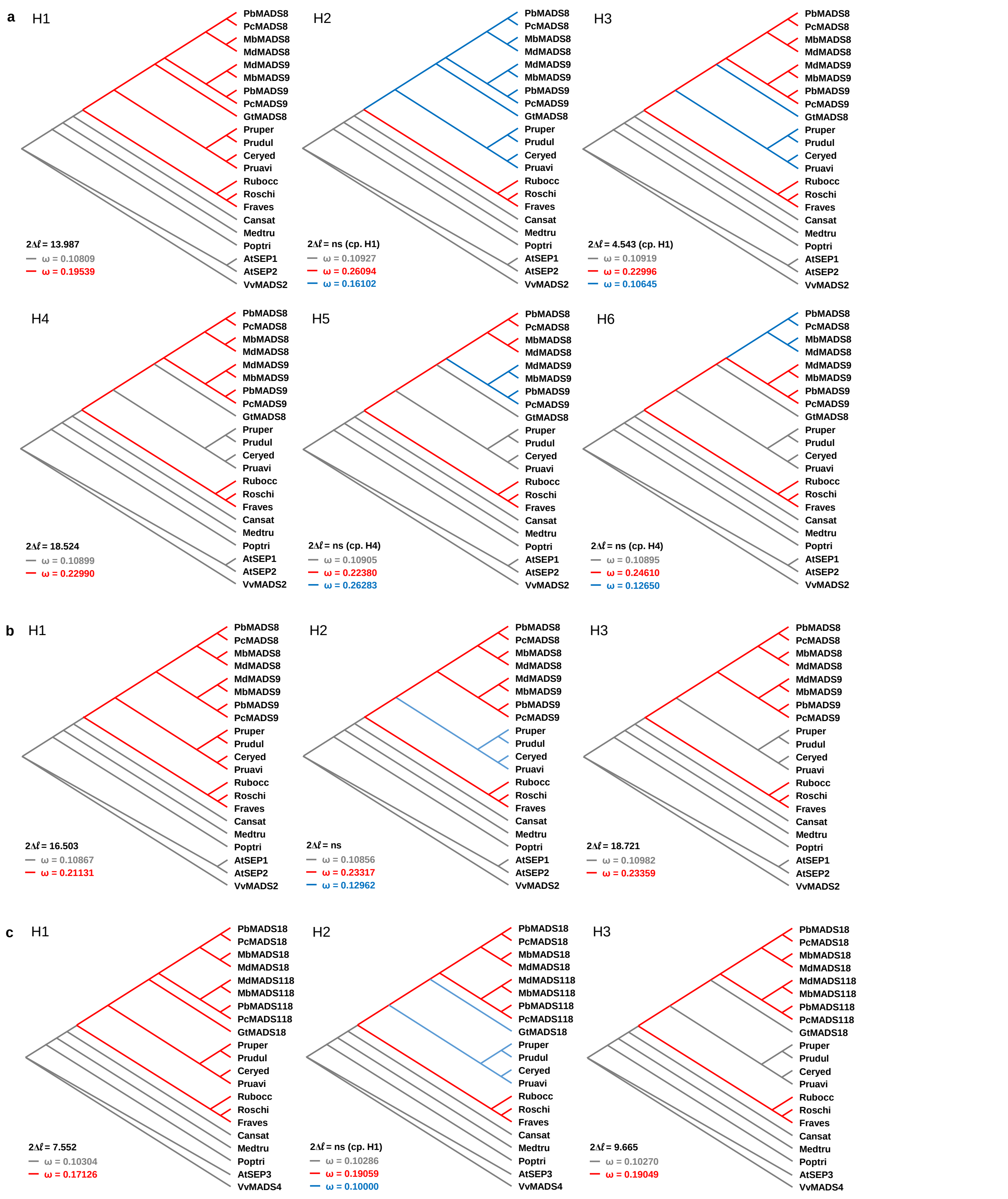

Supplementary Figure 23. Cladograms of branch-wise hypotheses testing variable dN/dS ratios

Phylogeny-based hypotheses testing the ratio of nonsynonymous to synonymous substitution rates (dN/dS, ω) in Rosid orthologous genes. a) Hypotheses of *SEP1/2*-like *MdMADS8* including *Gillenia*, b) Hypotheses of *SEP1/2*-like *MdMADS8* excluding *Gillenia*, c) Hypotheses of *SEP3*-like *MdMADS18*. 2Δl, Likelihood ratio test, measured for significance against Chi-squared distribution with 1 degree of freedom and p-value cut-off of 0.05 ($X^2_{(df=1,a=0.05)}$=3.841). Hypothesis ID's correspond to data presented in Supplementary Table 12.

Supplementary Tables

Supplementary Table 1. Shoot and reproductive organ level BBCH scales for *Gillenia trifoliata*

**Principle growth stage 0: Bud development**	
CODE	SHOOT-LEVEL DESCRIPTION
000	Dormancy and resting period: rhizome buds closed and covered by brown bud scales
001	Beginning of rhizome bud swelling: buds begin to swell and redden
003	End of rhizome bud swelling: buds swollen and red
007	Beginning of bud break: bud tips elongate and separate
009	Green to red-orange leaf tips visible
**Principle growth stage 1: Leaf development (main shoot, 100-109; lateral shoots, 119-199)**	
CODE	SHOOT-LEVEL DESCRIPTION
100	First leaves separated
101	First leaves unfolded
109	Leaves at all nodes unfolded
111	First leaves unfolded on lateral shoot, first node
119	Leaves at all nodes unfolded on lateral shoot, first node
191	First leaves unfolded on lateral shoot, ninth node
199	Leaves at all nodes unfolded on lateral shoot, ninth node
**Principle growth stage 3: Shoot development (main shoot, 300-309; lateral shoots, 319-399)**	
CODE	SHOOT-LEVEL DESCRIPTION
300	Internodes begin to elongate
301	First node detected
305	50% total shoot height reached
309	90% total shoot height reached
311	First node detected on lateral shoot, first node
319	90% total shoot length reached on lateral shoot, first node
391	First node detected on lateral shoot, ninth node
399	90% total shoot length reached on lateral shoot, ninth node
**Principle growth stage 2: Lateral shoot development on main shoot**	
CODE	SHOOT-LEVEL DESCRIPTION
200	First lateral shoot visible
209	First lateral shoot reached 90% of total length
290	Tenth lateral shoot visible
299	Tenth lateral shoot reached 90% of total length

**Principle growth stage 5: Inflorescence emergence (main shoot, 500-509; lateral shoots, 510-599)**			
CODE	SHOOT-LEVEL DESCRIPTION	CODE	ORGAN-LEVEL DESCRIPTION
501	Shoot architecture shift: terminal feathery leaves	51	Primordial inflorescence enclosed by feathery leaves
505	50% flowers visible and separated (50% ≥stage 55)	55	Green bud, pointed oval, hypanthium 1-2 mm diameter
506	60% flowers enlarged with petal tips visible	56	Bud 2-3 mm diameter, petals tips emerged
507	70% flowers enlarged with long petal tube	57	Bud 3-4 mm diameter, petals form long pointed tube
509	Most flowers with full petal tube (90% ≥stage 59)	59	Petal tube broadening and unfurling
511	Terminal node with feathery leaves on lateral shoot, first node		
519	Most flowers with petals forming broad tube visible on lateral shoot, first node		
591	Terminal node with feathery leaves on lateral shoot, ninth node		
599	Most flowers with petals forming broad tube visible on lateral shoot, ninth node		

Supplementary Table 1 continued. Shoot and reproductive organ level BBCH scales for *Gillenia*

**Principle growth stage 6: Flowering (main shoot, 600-609; lateral shoots, 610-699)**			
Code	Shoot-Level Description	Code	Organ-Level Description
600	First flowers petals parting (sporadic)	60	Petals parting, hypanthium opening constricted
603	30% flowers opening (30% ≥stage 63)	63	Petals <90° to hypanthium, stamens slightly revealed
605	Full flowering: 50% flowers open (50% ≥stage 65)	65	Petals ~90° to hypanthium, stamens light, well-revealed
607	Flowers fading: 70% flowers petals fading	67	Petals fading, >90° to hypanthium. stamens darkening
609	Most petals fallen or easily displaced	69	Petals fallen or easily displaced
615	50% flowers open on lateral shoot, first node		
619	Most petals fallen on lateral shoot, first node		
695	50% flowers open on lateral shoot, ninth node		
699	Most petals fallen on lateral shoot, ninth node		
**Principle growth stage 7: Fruit development (main shoot, 700-709; lateral shoots, 710-799)**			
Code	Shoot-Level Description	Code	Organ-Level Description
700	First fruit set (sporadic)	70	Date of pollination of stage 65 flower; 0 DAP
701	10% of receptacles enlarged	71	Receptacle enlarged (cp. stage 65); ~10 DAP
702	20% of follicles enlarged	72	Follicle length final size; ~21 DAP
703	30% of follicles bear seeds with 50% yellow coat	73	Seeds 50% yellow; ~28 DAP
704	40% of follicles bear seeds with yellow coat	74	Seeds 100% yellow
705	50% of follicles bear seeds with orange coat	75	Seeds show beginning of red pigmentation
709	90% of follicles bear seeds with burnt-orange coat	79	Most of seed coat dark red-brown colour, ~100 DAP
712	20% of follicles enlarged on lateral shoot, first node		
719	90% of follicles bear seeds with burnt-orange seed coat on lateral shoot, first node		
792	20% of follicles enlarged on lateral shoot, ninth node		
799	90% of follicles bear seeds with burnt-orange seed coat on lateral shoot, ninth node		
**PGS8**	**Fruit senescence (main shoot, 800-809; lateral shoots, 810-899)**		
Code	Shoot-Level Description	Code	Organ-Level Description
800	First fruits mature (sporadic)	80	Mature fruit, seed coat red-brown; 120 DAP
801	10% of follicles splitting under gentle force	81	Follicles splitting under gentle force
805	50% of follicles dehiscent at apex	85	Follicles dehiscent at apex
807	70% of follicles dehiscent ventrally and at apex	87	Follicles dehiscent ventrally and dorsally at apex
809	90% of follicles dehisced	89	Follicles deep brown, dehisced, seeds easily released
815	50% of follicles dehiscent at apex on lateral shoot, first node		
819	90% of follicles dehisced on lateral shoot, first node		
895	50% of follicles dehiscent at apex on lateral shoot, ninth node		
899	90% of follicles dehisced on lateral shoot, ninth node		
**PGS9**	**Vegetative senescence**		
Code	Shoot-Level description		
901	Beginning of senescence: distal edges of leaves brown; most leaves still green.		
909	End of senescence: Leaves easily detach from shoots, shoots remain attached unless manually removed		

Supplementary Table 2. Summary of correlative growth stages for *Gillenia trifoliata*

Stage	Description	Correlative stage	Correlative stage description
305	50% shoot height reached (primary shoots)	201	First lateral shoot visible (primary shoots)
		501	Shift in plant architecture upon inflorescence emergence
307*	70% shoot height reached (secondary shoots)	600	Beginning of flowering (primary shoots)
309	90% shoot height reached (primary shoots)	109	Leaves at all nodes unfolded (primary shoots)
		900	Beginning of senescence
699	End of flowering	900	Beginning of senescence

*correlation between shoots on the same rhizome

Supplementary Table 3: Flow cytometry DAPI peak data

Flow cytometry DAPI peak data with genome size estimate for *Gillenia trifoliata* relative to *Malus* x *domestica* genome size [9].

Sample	Reference	Rep	Sample peak	Reference peak	Sample:Ref ratio	Sample Genome Size (Mb)
Malus x domestica	*Trifolium repens*	1	108	192	0.56	708.54†
		2	186	335	0.56	
Gillenia trifoliata	*Trifolium repens*	1	81	330	0.25	322.6
		2	86	335	0.26	
Malus x domestica	*Bellis perennis*	1	134	317	0.42	708.54†
		2	130	312	0.42	
Gillenia trifoliata	*Bellis perennis*	1	68	352	0.19	321.6
		2	66	350	0.19	

† Estimated genome size from Zhang et al., 2019, Hanfu Triple Haploid (HFTH1) genome

Supplementary Table 4: Pseudo-chromosome assembly metrics for *Gillenia trifoliata*

Pseudo-chromosomes	No. of scaffolds	Length (bp)
Chr01	22	46,305,054
Chr02	36	32,680,979
Chr03	29	27,719,576
Chr04	30	33,455,105
Chr05	39	29,393,837
Chr06	13	26,610,424
Chr07	33	30,586,256
Chr08	19	23,036,381
Chr09	17	22,301,293
Total anchored	238	272,088,905
Total unanchored	1,253	24,214,645
Total size		296,303,550

Supplementary Table 5: Coverage of transposable elements by class for *Gillenia* genome

Class		bpMasked	%masked
LTR	Copia	28,016,728	9.46%
	Gypsy	18,790,187	6.34%
	unknow n	16,518,883	5.57%
TIR	CACTA	7,619,812	2.57%
	Mutator	19,618,949	6.62%
	PIF_Harbinger	13,544,712	4.57%
	Tc1_Mariner	518,673	0.18%
	hAT	15,208,609	5.13%
nonLTR	LINE_element	282,919	0.10%
	unknow n	272,429	0.09%
nonTIR	helitron	9,553,332	3.22%
repeat_region		9,886,172	3.34%
Total TE (bp):		139,831,405	47.19%
Total length (bp):		296,303,550	
Total sequences:		1262	

Supplementary Table 6: Coverage of transposable elements by chromosome for *Gillenia* genome

Chromosome	bpMasked	SeqLength	%masked
Chr01	24,618,222	46,305,054	53.17%
Chr02	14,368,433	32,680,979	43.97%
Chr03	11,453,164	27,719,576	41.32%
Chr04	15,644,628	33,455,105	46.76%
Chr05	14,040,868	29,393,837	47.77%
Chr06	11,802,215	26,610,424	44.35%
Chr07	13,061,198	30,586,256	42.70%
Chr08	9,024,181	23,036,381	39.17%
Chr09	9,719,016	22,301,293	43.58%
Unanchored	16,099,480	24,214,645	66.49%
Total TE (bp):	139,831,405	296,303,550	47.19%

Supplementary Table 7: Annotated transposable elements by class per *Gillenia* chromosome

Class		Chr01	Chr02	Chr03	Chr04	Chr05	Chr06	Chr07	Chr08	Chr09	Chr00
LTR											
	Copia	4,207	3331	2504	3020	2707	2411	2874	1855	1933	3360
	Gypsy	3,384	1483	1225	2143	1733	1323	1569	925	1313	2119
	unknow n	4,292	1970	1615	2451	2395	1723	2113	1249	1493	2957
TIR											
	CACTA	3,001	2436	2028	2274	2330	1988	1916	1538	1433	1685
	Mutator	10,010	7718	6537	7743	6657	6159	7076	5565	4977	3850
	PIF_Harbinger	5,310	3767	3236	3858	3296	2945	3388	2527	2435	2203
	Tc1_Mariner	331	281	256	257	223	227	282	187	190	135
	hAT	4,786	3093	2645	3438	2784	2655	2948	2140	2224	2068
nonLTR											
	LINE_element	101	125	91	92	100	80	84	61	45	54
	unknow n	41	36	44	38	32	33	41	21	8	27
nonTIR											
	helitron	5,360	4187	3311	3998	3485	3075	3447	2832	2627	2282
long_terminal_repeat		332	230	146	246	182	168	148	136	134	70
repeat_region		5,546	4417	3647	3791	3631	3245	4023	2615	2769	3102
target_site_duplication		330	230	146	246	180	168	148	136	134	70
TE count		47,031	33,304	27,431	33,595	29,735	26,200	30,057	21,787	21,715	23,982
Chr length (Mb)		46.31	32.68	27.72	33.46	29.39	26.61	30.59	23.04	22.30	24.56
TE count per kb		1.02	1.02	0.99	1.00	1.01	0.98	0.98	0.95	0.97	0.98

Supplementary Table 8: Transfer RNA by chromosome and unanchored contigs for *Gillenia*

Sequence name	tRNA count	sequence length (bp)
Chr01	57	46,305,054
Chr02	55	32,680,979
Chr03	52	27,719,576
Chr04	54	33,455,105
Chr05	48	29,393,857
Chr06	49	26,610,424
Chr07	39	30,586,256
Chr08	49	23,036,381
Chr09	41	22,301,293
contig_1706_RaGOO	20	118,019
contig_311_RaGOO	1	6,327
contig_1517_RaGOO	7	47,051
S4.1.27149_RaGOO	1	7,693
contig_2209_RaGOO	3	23,488
contig_2348_RaGOO	8	114,879
contig_731_RaGOO	1	15,778
contig_2384_RaGOO	8	133,003
contig_497_RaGOO	4	83,654
contig_3091_RaGOO	2	47,745
contig_2427_RaGOO	3	91,227
contig_2045_RaGOO	1	34,570
contig_537_RaGOO	1	44,486
contig_270_RaGOO	1	44,717
contig_575_RaGOO	1	63,621
contig_757_RaGOO	1	76,727
contig_2774_RaGOO	1	77,043
contig_48_RaGOO	1	93,284
contig_131_RaGOO	1	110,320
contig_156_RaGOO	2	299,447
contig_198_RaGOO	1	163,254
contig_952_RaGOO	1	255,874

Supplementary Table 9: GO enrichment for orthologous clusters of interest between *Gillenia*, *Malus*, *Pyrus*, *Prunus*, *Fragaria* and *Rubus*.

GO ID	Namespace	Name	Cluster Count	p-value
All (13356 clusters)				
GO:0015074	biological_process	DNA integration	2	1.57E-36
GO:0007165	biological_process	signal transduction	44	1.40E-16
GO:0006952	biological_process	defense response	85	7.18E-14
GO:0016705	molecular_function	oxidoreductase activity, acting on paired donors, w ith incorporation or reduction of molecular oxygen	27	3.21E-11
GO:0009820	biological_process	alkaloid metabolic process	7	4.01E-07
GO:0005886	cellular_component	plasma membrane	87	8.95E-07
GO:0004523	molecular_function	RNA-DNA hybrid ribonuclease activity	2	4.53E-06
GO:0050832	biological_process	defense response to fungus	11	7.66E-06
GO:0005524	molecular_function	ATP binding	13	9.87E-06
GO:0006412	biological_process	translation	134	2.24E-05
GO:0035251	molecular_function	UDP-glucosyltransferase activity	2	2.85E-05
GO:0008284	biological_process	positive regulation of cell proliferation	3	3.65E-05
GO:0016114	biological_process	terpenoid biosynthetic process	6	4.40E-05
GO:0009734	biological_process	auxin-activated signaling pathw ay	12	6.48E-05
GO:0004674	molecular_function	protein serine/threonine kinase activity	70	8.15E-05
GO:0009451	biological_process	RNA modification	181	0.000125394
GO:0016491	molecular_function	oxidoreductase activity	50	0.000143861
GO:0008270	molecular_function	zinc ion binding	49	0.000223489
GO:0006355	biological_process	regulation of transcription, DNA-templated	305	0.000519556
GO:0009611	biological_process	response to w ounding	85	0.000569992
Amygdaloideae only (593 clusters)				
GO:0009451	biological_process	RNA modification	18	9.23E-05
GO:0052747	molecular_function	sinapyl alcohol dehydrogenase activity	2	0.000480851
Rosoideae only (993 clusters)				
GO:0046274	biological_process	lignin catabolic process	7	1.61E-06
GO:0003677	molecular_function	DNA binding	12	1.23E-05
GO:0010016	biological_process	shoot system morphogenesis	4	4.80E-05
GO:0016705	molecular_function	oxidoreductase activity, acting on paired donors, w ith ir	12	7.32E-05
GO:0006511	biological_process	ubiquitin-dependent protein catabolic process	18	0.000121107
GO:0019748	biological_process	secondary metabolic process	5	0.000233902
GO:0006334	biological_process	nucleosome assembly	5	0.000423127
GO:0010951	biological_process	negative regulation of endopeptidase activity	4	0.000444883
Malodae only (549 clusters)				
GO:0008194	molecular_function	UDP-glycosyltransferase activity	7	5.87E-05
GO:0045944	biological_process	positive regulation of transcription from RNA polymerase II promoter	7	0.000149945
GO:0008168	molecular_function	methyltransferase activity	7	0.000356953
All except Malodae (172 clusters)				
GO:0005216	molecular_function	ion channel activity	4	0.000203153
GO:0006952	biological_process	defense response	8	0.000254102
Maleae only (2943 clusters)				
GO:0009451	biological_process	RNA modification	12	6.80E-05
GO:0006952	biological_process	defense response	48	0.000137
All except Maleae (77 clusters)				
No GO enrichment				
Gillenia only (712 clusters)				
No GO enrichment				
All except Gillenia (469 clusters)				
No GO enrichment				
Gillenia and Malus only (157 clusters)				
GO:0004867	molecular_function	serine-type endopeptidase inhibitor activity	3	3.74E-07
GO:0005524	molecular_function	ATP binding	4	0.000139695
GO:0015812	biological_process	gamma-aminobutyric acid transport	2	0.000905481
Gillenia and Pyrus only (376 clusters)				
GO:0010223	biological_process	secondary shoot formation	3	0.000120848
GO:0005739	cellular_component	mitochondrion	6	0.000803645
Gillenia and Prunus only (124 clusters)				
GO:0006952	biological_process	defense response	7	5.17E-05
GO:0010038	biological_process	response to metal ion	2	0.000712651
GO:0051707	biological_process	response to other organism	2	0.000839466
Gillenia and Fragaria only (78 clusters)				
No GO enrichment				
Gillenia and Rubus only (49 clusters)				
GO:0006952	biological_process	defense response	2	0.024095025

Supplementary Table 10: Conversion table of TALE transcription factor MDP models

Conversion of MDP models identified by Li et al [10] to GDDH13 or HFTH1 models identified in this study.

TALE subfamily	Li et al 2019 name	MDP number	best GDDH/HFTH model	Note
KNOX	MdHOX10	MDP0000323095	MD02G1012900	multiple MDP-model locus; alternate MDP model to MDP0000137501
KNOX	MdHOX11	MDP0000136205	MD02G1012900	multiple MDP-model locus; alternate MDP model to MDP0000137501
KNOX	MdHOX12	MDP0000137501	MD02G1012900	multiple MDP-model locus
KNOX	MdHOX43	MDP0000244941	MD04G1215500	
KNOX	MdHOX44	MDP0000231216	MD05G1352500	
KNOX	MdHOX59	MDP0000313776	MD06G1071100	
KNOX	MdHOX63	MDP0000254187	MD06G1171700	
KNOX	MdHOX83	MDP0000280307	HF21459-RA	
KNOX	MdHOX89	MDP0000135063	MD08G1153600	
KNOX	MdHOX100	MDP0000222097	MD09G1112500	
KNOX	MdHOX108	MDP0000551496	MD04G1069700	
KNOX	MdHOX113	MDP0000285490	MD10G1276200	
KNOX	MdHOX117	MDP0000136226	MD10G1326500	
KNOX	MdHOX136	MDP0000131500	MD12G1205700	multiple MDP-model locus
KNOX	MdHOX137	MDP0000187050	MD12G1205700	multiple MDP-model locus; alternate MDP model to MDP0000131500
KNOX	MdHOX141	MDP0000252095	MD13G1095800	
KNOX	MdHOX159	MDP0000254847	MD14G1177200	
KNOX	MdHOX166	MDP0000599531	MD15G1062700	multiple MDP-model locus; alternate MDP model to MDP0000722139
KNOX	MdHOX167	MDP0000722139	MD15G1062700	multiple MDP-model locus
KNOX	MdHOX174	MDP0000167286	MD15G1130800	
KNOX	MdHOX175	MDP0000149322	MD15G1159800	
KNOX	MdHOX190	MDP0000294096	MD16G1097200	
KNOX	MdHOX197	MDP0000233192	MD17G1102600	
KNOX-KNATM	not identified	MDP0000172375	MD13G1018900	
KNOX-KNATM	not identified	MDP0000242411	MD06G1232400	
KNOX-KNATM	not identified	MDP0000299540	MD14G1239200	
BEL	MdHOX1	MDP0000143173	MD14G1109800	
BEL	MdHOX20	MDP0000130825	MD02G1156800	
BEL	MdHOX30	MDP0000255392	MD03G1122400	multiple MDP-model locus; alternate MDP model to MDP0000596661
BEL	MdHOX31	MDP0000596661	MD03G1122400	multiple MDP-model locus
BEL	MdHOX32	MDP0000429775	MD04G1138100	multiple MDP-model locus
BEL	MdHOX33	MDP0000126587	MD04G1138100	multiple MDP-model locus; alternate MDP model to MDP0000429775
BEL	MdHOX75	MDP0000204889	MD08G1024200	
BEL	MdHOX76	MDP0000204890	MD08G1024300	multiple MDP-model locus
BEL	MdHOX77	MDP0000283117	MD08G1024300	multiple MDP-model locus; alternate MDP model to MDP0000204890
BEL	MdHOX78	MDP0000248279	MD05G1059800	
BEL	MdHOX81	MDP0000166587	MD08G1043600	
BEL	MdHOX110	MDP0000239684	MD10G1068000	
BEL	MdHOX122	MDP0000272542	MD11G1140600	
BEL	MdHOX127	MDP0000128214	MD12G1040700	
BEL	MdHOX132	MDP0000304106	MD15G1021900	
BEL	MdHOX133	MDP0000210119	MD15G1021800	
BEL	MdHOX135	MDP0000209399	MD12G1153600	
BEL	MdHOX147	MDP0000125413	MD07G1205600	
BEL	MdHOX149	MDP0000228381	MD14G1039700	multiple MDP-model locus; alternate MDP model to MDP0000841379
BEL	MdHOX150	MDP0000841379	MD14G1039700	multiple MDP-model locus
BEL	MdHOX151	MDP0000546502	MD14G1039700	multiple MDP-model locus; alternate MDP model to MDP0000841379
BEL	MdHOX165	MDP0000300503	MD15G1061000	
BEL	MdHOX179	MDP0000141097	MD15G1271200	
BEL	not identified	MDP0000123680	MD06G1088700	

Supplementary Table 11: Conversion table of NAC transcription factor MDP models

Conversion of MDP models identified in Su et al [11] and Busatto et al [12] to GDDH13 or HFTH1 models identified in this study.

Source	Name	MDP number	best GDDH/HFTH model	Note
Su et al 2013	MdNAC1	MDP0000882983	MD01G1092200	
Su et al 2013	MdNAC2	MDP0000836293	MD01G1092500	
Su et al 2013	MdNAC3	MDP0000136652	MD01G1093000	multiple MDP model locus; alternate model to MDP-182344
Su et al 2013	MdNAC4	MDP0000182344	MD01G1093000	multiple MDP model locus; alternate model to MDP-136652
Su et al 2013	MdNAC5	MDP0000392668	MD01G1093200	multiple MDP model locus; alternate model to MDP-291838
Su et al 2013	MdNAC6	MDP0000688878	MD01G1093500	multiple MDP model locus; alternate model to MDP-138340
Su et al 2013	MdNAC7	MDP0000291838	MD01G1093200	multiple MDP model locus; alternate model to MDP-392668
Su et al 2013	MdNAC8	MDP0000138340	MD01G1093500	multiple MDP model locus; alternate model to MDP-688878
Su et al 2013	MdNAC9	MDP0000200646	MD01G1093700	multiple MDP model locus; alternate model to MDP-198108
Su et al 2013	MdNAC10	MDP0000279197	MD01G1093800	multiple MDP model locus; alternate model to MDP-280486
Su et al 2013	MdNAC11	MDP0000280486	MD01G1093800	multiple MDP model locus; alternate model to MDP-279197
Su et al 2013	MdNAC12	MDP0000440829	MD01G1094000	
Su et al 2013	MdNAC13	MDP0000183558	MD01G1094500	
Su et al 2013	MdNAC14	MDP0000131126	MD00G1117000	multiple MDP model locus; alternate model to MDP-221977
Su et al 2013	MdNAC15	MDP0000221977	MD00G1117000	multiple MDP model locus; alternate model to MDP-131126
Su et al 2013	MdNAC16	MDP0000124509	MD02G1068700	
Su et al 2013	MdNAC17	MDP0000180605	MD02G1243800	
Su et al 2013	MdNAC18	MDP0000437676	MD02G1243900	
Su et al 2013	MdNAC19	MDP0000267678	HF40027-RA	multiple MDP model locus; alternate model to MDP-921016
Su et al 2013	MdNAC20	MDP0000921016	HF40027-RA	multiple MDP model locus; alternate model to MDP-267678/-267681
Su et al 2013	MdNAC21	MDP0000267681	HF40027-RA	multiple MDP model locus; alternate model to MDP-921016
Su et al 2013	MdNAC22	MDP0000499023	no GDDH13 and HFTH1 hits	may be duplicate of MDP-516159
Su et al 2013	MdNAC23	MDP0000516159	MD03G1115100	
Su et al 2013	MdNAC24	MDP0000595295	MD03G1130400	
Su et al 2013	MdNAC25	MDP0000759504	MD03G1148500	multiple MDP model locus; alternate model to MDP-130797
Su et al 2013	MdNAC26	MDP0000130797	MD03G1148500	multiple MDP model locus; alternate model to MDP-759504
Su et al 2013	MdNAC27	MDP0000133636	MD03G1150800	
Su et al 2013	MdNAC28	MDP0000299944	MD03G1172900	multiple MDP model locus; alternate model to MDP-132627
Su et al 2013	MdNAC29	MDP0000132627	MD03G1172900	multiple MDP model locus; alternate model to MDP-299944
Su et al 2013	MdNAC30	MDP0000180343	HF04202-RA	no GDDH model
Su et al 2013	MdNAC31	MDP0000315947	HF04339-RA	no GDDH model
Su et al 2013	MdNAC32	MDP0000868419	MD03G1222600	
Su et al 2013	MdNAC33	MDP0000262032	MD03G1222700	
Su et al 2013	MdNAC34	MDP0000135860	HF03247-RA	no GDDH model
Su et al 2013	MdNAC35	MDP0000215351	MD03G1255000	multiple MDP model locus; tandem duplication of near identical models
Su et al 2013	MdNAC36	MDP0000222045	MD03G1255300	
Su et al 2013	MdNAC37	MDP0000907178	MD00G1154700	
Su et al 2013	MdNAC38	MDP0000174734	MD00G1154800	
Su et al 2013	MdNAC39	MDP0000307020	no GDDH13 and HFTH1 hits	
Su et al 2013	MdNAC40	MDP0000256806	HF01624-RA	no GDDH model
Su et al 2013	MdNAC41	MDP0000802924	MD04G1100400	
Su et al 2013	MdNAC42	MDP0000173636	HF12113-RA	best GDDH hits are split models MD05G1321900-MD05G1321800
Su et al 2013	MdNAC43	MDP0000276278	MD05G1309700	
Su et al 2013	MdNAC44	MDP0000460430	MD00G1085300	
Su et al 2013	MdNAC45	MDP0000199840	MD05G1239300	
Su et al 2013	MdNAC46	MDP0000800550	MD05G1030600	
Su et al 2013	MdNAC47	MDP0000136180	MD15G1247800	multiple MDP model locus; alternate model to MDP-299959
Su et al 2013	MdNAC48	MDP0000299959	MD15G1247800	multiple MDP model locus; alternate model to MDP-298965/-136180
Su et al 2013	MdNAC49	MDP0000298965	MD15G1247800	multiple MDP model locus; alternate model to MDP-299959
Su et al 2013	MdNAC50	MDP0000257674	HF31758-RA	MD05G1005600 model for this locus is partial model
Su et al 2013	MdNAC51	MDP0000167208	MD06G1004900	multiple MDP model locus; alternate model to MDP-191344
Su et al 2013	MdNAC52	MDP0000191344	MD06G1004900	multiple MDP model locus; alternate model to MDP-167208/-198235
Su et al 2013	MdNAC53	MDP0000911336	MD06G1030300	
Su et al 2013	MdNAC54	MDP0000772731	MD06G1030300	
Su et al 2013	MdNAC55	MDP0000762302	MD06G1121400	
Su et al 2013	MdNAC56	MDP0000130686	MD06G1135700	
Su et al 2013	MdNAC57	MDP0000911724	MD06G1196100	
Su et al 2013	MdNAC58	MDP0000246482	MD06G1216500	multiple MDP model locus; alternate model to MDP-153949
Su et al 2013	MdNAC59	MDP0000153949	MD06G1216500	multiple MDP model locus; alternate model to MDP-246482
Su et al 2013	MdNAC60	MDP0000387787	MD06G1236800	MD06G1236800 best representative but truncated
Su et al 2013	MdNAC61	MDP0000258167	MD07G1073100	
Su et al 2013	MdNAC62	MDP0000184706	MD03G1255000	multiple MDP model locus; tandem duplication of near identical models
Su et al 2013	MdNAC63	MDP0000563165	MD07G1158300	
Su et al 2013	MdNAC64	MDP0000249947	MD07G1162700	
Su et al 2013	MdNAC65	MDP0000231843	MD07G1163700	multiple MDP model locus; tandem duplication of near identical models
Su et al 2013	MdNAC66	MDP0000231845	MD07G1163200	
Su et al 2013	MdNAC67	MDP0000282616	MD07G1163400	
Su et al 2013	MdNAC68	MDP0000214515	MD07G1163700	multiple MDP model locus; tandem duplication of near identical models
Su et al 2013	MdNAC69	MDP0000311190	MD07G1164000	multiple MDP model locus; alternate model to MDP-575835
Su et al 2013	MdNAC70	MDP0000575835	MD07G1164000	multiple MDP model locus; alternate model to MDP-311190/-274989
Su et al 2013	MdNAC71	MDP0000274989	MD07G1164000	multiple MDP model locus; alternate model to MDP-575835
Su et al 2013	MdNAC72	MDP0000226701	MD07G1254800-4700	both GDDH13 and HFTH1 models are split
Su et al 2013	MdNAC73	MDP0000907489	MD07G1301800	
Su et al 2013	MdNAC74	MDP0000690168	MD08G1121300	
Su et al 2013	MdNAC75	MDP0000283092	MD01G1094200	
Su et al 2013	MdNAC76	MDP0000278387	HF05611-RA	best representative model betw een GDDH and HFTH1
Su et al 2013	MdNAC77	MDP0000198108	MD01G1093700	multiple MDP model locus; alternate model to MDP-200646
Su et al 2013	MdNAC78	MDP0000240094	MD15G1415700	
Su et al 2013	MdNAC79	MDP0000309382	MD09G1006400	
Su et al 2013	MdNAC80	MDP0000276765	MD09G1053700	
Su et al 2013	MdNAC81	MDP0000902822	MD05G1042100	
Su et al 2013	MdNAC82	MDP0000852271	MD05G1037700	
Su et al 2013	MdNAC83	MDP0000849944	MD10G1048500-8600	both GDDH13 and HFTH1 models are split
Su et al 2013	MdNAC84	MDP0000480581	MD05G1130500	
Su et al 2013	MdNAC85	MDP0000679946	MD08G1250000	
Su et al 2013	MdNAC86	MDP0000697030	HF30016-RA	no GDDH model
Su et al 2013	MdNAC87	MDP0000139773	MD10G1186100	fragment
Su et al 2013	MdNAC88	MDP0000298182	MD10G1198400	
Su et al 2013	MdNAC89	MDP0000204582	MD10G1217500	
Su et al 2013	MdNAC90	MDP0000652584	MD10G1220600	multiple MDP model locus; alternate model to MDP-501518
Su et al 2013	MdNAC91	MDP0000309351	MD10G1288300	
Su et al 2013	MdNAC92	MDP0000120881	MD10G1299900	
Su et al 2013	MdNAC93	MDP0000265416	no GDDH13 and HFTH1 hits	
Su et al 2013	MdNAC94	MDP0000382720	no GDDH13 and HFTH1 hits	
Su et al 2013	MdNAC95	MDP0000148746	MD11G1074200	multiple MDP model locus; alternate model to MDP-171771
Su et al 2013	MdNAC96	MDP0000171771	MD11G1074200	multiple MDP model locus; alternate model to MDP-148746/-394116
Su et al 2013	MdNAC97	MDP0000394116	MD11G1074200	multiple MDP model locus; alternate model to MDP-171771
Su et al 2013	MdNAC98	MDP0000289955	MD11G1075600	
Su et al 2013	MdNAC99	MDP0000246470	MD00G1146200	multiple MDP model locus; alternative model to MDP-254112
Su et al 2013	MdNAC100	MDP0000254112	MD00G1146200	multiple MDP model locus; alternative model to MDP-246470
Su et al 2013	MdNAC101	MDP0000255884	MD11G1133000	
Su et al 2013	MdNAC102	MDP0000265616	MD11G1138500	
Su et al 2013	MdNAC103	MDP0000125980	HF06149-RA	multiple MDP model locus; alternate model to MDP-125980
Su et al 2013	MdNAC104	MDP0000807984	HF06149-RA	multiple MDP model locus; alternate model to MDP-807984
Su et al 2013	MdNAC105	MDP0000206199	MD11G1219300	
Su et al 2013	MdNAC106	MDP0000206868	HF03543-RA	no GDDH model
Su et al 2013	MdNAC107	MDP0000233010	MD11G1190900	
Su et al 2013	MdNAC108	MDP0000285555	HF03347-RA	no GDDH model
Su et al 2013	MdNAC109	MDP0000213555	HF03349-RA	no GDDH model
Su et al 2013	MdNAC110	MDP0000674514	MD11G1239900	
Su et al 2013	MdNAC111	MDP0000655666	MD11G1264900	
Su et al 2013	MdNAC112	MDP0000184069	MD12G1001200	
Su et al 2013	MdNAC113	MDP0000206212	MD12G1030200	
Su et al 2013	MdNAC114	MDP0000718993	HF37866-RA	multiple MDP model locus; alternate model to MDP-634006
Su et al 2013	MdNAC115	MDP0000634006	HF37866-RA	multiple MDP model locus; alternate model to MDP-718993
Su et al 2013	MdNAC116	MDP0000310981	MD11G1253500	
Su et al 2013	MdNAC117	MDP0000165845	MD13G1046600	multiple MDP model locus; alternate model to MDP-216205
Su et al 2013	MdNAC118	MDP0000216205	MD13G1046600	multiple MDP model locus; alternate model to MDP-165845
Su et al 2013	MdNAC119	MDP0000655623	MD13G1063900	
Su et al 2013	MdNAC120	MDP0000481448	MD13G1069200	
Su et al 2013	MdNAC121	MDP0000186765	MD13G1072500	
Su et al 2013	MdNAC122	MDP0000126517	MD13G1125000	
Su et al 2013	MdNAC123	MDP0000205938	MD11G1167900	
Su et al 2013	MdNAC124	MDP0000180683	MD14G1001000	
Su et al 2013	MdNAC125	MDP0000152400	MD14G1030800	multiple MDP model locus; alternate model to MDP-252746
Su et al 2013	MdNAC126	MDP0000252746	MD14G1030800	
Su et al 2013	MdNAC127	MDP0000252435	MD14G1030700	
Su et al 2013	MdNAC128	MDP0000506233	MD14G1030900	
Su et al 2013	MdNAC129	MDP0000558493	MD10G1000900	
Su et al 2013	MdNAC130	MDP0000176447	MD02G1134500	
Su et al 2013	MdNAC131	MDP0000722092	MD14G1071200	
Su et al 2013	MdNAC132	MDP0000868556	MD10G1133400	
Su et al 2013	MdNAC133	MDP0000140229	MD14G1137900	
Su et al 2013	MdNAC134	MDP0000561788	no GDDH13 and HFTH1 hits	
Su et al 2013	MdNAC135	MDP0000587003	no GDDH13 and HFTH1 hits	
Su et al 2013	MdNAC136	MDP0000266908	MD00G1096600	
Su et al 2013	MdNAC137	MDP0000147080	MD14G1150900	
Su et al 2013	MdNAC138	MDP0000121265	MD14G1203000	
Su et al 2013	MdNAC139	MDP0000130785	MD14G1226900	
Su et al 2013	MdNAC140	MDP0000656113	MD14G1243700	
Su et al 2013	MdNAC141	MDP0000152774	MD15G1079400	
Su et al 2013	MdNAC142	MDP0000232008	MD15G1100100	
Su et al 2013	MdNAC143	MDP0000334047	MD15G1136600	
Su et al 2013	MdNAC144	MDP0000667743	MD03G1255100	tandem duplication of near identical models
Su et al 2013	MdNAC145	MDP0000272164	MD03G1255000	multiple MDP model locus; tandem duplication of near identical models
Su et al 2013	MdNAC146	MDP0000168681	MD01G1088300	
Su et al 2013	MdNAC147	MDP0000240855	MD04G1043700	
Su et al 2013	MdNAC148	MDP0000259556	HF00943-RA	no GDDH model
Su et al 2013	MdNAC149	MDP0000618650	MD15G1200200	multiple MDP model locus; alternate model to MDP-157070
Su et al 2013	MdNAC150	MDP0000157070	MD15G1200200	multiple MDP model locus; alternate model to MDP-618650
Su et al 2013	MdNAC151	MDP0000759612	MD15G1393000	multiple MDP model locus; alternate model to MDP-126009
Su et al 2013	MdNAC152	MDP0000189394	MD15G1444700	
Su et al 2013	MdNAC153	MDP0000224592	MD16G1048300	
Su et al 2013	MdNAC154	MDP0000457996	MD07G1073200	multiple MDP model locus; alternate model to MDP-695714
Su et al 2013	MdNAC155	MDP0000695714	MD07G1073200	multiple MDP model locus; alternate model to MDP-457996
Su et al 2013	MdNAC156	MDP0000236349	MD16G1062600	multiple MDP model locus; alternate model to MDP-918566
Su et al 2013	MdNAC157	MDP0000918566	MD16G1062600	multiple MDP model locus; alternate model to MDP-236349
Su et al 2013	MdNAC158	MDP0000262990	MD16G1069500	
Su et al 2013	MdNAC159	MDP0000241821	MD16G1073300	
Su et al 2013	MdNAC160	MDP0000130123	MD16G1125800	
Su et al 2013	MdNAC161	MDP0000936269	HF16354-RA	MD16G1276900-7000 are best representatives but are split models
Su et al 2013	MdNAC162	MDP0000842702	MD17G1010300	
Su et al 2013	MdNAC163	MDP0000232050	MD17G1051600	multiple MDP model locus; alternate model to MDP-129335
Su et al 2013	MdNAC164	MDP0000129335	MD17G1051600	multiple MDP model locus; alternate model to MDP-232050
Su et al 2013	MdNAC165	MDP0000404409	MD12G1000800	
Su et al 2013	MdNAC166	MDP0000520807	MD17G1286600	
Su et al 2013	MdNAC167	MDP0000501518	MD10G1220600	multiple MDP model locus; alternate model to MDP-652584
Su et al 2013	MdNAC168	MDP0000132623	HF03744-RA	no GDDH model
Su et al 2013	MdNAC169	MDP0000458799	MD08G1232800	
Su et al 2013	MdNAC170	MDP0000169485	MD10G1041500	
Su et al 2013	MdNAC171	MDP0000146969	MD10G1041500	
Su et al 2013	MdNAC172	MDP0000177041	HF01043-RA	no GDDH model
Su et al 2013	MdNAC173	MDP0000267984	HF01039-RA	no GDDH model
Su et al 2013	MdNAC174	MDP0000936441	MD14G1069400	
Su et al 2013	MdNAC175	MDP0000296292	MD03G1172900	multiple MDP model locus; alternate model to MDP-132627
Su et al 2013	MdNAC176	MDP0000219488	MD10G1041500	multiple MDP model locus; alternate model to MDP-146969
Su et al 2013	MdNAC177	MDP0000126009	MD15G1393000	multiple MDP model locus; alternate model to MDP-759612
Su et al 2013	MdNAC178	MDP0000497016	MD00G1155000	
Su et al 2013	MdNAC179	MDP0000225852	MD03G1255200	tandem duplication of near identical models
Su et al 2013	MdNAC180	MDP0000198235	MD06G1004900	multiple MDP model locus; alternate model to MDP-191344
Busatto et al 2016	NAC	MDP0000119446	MD06G1031700	

Supplementary Table 12. Branch-wise hypotheses testing dN/dS ratios

Branch-wise hypotheses testing variable nonsynonymous to synonymous substitution rates (dN/dS, ω) amongst Rosid orthologous genes. a) Hypotheses of *SEP1/2*-like *MdMADS8* including *Gillenia*, b) Hypotheses of *SEP1/2*-like *MdMADS8* excluding *Gillenia*, c) Hypotheses of *SEP3*-like *MdMADS18*. Hypothesis ID's correspond to cladograms presented in Supplementary Figure 22.

a

			average ω (dN/dS)									
ID	hypothesis description	ratio	Orthologues (O)	Rosoideae (R)	Amygdaleae (A)	Gillenia	Maleae-MADS8	Maleae-MADS9	likelihood	comparison	2Δl	p-value
H0	all equal	one	0.13	$=\omega_O$	$=\omega_O$	$=\omega_O$	$=\omega_O$	$=\omega_O$	-4850.328376	na	na	na
H1	Orthologues vs. Rosaceae	two	0.11	0.20	$=\omega_R$	$=\omega_R$	$=\omega_R$	$=\omega_R$	-4843.335081	H1-H0	13.987	0.00018
H2	Orthologues vs. Rosoideae vs. Amygdaloideae	three	0.11	0.26	0.16	$=\omega_A$	$=\omega_A$	$=\omega_A$	-484174441	H2-H1	3.181	ns
H3	Orthologues vs. FA-Rosaceae vs. non-FA-Rosaceae	three	0.11	0.23	0.11	$=\omega_A$	$=\omega_R$	$=\omega_R$	-4841063722	H3-H1	4.543	0.03305
H4	Orthologues & non-FA-Rosaceae vs. FA-Rosaceae	two	0.11	0.23	$=\omega_O$	$=\omega_O$	$=\omega_R$	$=\omega_R$	-4841066379	H4-H0	18.524	0.00002
H5	Orthologues & non-FA-Rosaceae vs. Rosoideae & Mal-MADS8 vs. Mal-MADS9	three	0.11	0.22	$=\omega_O$	$=\omega_O$	$=\omega_R$	0.26	-4840.988747	H5-H1	4.693	0.03029
										H5-H4	0.155	ns
H6	Orthologues & non-FA-Rosaceae vs. Rosoideae & Mal-MADS9 vs. Mal-MADS8	three	0.11	0.25	$=\omega_O$	$=\omega_O$	0.13	$=\omega_R$	-4840.132785	H6-H1	6.405	0.01138
										H6-H4	1.867	ns

$X^2_{(d=1, a=0.05)} = 3.841$

b

			average ω (dN/dS)							
ID	hypothesis description	ratio	Orthologues (O)	Rosoideae (R)	Amygdaleae (A)	Maleae	likelihood	comparison	2Δl	p-value
H0	all equal	one	0.13	$=\omega_O$	$=\omega_O$	$=\omega_O$	-4791.825707	na	na	na
H1	Orthologues vs. Rosaceae	two	0.11	0.21	$=\omega_R$	$=\omega_R$	-4783.574399	H1- H0	16.503	0.00005
H2	Orthologues vs. Rosoideae vs. Amygdaleae	three	0.11	0.23	0.13	$=\omega_A$	-4782.344698	H2- H1	2.459	ns
H3	Orthologues & Amygdaleae vs. Rosaceae	two	0.11	0.23	$=\omega_O$	$=\omega_R$	-4782.465184	H3- H0	18.721	0.00002

$X^2_{(d=1, a=0.05)} = 3.841$

c

			average ω (dN/dS)									
ID	hypothesis description	ratio	Orthologues (O)	Rosoideae (R)	Amygdaleae (A)	Gillenia	Maleae-MADS8	Maleae-MADS9	likelihood	comparison	2Δl	p-value
H0	all equal	one	0.12	$=\omega_O$	$=\omega_O$	$=\omega_O$	$=\omega_O$	$=\omega_O$	-4291.529772	na	na	na
H1	Orthologues vs. Rosaceae	two	0.10	0.17	$=\omega_R$	$=\omega_R$	$=\omega_R$	$=\omega_R$	-4287.753744	H1-H0	7.552	0.00599
H2	Orthologues vs. FA-Rosaceae vs. non-FA-Rosaceae	three	0.10	0.19	0.10	$=\omega_A$	$=\omega_R$	$=\omega_R$	-4286.695165	H2-H1	2.117	ns
H3	Orthologues & non-FA-Rosaceae vs. FA-Rosaceae	two	0.10	0.19	$=\omega_O$	$=\omega_O$	$=\omega_R$	$=\omega_R$	-4286.697304	H3-H0	9.665	0.00188

$X^2_{(d=1, a=0.05)} = 3.841$
